# Widespread thermophilization but weak link to climate warming in Europe’s summit plant communities

**DOI:** 10.1038/s41559-026-03150-x

**Published:** 2026-08-14

**Authors:** Johannes Hausharter, Johannes Wessely, Norbert Helm, Kryštof Chytrý, Karl Hülber, Dietmar Moser, Mary Carolina García Lino, Katharina Kagerl, Andrea Lamprecht, Jonathan Lenoir, Andreas Kollert, Andreas Mayr, Martin Rutzinger, Philippe Choler, Pieter De Frenne, Jonas J. Lembrechts, Otar Abdaladze, Chris Andrews, Peter Barančok, José-Luis Benito-Alonso, Eva M. Cañadas, Michele Carbognani, Pau Carnicero, Emmanuel Corcket, Michele Di Musciano, Jan Dick, Khatuna Gigauri, Andreas Hilpold, Juan J. Jiménez, Juan Lorite, Umberto Morra di Cella, Panagiotis Nyktas, Simone Orsenigo, Andrej Palaj, Martina Petey, Mihai Pușcaș, Christophe Randin, Ilektra Remoundou, Christian Rixen, Patrick Saccone, Angela Stanisci, Jean-Paul Theurillat, Tudor-Mihai Ursu, Thomas Vanneste, Raphael S. von Büren, Sonja Wipf, Manuela Winkler, Harald Pauli, Stefan Dullinger

**Affiliations:** 1 Department of Botany and Biodiversity Research, University of Vienna, Vienna, Austria; 2 Vienna Doctoral School of Ecology and Evolution, University of Vienna, Vienna, Austria; 3 GLORIA co-ordination, Institute for Interdisciplinary Mountain Research, Austrian Academy of Sciences, Vienna, Austria; 4 GLORIA co-ordination, Department of Ecosystem Management, Climate and Biodiversity, BOKU University, Vienna, Austria; 5 UMR CNRS 7058 “Ecologie et Dynamique des Systèmes Anthropisés” (EDYSAN), Université de Picardie Jules Verne, Amiens, France; 6 Department of Geography, University of Innsbruck, Innsbruck, Austria; 7 Université Grenoble Alpes, Université Savoie Mont Blanc, CNRS, LECA, Grenoble, France; 8 Forest & Nature Lab, Ghent University, Gontrode, Belgium; 9 Ecology & Biodiversity, Utrecht University, Utrecht, the Netherlands; 10 Plants & Ecosystems, University of Antwerp, Antwerp, Belgium; 11 Institute of Ecology, School of Natural Sciences and Medicine, Ilia State University, Tbilisi, Georgia; 12 UK Centre for Ecology and Hydrology, Penicuik, UK; 13 Institute of Landscape Ecology, Slovak Academy of Sciences, Bratislava, Slovak Republic; 14 Jolube Botanical Consultant, Jaca, Spain; 15 Department of Botany, University of Granada, Granada, Spain; 16 Department of Chemistry, Life Sciences and Environmental Sustainability, University of Parma, Parma, Italy; 17 Department of Botany, University of Innsbruck, Innsbruck, Austria; 18 Department of Animal Biology, Plant Biology and Ecology, Autonomous University of Barcelona, Bellaterra, Spain; 19 IMBE, Aix Marseille Université, Avignon Université, CNRS, IRD, Marseille, France; 20 Department of Life, Health and Environmental Sciences, University of L’Aquila, L’Aquila, Italy; 21 Department of Biological, Geological and Environmental Sciences (BiGeA), Alma Mater Studiorum, University of Bologna, Bologna, Italy; 22 Georgian Institute of Public Affairs, Tbilisi, Georgia; 23 Institute for Alpine Environment, Eurac Research, Bolzano, Italy; 24 Pyrenean Institute of Ecology, IPE, CSIC, Jaca, Spain; 25 Interuniversity Institute for Earth System Research, University of Granada, Granada, Spain; 26 Environmental Protection Agency of Aosta Valley, Saint Christophe, Italy; 27 Faculty of Geo-Information Science and Earth Observation (ITC), University of Twente, Enschede, the Netherlands; 28 Natural History Museum of Crete, School of Sciences and Engineering, University of Crete, Heraklion, Greece; 29 Department of Earth and Environmental Sciences, University of Pavia, Pavia, Italy; 30 A. Borza Botanic Garden, Babeș-Bolyai University, Cluj-Napoca, Romania; 31 Faculty of Biology and Geology, Babeș-Bolyai University, Cluj-Napoca, Romania; 32 Emil G. Racoviță Institute, Babeș-Bolyai University, Cluj-Napoca, Romania; 33 Institute of Earth Surface Dynamics (IDYST), University of Lausanne, Lausanne, Switzerland; 34 Fondation du Jardin botanique Flore-Alpe, Champex-Lac, Switzerland; 35 Interdisciplinary Centre for Mountain Research (CIRM), University of Lausanne, Bramois, Switzerland; 36 Mediterranean Agronomic Institute of Chania, Chania, Greece; 37 WSL Institute for Snow and Avalanche Research SLF, Davos, Switzerland; 38 Climate Change, Extremes and Natural Hazards in Alpine Regions Research Center CERC, Davos, Switzerland; 39 Institute of Botany of the Czech Academy of Sciences, Průhonice, Czech Republic; 40 Department of Biosciences and Territory, University of Molise, Termoli, Italy; 41 Department of Plant Sciences, University of Geneva, Chambésy, Switzerland; 42 Institute of Biological Research Cluj, National Institute of Research and Development for Biological Sciences, Cluj-Napoca, Romania; 43 Meise Botanic Garden, Meise, Belgium; 44 Department of Research and Monitoring, Swiss National Park, Zernez, Switzerland; 45 Department of Environmental Sciences, Research Group Ecology, University of Basel, Basel, Switzerland

**Keywords:** Climate-change ecology, Plant ecology, Macroecology

## Abstract

Recent changes in alpine vegetation are often attributed to climate warming, particularly community composition shifts towards more warmth-associated species, or thermophilization. Here we assess the link between thermophilization and warming across 53 European mountain summits. We combine long-term macroclimatic and microclimatic temperature time series with vegetation surveys in 724 permanent plots, monitored over 21 years, to evaluate a possible thermophilization signal and relate it to rates of change in 10 temperature metrics. We find evidence of both thermophilization of alpine plant communities and an increase in temperatures. However, although these two trends are related when averaged across mountain regions, their relationship is weak at the individual plot scale, especially when considering microclimatic temperature metrics. Instead, substrate conditions and particularly the availability of thermophilic colonizers in the surrounding vegetation have a major influence on plot-level thermophilization rates. We conclude that the response of plant communities to climate change strongly depends on the abiotic and biotic context, and intensified monitoring efforts are needed to reduce the resulting uncertainties.

## Abstract (web summary)

Attributing alpine vegetation changes to climate change alone is intuitive but might neglect complexities such as microclimate and dispersal limitations. Here the authors show that thermophilization of European summit vegetation is weakly related to temperature change, as it depends on local environmental and floristic conditions.

## Main

Plant communities above the alpine treeline host a high proportion of endemic or small-ranged taxa^1,2^ and provide substantial ecosystem services^3,4^. Over the last decades, however, these communities have undergone considerable changes^5–7^. These changes are frequently attributed to warming temperatures, which are supposed to allow for the colonization by more warmth-associated species. Indeed, phenomena such as upward shifts of the elevational ranges of species^8,9^, increasing species richness on summits^7,10^ or shifts in community composition towards more warmth-associated species^6^ intuitively appear linked to increasing temperatures as a main driver. However, explicit assessments of the strength of the relationship between the magnitude of warming and the rates of these processes are still rare (for example, refs. ^6,11^) and the assumed impact of climate warming thus remains poorly substantiated^12,13^.

Demonstrating direct links between vegetation change and climatic trends faces several challenges. First, many studies documenting recent alpine vegetation changes focused on a limited spatial extent, such as a single mountain range, or even a single mountain (for example, refs. ^8,14–17^). Within limited spatial extents, the variation in the rate of climatic change is limited and potential relationships with vegetation change are hence difficult to detect. Second, ‘temperature’ is a variable that can be summarized in several ways, for example as thermal sums or as maxima, means or minima, during various seasonal periods, for air or soil conditions and so on^18,19^. The magnitude, and even the direction of change over time in these temperature metrics may differ considerably^20^. Consequently, variable selection and the choice between considering a single metric or several, potentially interacting, metrics may thus have a strong effect on the observed relationship between temperature change and vegetation change^18^. Third, temperature, and therefore temperature change, is usually interpolated from weather station data 2 m above ground. This focus on macroclimatic ambient air temperature has been argued to be at odds with the way temperature affects low-stature alpine plant communities^21^. These communities are rather shaped by the near-ground microclimate, which can deviate considerably from the macroclimate in the rugged alpine terrain^22^ and may also buffer against macroclimatic temperature change^23^. Fourth, given that plant communities can exhibit delayed responses to changing temperatures, considering appropriate lag times may be essential when investigating relationships between the two^24^. Accordingly, a conclusive temporal analysis of the link between temperature change and vegetation change probably requires a sufficiently long time series of on-site microclimate measurements alongside vegetation monitoring, ideally over a large geographical extent with varying trajectories of temperature change, expressed in several temperature metrics.

Finally, substantiating the link between climate change and alpine vegetation change is complicated by a wide range of potentially confounding factors. Besides additional components of anthropogenic global change, such as land-use change^25^ or airborne nitrogen deposition^26^, non-climatic site conditions (soil conditions and disturbances)^27,28^, biotic interactions and community properties^29^, as well as neutral processes^30–32^, codetermine the dynamics of alpine vegetation to varying extents. Some of these non-climatic factors may compound, mitigate or even mask the effects of climate change. As an example, dispersal and recruitment limitations, which are widespread in alpine vegetation^24,32^, may make colonization of sites strongly dependent on nearby source pools of potential colonizers; or specific substrate conditions, such as rock and scree, may filter potential colonizers independent of their thermal requirements^33^.

One prominent aspect of alpine vegetation change with a particularly intuitive link to increasing temperatures is the so-called thermophilization of plant communities (sensu ref. ^6^). Thermophilization describes an increase in the ratio of warmth-associated to cold-associated species. It involves the colonization by or a gain in cover of warmth-associated species and/or the disappearance or loss in cover of cold-associated species. There is clear evidence for such directional shifts, although with considerable regional or even local-scale variation^6,11,16,34^. As with other aspects of alpine vegetation change, there are, however, hardly any studies that have evaluated the intuitive hypothesis that higher rates of warming will result in more pronounced thermophilization rates. The lack of such studies hampers our understanding of how the changing climate affects alpine vegetation and increases the uncertainty of vegetation and biodiversity scenarios under further twenty-first century climate change^35^.

The GLORIA-Europe network, to our knowledge the largest systematic monitoring scheme of climate change effects on mountain summit vegetation^36^, offers a dataset particularly suited for closing this research gap. Within this network, the species composition in hundreds of local vegetation plots has been monitored every 7 years since the year 2001. Their distribution across all major European mountain ranges results in considerable variation in temperature trends, which are recorded on-site. The summits were selected such that the impact of land use, arguably the most important other driver of recent alpine vegetation change, is minimized^37^. Here we first assessed whether the monitored plant communities exhibited a thermophilization signal and how it varied across the monitoring sites. Second, we related thermophilization rates to the rates of temperature change, assessed through both interpolated macroclimatic air temperature and microclimatic on-site soil temperature. Third, we evaluated the potential impacts of other non-climatic environmental properties on thermophilization rates and compared them to the effects of temperature change. We hypothesize that (1) European summit plant communities experienced significant thermophilization over 21 years. We further expect that the thermophilization rates (2) generally increased with the rate of warming, although effect sizes may differ among temperature metrics; and (3) were more strongly related to microclimatic (on-site soil) temperature trends than to macroclimatic (interpolated air) temperature trends. We further assume to find additional effects of the biotic and abiotic context on thermophilization rates. In particular, we expect that thermophilization (4) increased with the availability of thermophilic colonizers in the immediate surroundings of the vegetation plots and the proportion of bare soil available for colonization within plots; and that it (5) decreased with unsuitable soil conditions, specifically a high proportion of rock and scree covering the plots.

## Results

### Quantifying thermophilization and environmental drivers

**Fig. 1:**
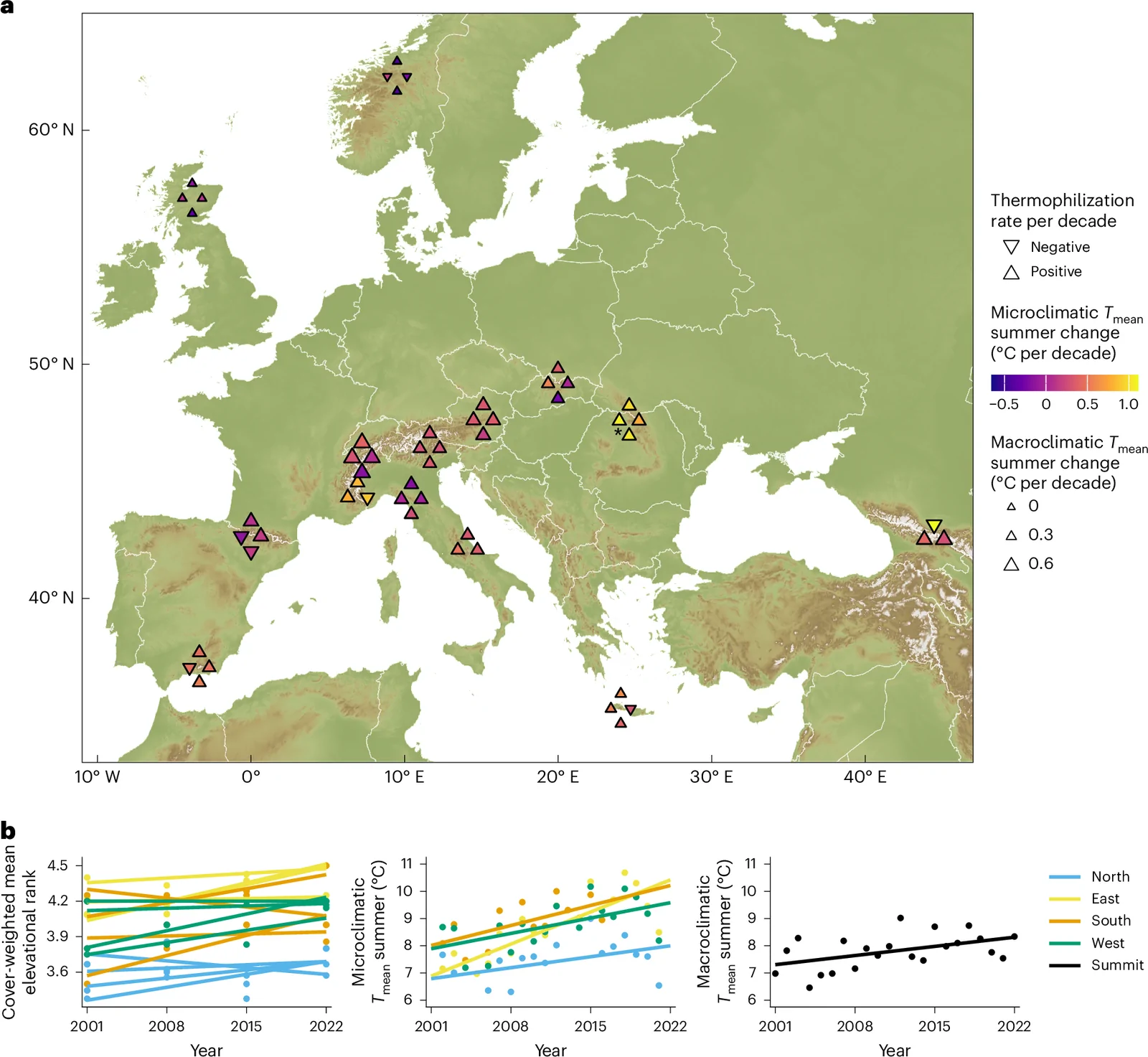
Map of surveyed summits and example trends in thermophilization and temperature change. **a**, Geographical distribution of the 53 summits surveyed across the 14 mountain regions in Europe. Triangle orientation indicates whether the thermophilization rates (plot-level changes in mean elevational rank of the taxa) per decade, averaged across all plots of a summit, were positive (pointing up) or negative (pointing down). The colour scale indicates the rate of microclimatic mean summer temperature change per decade (derived from soil temperature measurements) averaged across all plots of a summit; symbol size scales with the summit rate of macroclimatic mean summer temperature change per decade (derived from interpolated air temperatures). Note that summits are jittered around the centroids of mountain regions for better readability (with summit arranged by decreasing elevation in the order: top, left, right, bottom). Asterisk (*) indicates Eastern Carpathians, Rodna Mountains–Gropile. **b**, Thermophilization rates (left), rates of microclimatic (centre; from the four soil temperature loggers per summit) and macroclimatic (right; at the summit level) mean summer temperature change are illustrated for one exemplary summit (location on the map indicated by an asterisk). The colour indicates the exposition (North, East, South or West) of the plots or loggers. The rates were derived by regressing the cover-weighted mean elevational ranks of plots, the logger-measured mean summer temperatures and the interpolated mean summer temperatures of summits, against time. The raw values that were regressed against time are depicted as points. Positive trends (increases) in the cover-weighted mean elevational rank indicate thermophilization. For the map in **a**, background elevation data were obtained from MERIT DEM^65^ and country borders were obtained from GADM under a Creative Commons license CC BY-SA 2.0.

We relied on data from 724 vegetation plots arranged in plot clusters (that is, composed of four 1 m × 1 m plots) in each of the four cardinal directions (for a total of 16 vegetation plots per summit; Supplementary Fig. 1) across 53 summits in 14 mountain regions distributed across Europe (Fig. 1 and Supplementary Table 1; note that not all 16 plots of every summit could be considered for this study; Methods). These plots were surveyed four times between 2001 and 2022, and soil temperature was measured in hourly intervals at the centre of each plot cluster during the same period. In every survey, the percentage cover of all vascular plant taxa occurring in the plots was recorded, and all taxa occurring in the uppermost 10 m of the summit were listed. Additionally, for each plot, the percentage area that was covered by rock and scree, or bare soil, was estimated. Each taxon occurring in any of the plots was assigned an elevation rank expressing its elevational distribution optimum^6^ (representing the elevational vegetation belt (from colline (7) to nival (1))—in which the taxon occurs most frequently; Supplementary Table 2), which we consider a proxy for the temperature niche optimum of the taxon. For each plot in each survey, we then calculated the cover-weighted mean elevational rank across all co-occurring taxa and regressed these against time to derive thermophilization rates per decade. The resulting rates were tested for deviation from zero by means of a linear mixed-effects model with the intercept as the only fixed effect. Similarly, we derived rates of plot-cluster-level microclimatic change (derived from soil temperature measurements) and summit-level macroclimatic change (derived from interpolated air temperatures) in ten temperature metrics, for a total of 20 rates of temperature change which we tested for deviation from zero with the same type of linear mixed-effects model as described above. Among possible metrics, we calculated mean annual temperatures because it integrates over several, probably important, temperature variables, as well as mean, minimum and maximum summer (May to September; growing season conditions), winter (November to March; conditions during dormancy) and June temperatures (previously shown to correlate with thermophilization by ref. ^6^; see Supplementary Table 3 for an overview of temperature metrics used). We again used linear mixed-effects models, but now with temperature metrics as fixed-effect predictors, to assess the relationship between the magnitude of warming and thermophilization. As vegetation change can lag behind temperature change^24^, we repeated these analyses for 1–10 years of assumed lag time between temperature change and thermophilization, but only for macroclimatic metrics, as microclimatic data were only available from the year 2001 onwards. For each plot we further calculated the difference between the mean elevational ranks of taxa occurring in the uppermost 10 m of the summit and the mean elevational rank of taxa in the focal plot during the first survey. This difference provides a proxy for the availability of thermophilic colonizers in the surroundings of the plots. Finally, we added this proxy of potentially available colonizers together with the proportions of bare soil, as well as rock and scree cover per plot as further fixed-effect predictors to the linear mixed-effects models relating thermophilization rates to rates of change in the different temperature metrics.

### Overall trends in thermophilization rates and temperature change

**Fig. 2:**
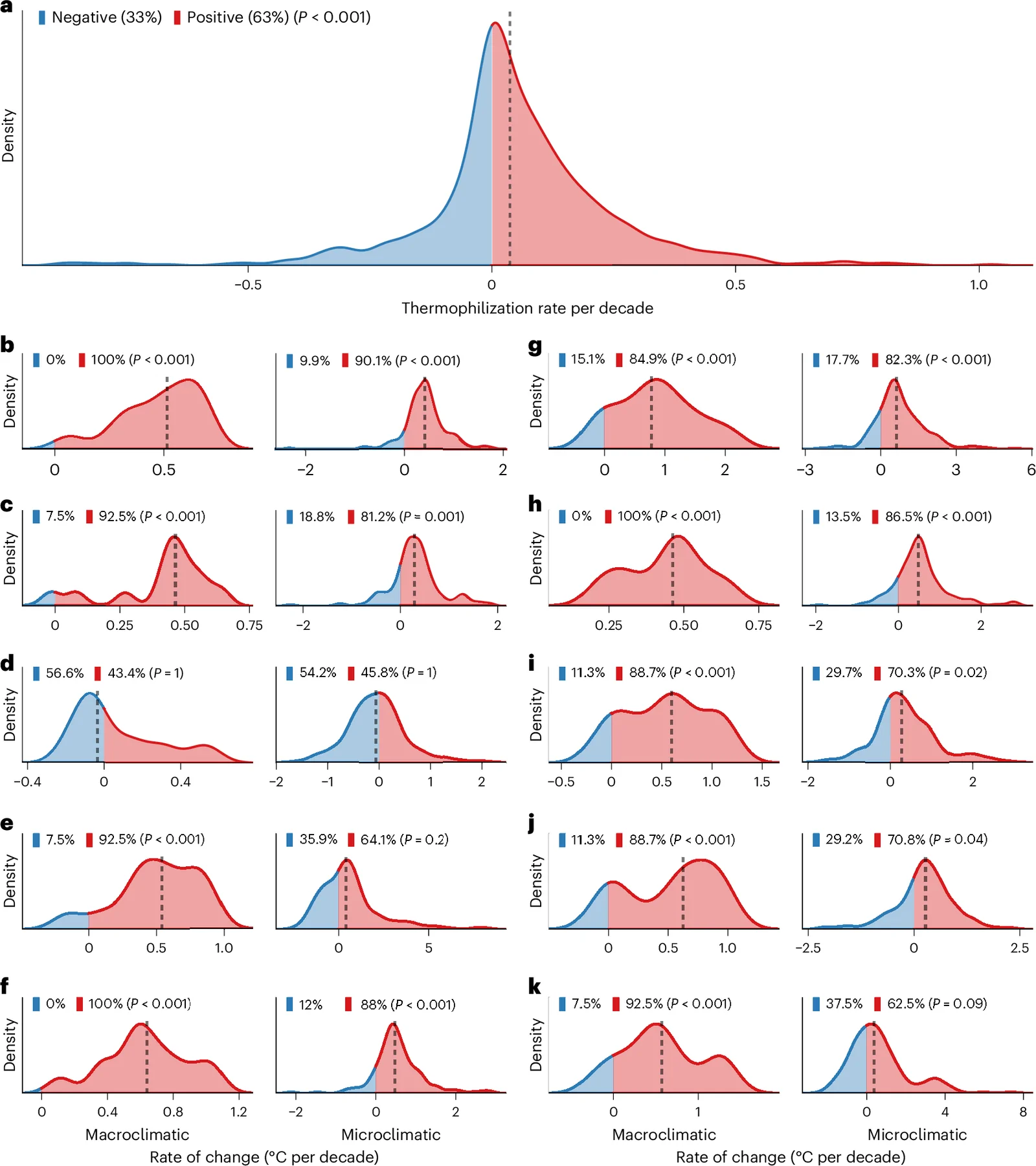
Distribution of thermophilization rates and rates of temperature change. **a**, Distribution of the plot-level thermophilization rates (change in cover-weighted mean elevational rank; *n* = 724 plots). **b**–**k**, Rates of change for ten summit-level macroclimatic (left; *n* = 53 summits) and ten plot-cluster-level microclimatic (right; *n* = 192 plot clusters) temperature metrics (*T*_mean_ annual (**b**), *T*_mean_ summer (**c**), *T*_min_ summer (**d**), *T*_max_ summer (**e**), *T*_mean_ winter (**f**), *T*_min_ winter (**g**), *T*_max_ winter (**h**), *T*_mean_ June (**i**), *T*_min_ June (**j**) and *T*_max_ June (**k**)). Percentages indicate proportions of negative (blue) and positive (red) rates. The dashed lines indicate the respective medians. Values in **a** do not sum up to 100% because of plots with thermophilization rates of zero (4%). One-sided *P* values were derived from intercept-only linear mixed-effects models testing whether thermophilization rates and rates of temperature change were significantly greater than zero. The *P* values were further corrected for multiple comparisons (this adjustment can lead to *P* values of exactly 1) and are presented at the top of each panel. *T*_mean_, mean temperature; *T*_min_, minimum temperature; *T*_max_, maximum temperature.

Across all 724 permanent vegetation plots, intercept-only linear mixed-effects models revealed that the mean thermophilization rate per decade was positive (0.06; s.d. = 0.20, minimum −0.88, maximum 1.02) and differed significantly from zero (*z* = 3.09, *P* < 0.001; Supplementary Table 4). This rate implies that it would take approximately 170 years for a complete turnover of the community composition of vegetation plots typical for one elevational belt (for example, subnival) towards a composition typical of the next-lower vegetation belt (in this example, alpine). Of the 724 plots, 63% showed a positive thermophilization rate, whereas 33% showed a negative rate and 4% a rate of zero (Fig. 2a). The thermophilization rate was independent of geographical latitude or elevation (Supplementary Table 5).

**Extended Data Fig. 1:**
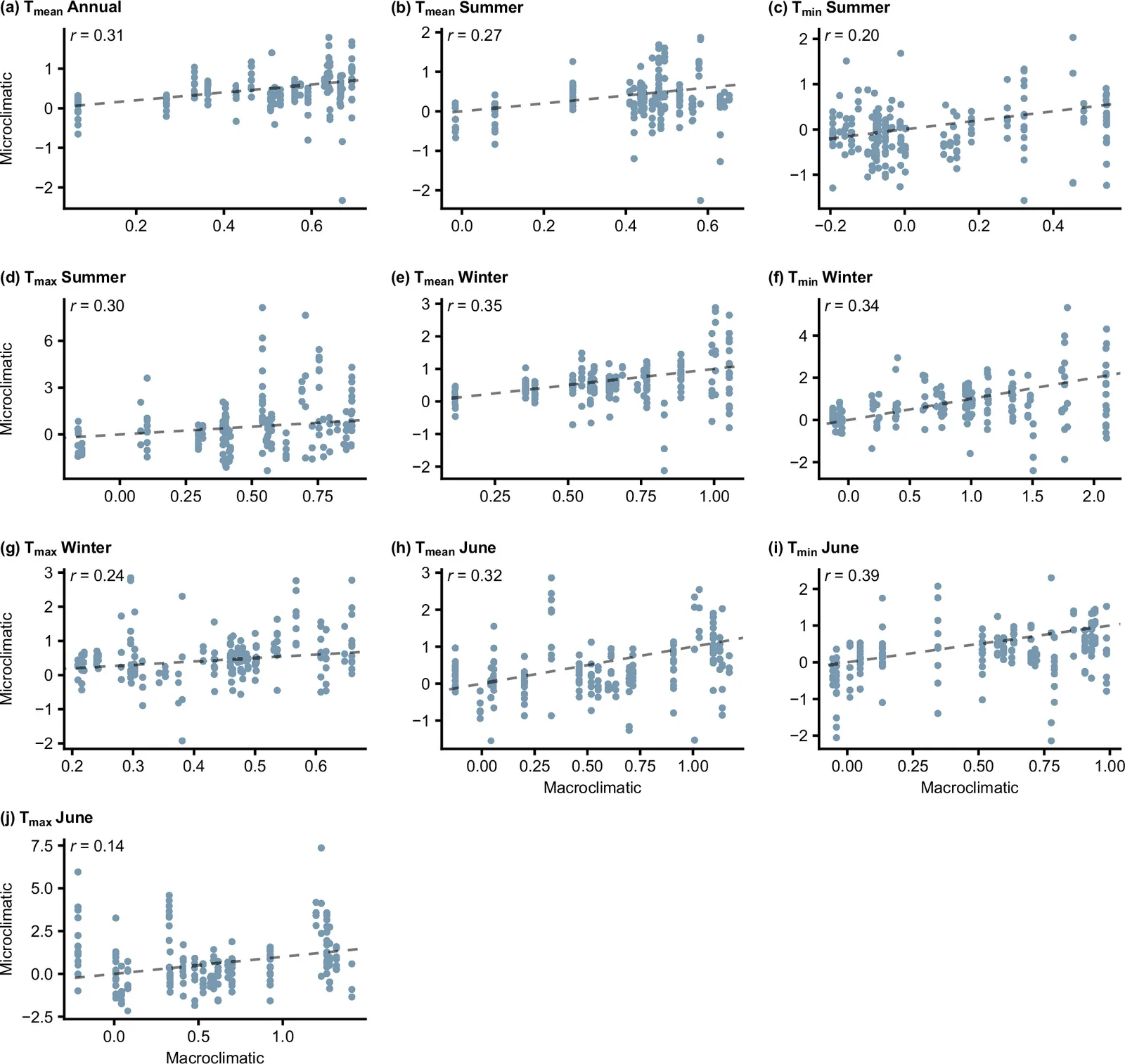
Relationships between macroclimatic and microclimatic rates of change in the same temperature metrics. Relationships between macroclimatic and microclimatic rates of change in the same temperature metrics across the 724 vegetation plots for (**a**) mean annual temperature, (**b**) mean summer temperature, (**c**) minimum summer temperature, (**d**) maximum summer temperature, (**e**) mean winter temperature, (**f**) minimum winter temperature, (**g**) maximum winter temperature, **(h**) mean June temperature, (**i**) minimum June temperature and (**j**) maximum June temperature. The dashed lines represent the idealized 1:1 relationships. Pearson correlation coefficients (*r*) are provided in the panels’ top-left corners. T_mean_ … mean temperature; T_min_ … minimum temperature, T_max_ … maximum temperature.

**Extended Data Fig. 2:**
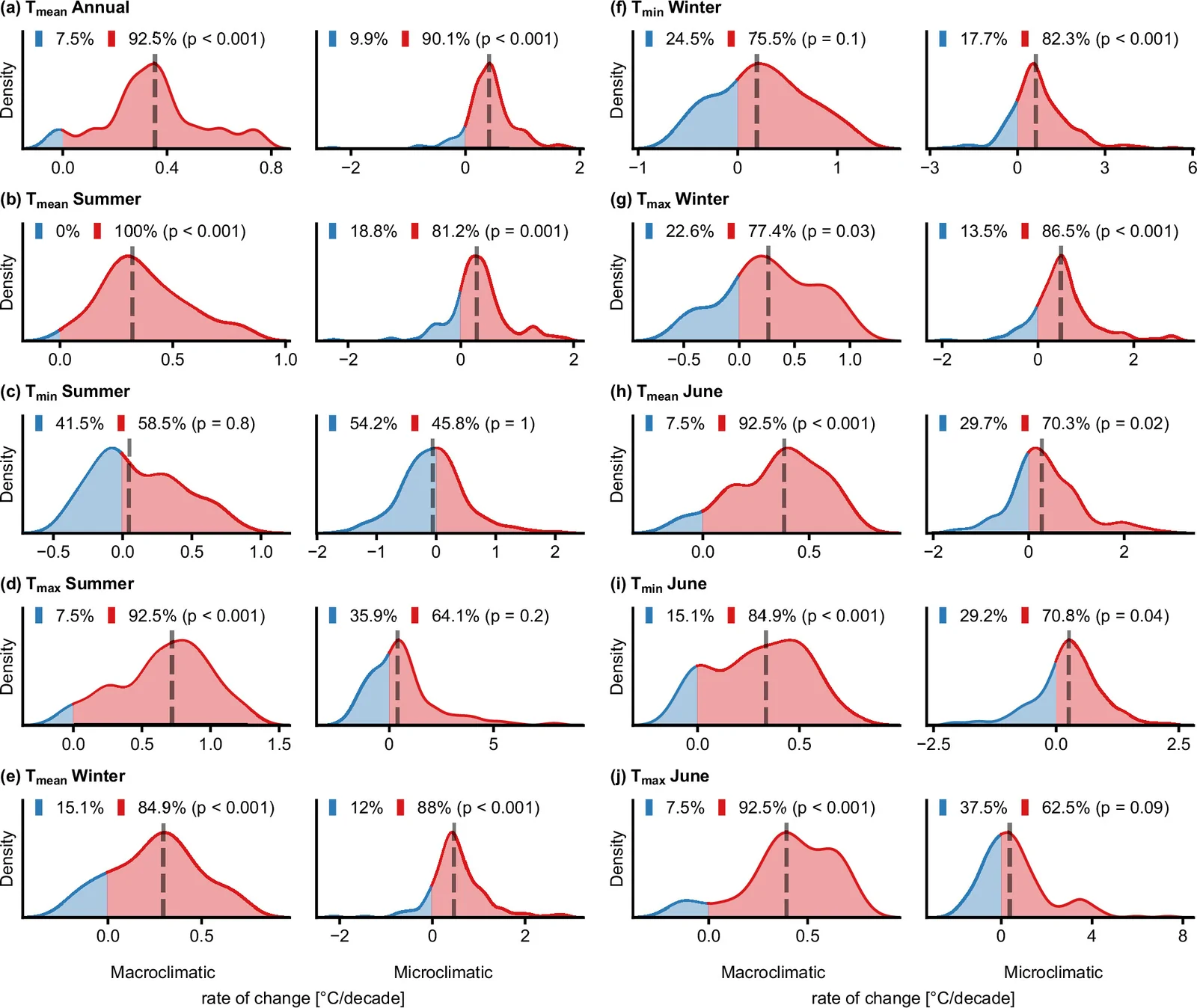
Distribution of rates of temperature change when macroclimatic temperature metrics are calculated with a 4-year time lag. Distribution of rates of change for ten summit-level macroclimatic (left, calculated with a 4-year time lag; *n* = 53 summits) and plot-cluster-level microclimatic (right, as already presented in the main text; *n* = 192 plot clusters) temperature metrics (**[a]** T_mean_ annual, **[b]** T_mean_ summer, **[c]** T_min_ summer, **[d]** T_max_ summer, **[e]** T_mean_ winter, **[f]** T_min_ winter, **[g]** T_max_ winter, **[h]** T_mean_ June, **[i]** T_min_ June and **[j]** T_max_ June). Percentages indicate proportions of negative (blue) and positive (red) rates. The dashed lines indicate the respective medians. One-sided p values were derived from intercept-only linear mixed-effects models testing whether rates of temperature change were significantly greater than zero. The p values were further corrected for multiple comparisons (this adjustment can lead to p values of exactly 1) and are presented at the top of each panel. T_mean_ … mean temperature; T_min_ … minimum temperature, T_max_ … maximum temperature.

This overall thermophilization signal in alpine plant communities was consistent with the signal we found for the rates of change in both macroclimatic and microclimatic temperatures. The rates of temperature change per decade were positive and differed significantly from zero for all temperature metrics except for macroclimatic and microclimatic minimum summer temperature, as well as microclimatic maximum summer and maximum June temperatures (Supplementary Table 4). Rates of change in the macroclimatic temperature metrics were on average higher than in microclimatic ones with mean rates, across all ten metrics of 0.51 ± 0.32 °C per decade and 0.44 ± 0.85 °C per decade, respectively. Depending on the temperature metric, between 45.8% and 90.1% (mean 74.2%) of plot clusters (microclimate) and between 43.4% and 100% (mean 88.3%) of summits (macroclimate) showed positive rates of temperature change (Fig. 2b–k). Macroclimatic and microclimatic rates of change in the same temperature metrics correlated only weakly among each other, with Pearson correlation coefficients ranging from 0.14 to 0.39 (Extended Data Fig. 1). Macroclimatic temperature trends were similar when calculated under an assumed lag of thermophilization response, that is when calculated for time intervals preceding the vegetation monitoring by 1–10 years (Extended Data Fig. 2 and Supplementary Table 6).

### Relationships between thermophilization rates and the rates of temperature change

**Fig. 3:**
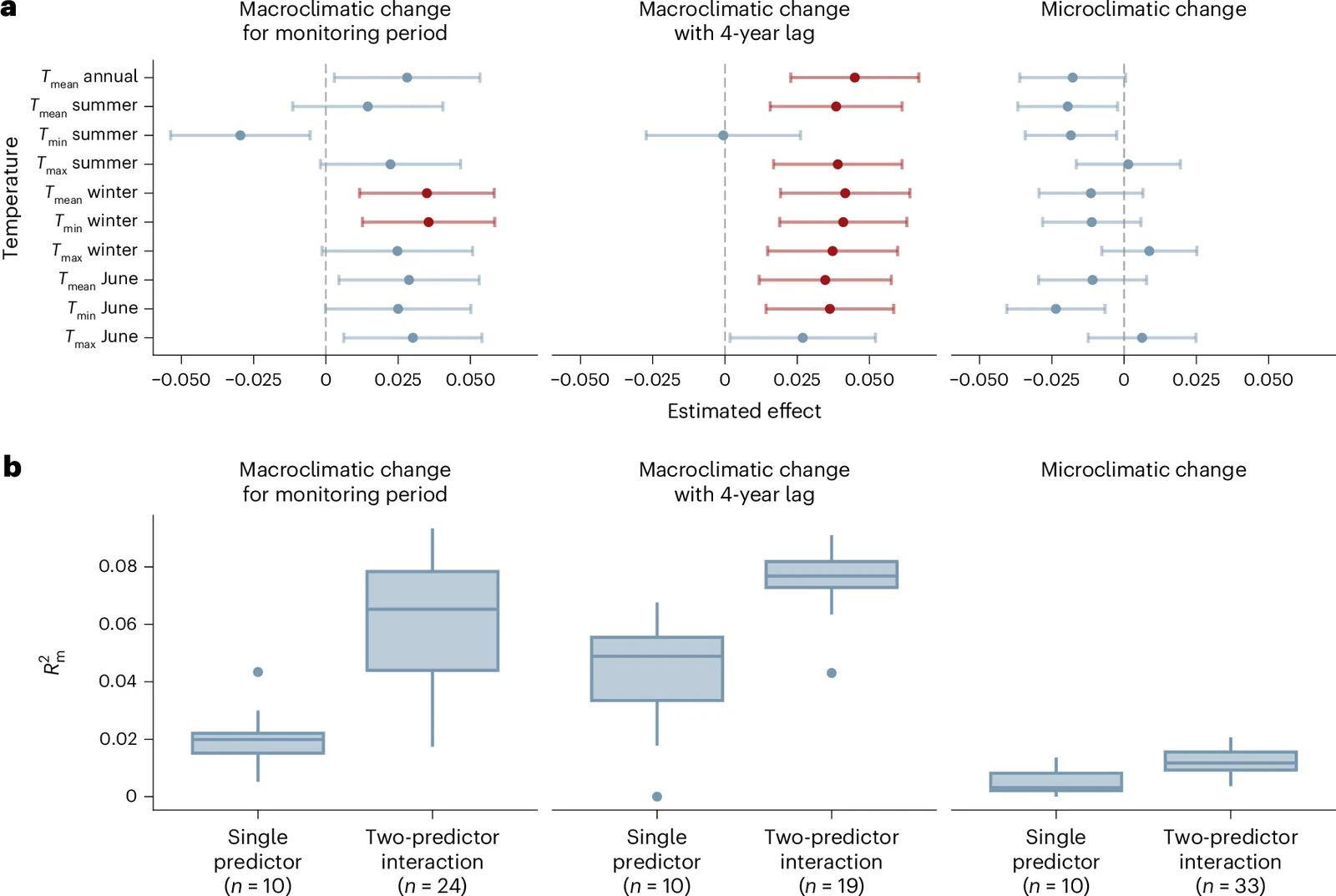
Results of linear mixed-effects models relating thermophilization rates to rates of temperature change. **a**, Estimated effects (dots) and their 90% confidence intervals (error bars) of linear mixed-effects models relating the thermophilization rates individually to the different rates of change in each of ten temperature metrics on different climatic scales. Temperature metrics with a significant positive effect on the thermophilization rates (after correcting for multiple comparisons) are highlighted in red. Dashed lines indicate 0. **b**, *R*^2^_m_ values of models containing either one of macroclimatic or microclimatic temperature metrics (single predictor) or two interacting temperature metrics (two-predictor interaction). Boxes indicate the median and interquartile range (IQR; 25th and 75th percentiles), whiskers extend to the furthest data points within 1.5× IQR from the hinges and points represent outliers.

When calculated for the time interval of vegetation monitoring (2001–2022) single-predictor linear mixed-effects models revealed a significant relationship only between thermophilization rates and two macroclimatic metrics: mean and minimum winter temperature change (Fig. 3a), with only weak explanatory power, as quantified by the marginal *R*^2^ (*R*^2^_m_) of 0.03 and 0.04, respectively. Microclimatic metrics explained a still lower fraction of the observed variation in thermophilization (average *R*^2^_m_ across all macroclimatic and microclimatic models were 0.02 and <0.01, respectively; Fig. 3b and Supplementary Table 7).

**Extended Data Fig. 3:**
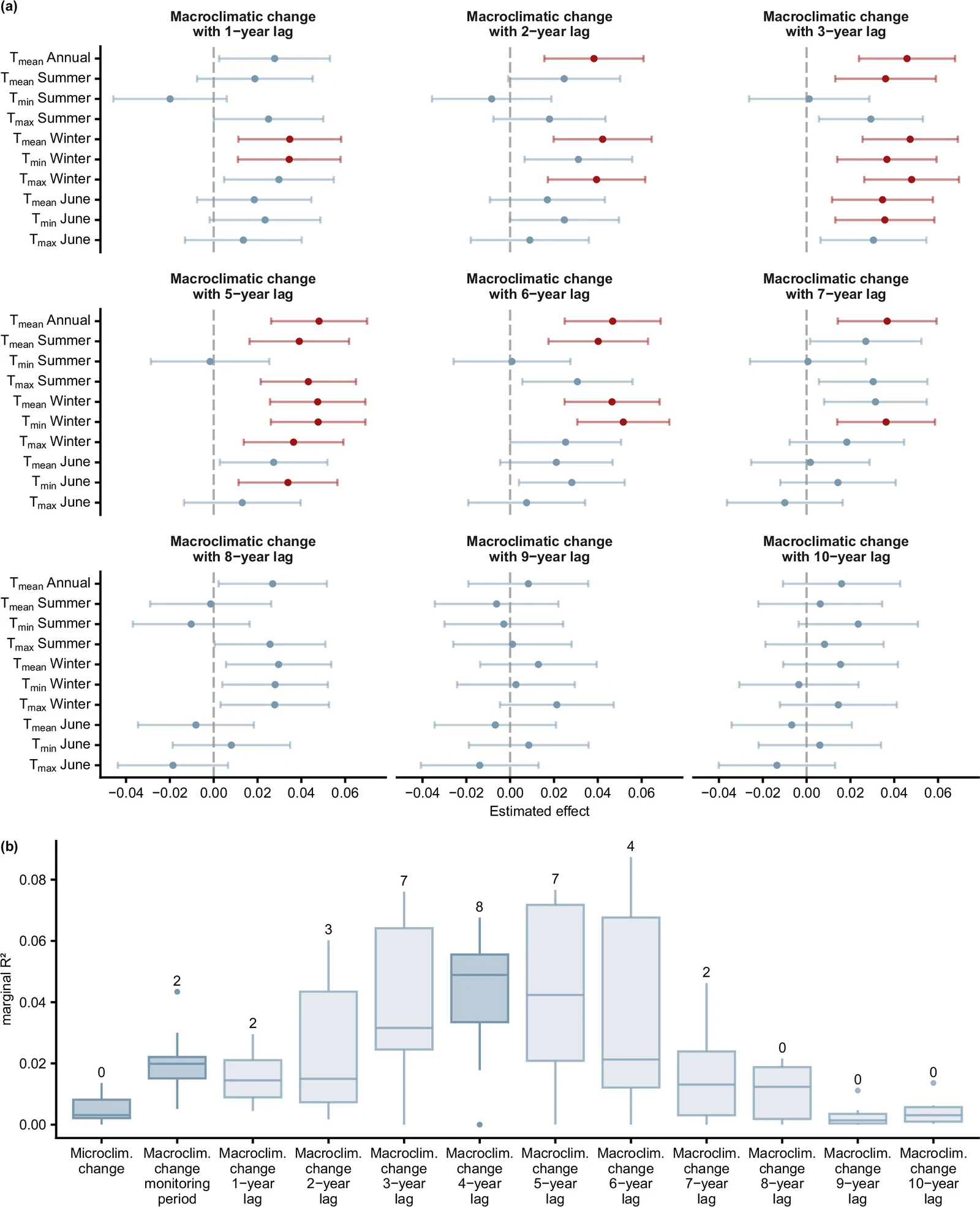
Results of linear mixed-effects models relating thermophilization rates to rates of macroclimatic temperature change calculated with time lags of one to ten years. (**a**) Estimated effects (dots) and their 90% confidence intervals (error bars) of linear mixed-effects models relating thermophilization rate individually to the rates of change in each of ten macroclimatic temperature metrics calculated with different time lags that are not presented in the main text. Temperature metrics with a significant effect on the thermophilization rate (after correcting for multiple comparisons) are highlighted in red. Dashed lines indicate 0. Estimated effects are based on models fitted with the rates of temperature change scaled to zero mean and unit variance for better comparability. (**b**) Marginal R^2^ (R^2^_m_) values of models containing either one of the macroclimatic or microclimatic temperature metrics (n = 10). The numbers above the individual boxes indicate the number of temperature metrics that were significant predictors of thermophilization rates. R^2^_m_ values already presented in the main text are also included here for comparison and highlighted in a darker color. Boxes indicate the median and interquartile range (IQR; 25th and 75th percentiles), whiskers extend to the furthest data points within 1.5 × IQR from the hinges, and points represent outliers.

Accounting for possible lag times of vegetation responses by calculating macroclimatic temperature change rates using one-year sliding time windows preceding the monitoring interval by 1–10 years (that is, for the periods 2000–2021 to 1991–2012), revealed that temperature trends preceding the monitored vegetation changes by 3–5 years were better coupled with thermophilization rates than temperature trends calculated for the same time window as the monitored vegetation changes (Extended Data Fig. 3). For the best-fitting 4-year time lag, changes in eight of the ten temperature metrics were significantly related to thermophilization rates, although the explanatory power remained weak (average *R*^2^_m_ = 0.04; Fig. 3b).

Finally, to explore whether combinations of different metrics of temperature improve the relationship between the magnitude of warming and thermophilization rates, we combined two such metrics and their interaction term as fixed effects into linear mixed-effects models. We only considered interactions between variables on the same climatic scale (that is, only between either macroclimatic or microclimatic variables, but not between one macroclimatic and one microclimatic variable) and between variables that were not highly correlated (|Pearson’s *r* | ≤ 0.5). These combined metrics of temperature change captured a higher proportion of the observed variation in thermophilization rates than did single predictors (Fig. 3b), but their explanatory power remained moderate to weak, especially in the case of microclimatic change (average *R*^2^_m_ across all two-metric macroclimatic and microclimatic models were 0.06 and 0.01, respectively), and few interactions had a significant effect (Supplementary Tables 8–10). Optimized two-metric macroclimatic models with a 4-year lag time reached a mean *R*^2^_m_ of 0.08 (Fig. 3b).

**Fig. 4:**
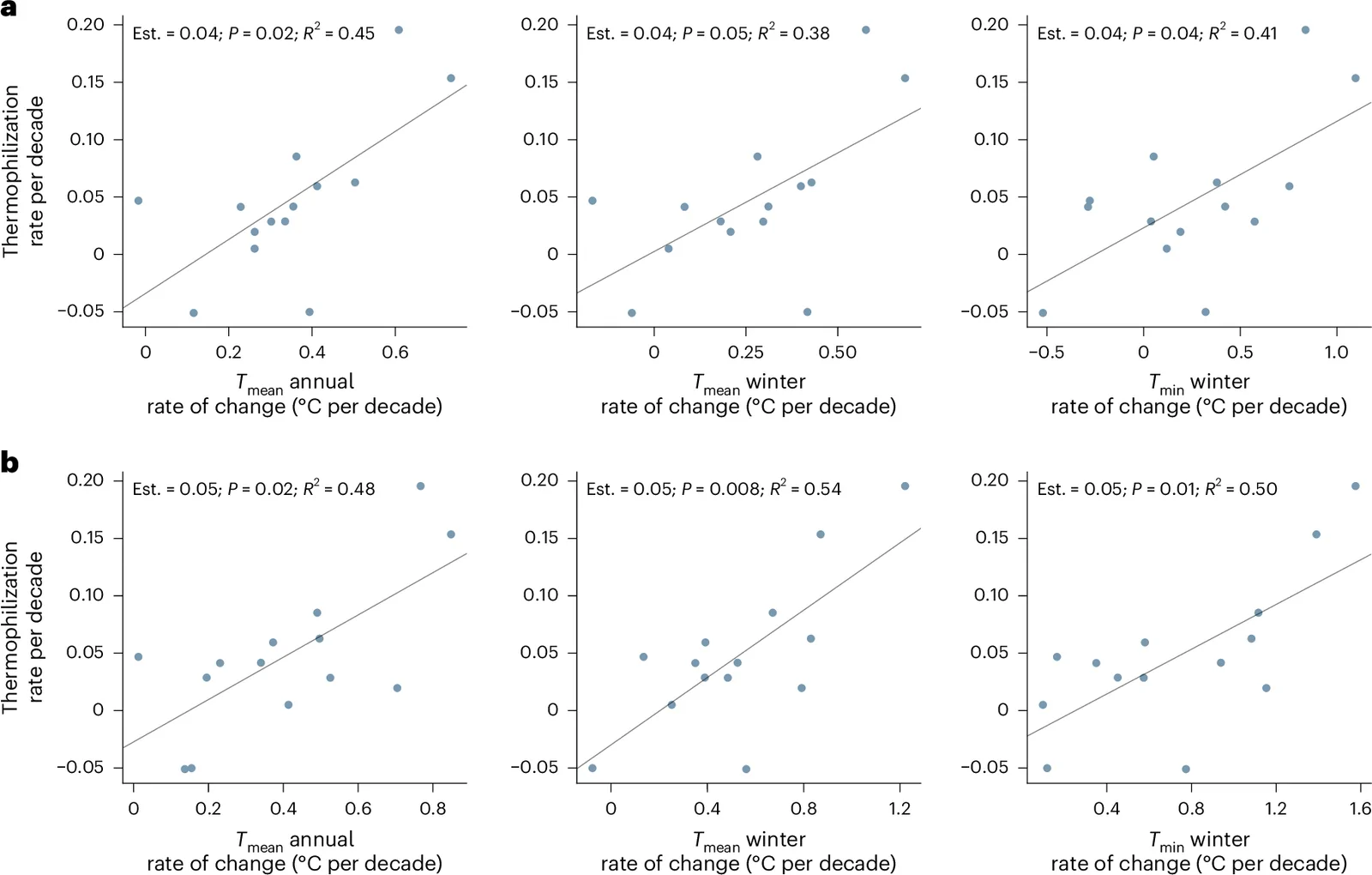
Relationships between thermophilization rates and rates of temperature change on the regional level. **a**,**b**, Relationships between thermophilization rates and rates of temperature change when aggregated to the regional scale for macroclimatic temperature change metrics calculated with a 4-year time lag (**a**) and microclimatic temperature change metrics (**b**). Only significant relationships as determined by OLS regressions are presented here, with estimated effects (Est.), one-sided, right-tailed *P* values corrected for multiple comparisons and the models *R*^2^ (proportion of explained variance) provided in the top left. The lines represent the regression lines. The estimated effects provided are based on models fitted with the rates of temperature change scaled to zero mean and unit variance for better comparability. The unscaled data are depicted for better interpretability.

**Extended Data Fig. 4:**
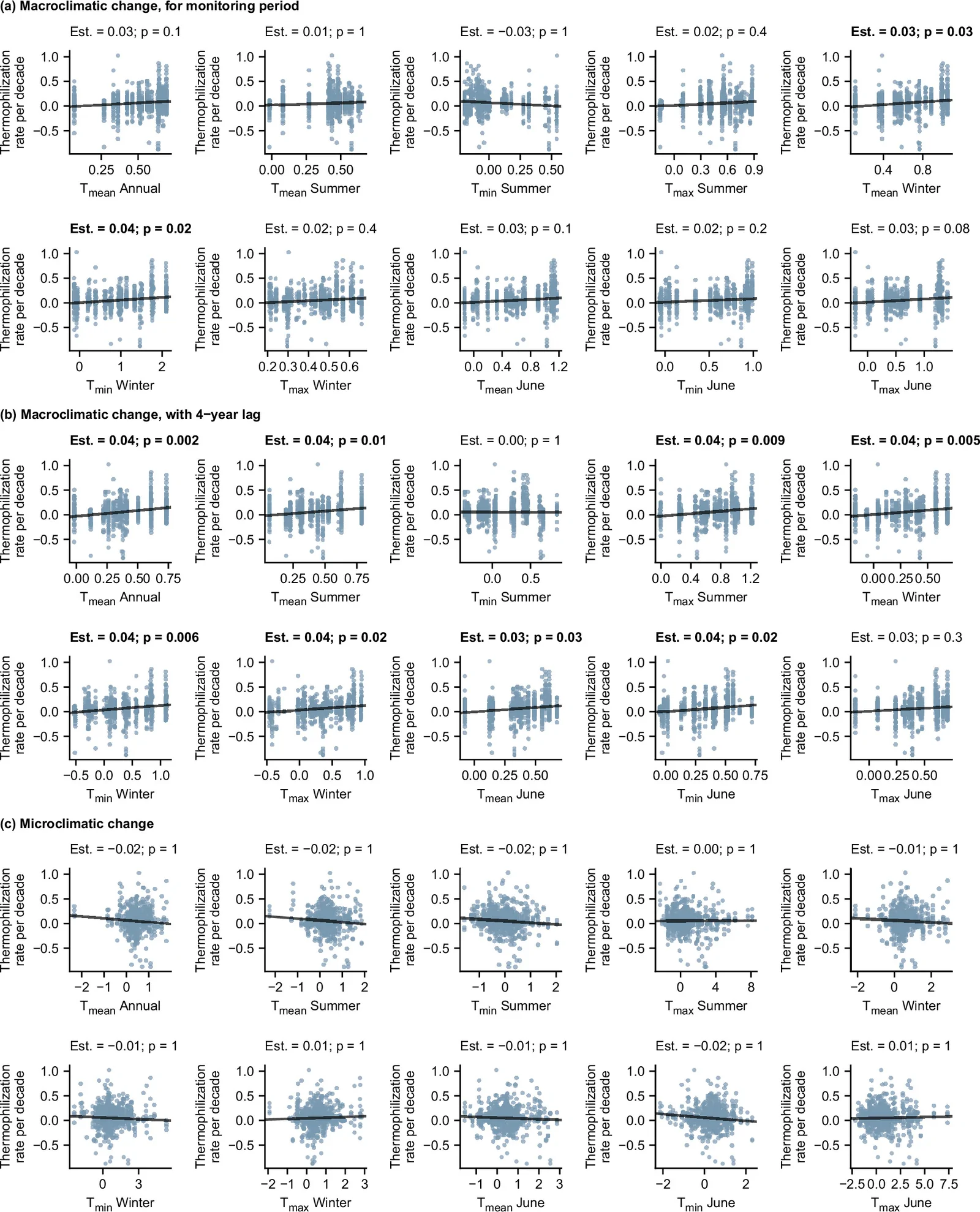
Relationships between thermophilization rates and rates of temperature change on the plot level. Relationships between plot-level thermophilization rate and the rates of change in ten (**a-b**) summit-level macroclimatic (calculated for the monitoring period [**a**] or with a 4-year time lag [**b**]) and (**c**) plot-cluster-level microclimatic temperature metrics across the 724 vegetation plots. Estimated effect (Est.) and p value of the respective temperature metric, derived from linear mixed-effect models, are provided above each panel. Only the one-sided, right-tailed p values adjusted for multiple comparisons are provided (this adjustment can lead to p values of exactly 1). Bold text indicates statistically significant p values. The models included a random-intercept term for plot cluster nested in summit nested in mountain region. The line represents the regression line of the fitted models. The estimated effects are reported based on models fitted with predictors scaled to zero mean and unit variance, while the unscaled data is depicted for better interpretability. Est. … estimated effect; T_mean_ … mean temperature; T_min_ … minimum temperature, T_max_ … maximum temperature.

**Extended Data Fig. 5:**
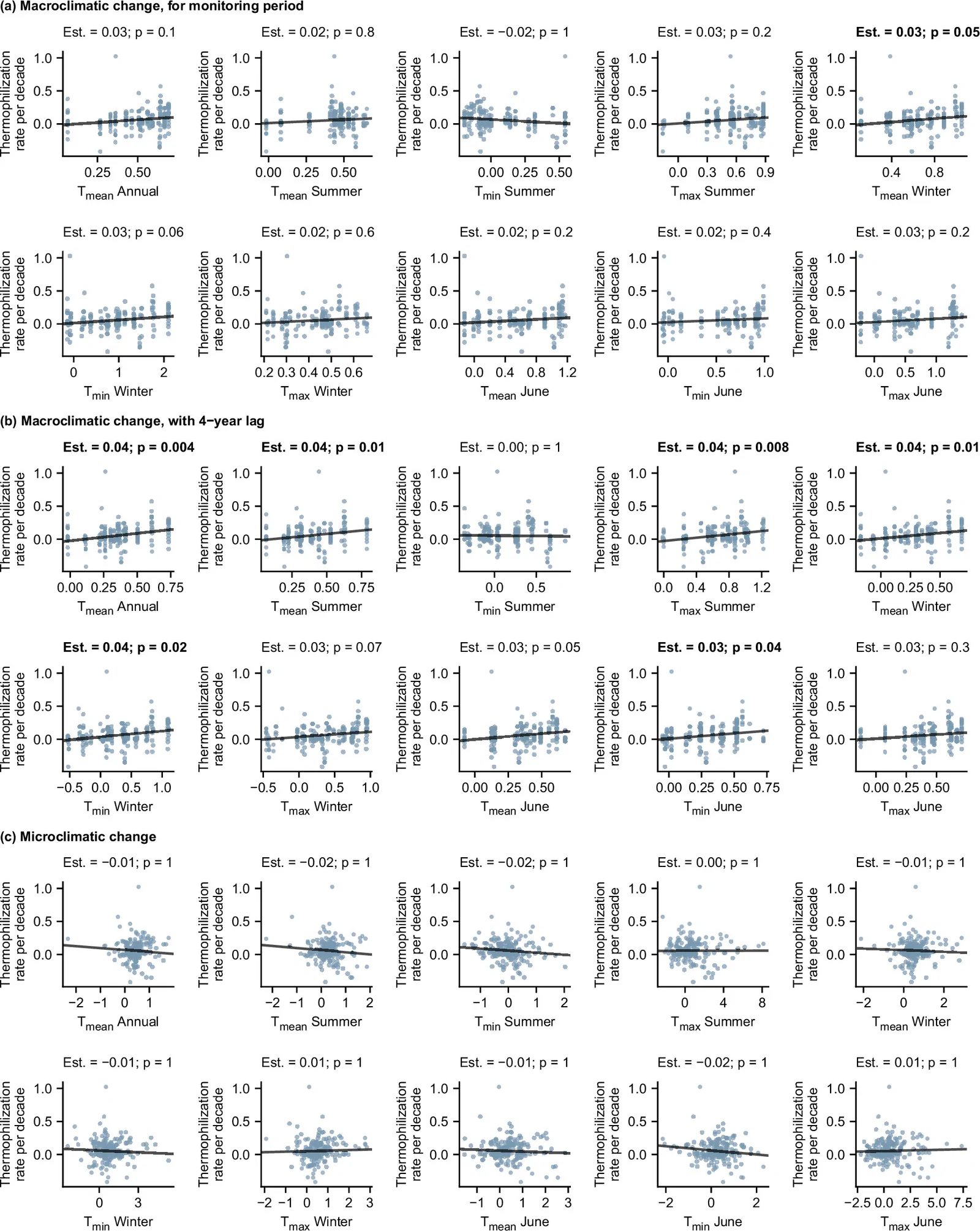
Relationships between thermophilization rates and rates of temperature change on the plot-cluster level. Relationships between thermophilization rate aggregated to the plot-cluster level via averaging and the rates of change in ten (**a-b**) summit-level macroclimatic (calculated for [**a**] the monitoring period or [**b**] with a 4-year time lag) and (**c**) plot-cluster-level microclimatic temperature metrics across the 192 plot clusters. Estimated effect (Est.) and p value of the respective temperature metric, derived from linear mixed-effect models, are provided above each panel. Only the one-sided, right-tailed p values adjusted for multiple comparisons are provided (this adjustment can lead to p values of exactly 1). Bold text indicates statistically significant p values. The models included a random-intercept term for summit nested in mountain region. The line represents the regression line of the fitted models. The estimated effects are reported based on models fitted with predictors scaled to zero mean and unit variance, while the unscaled data is depicted for better interpretability. Est. … estimated effect; T_mean_ … mean temperature; T_min_ … minimum temperature, T_max_ … maximum temperature.

**Extended Data Fig. 6:**
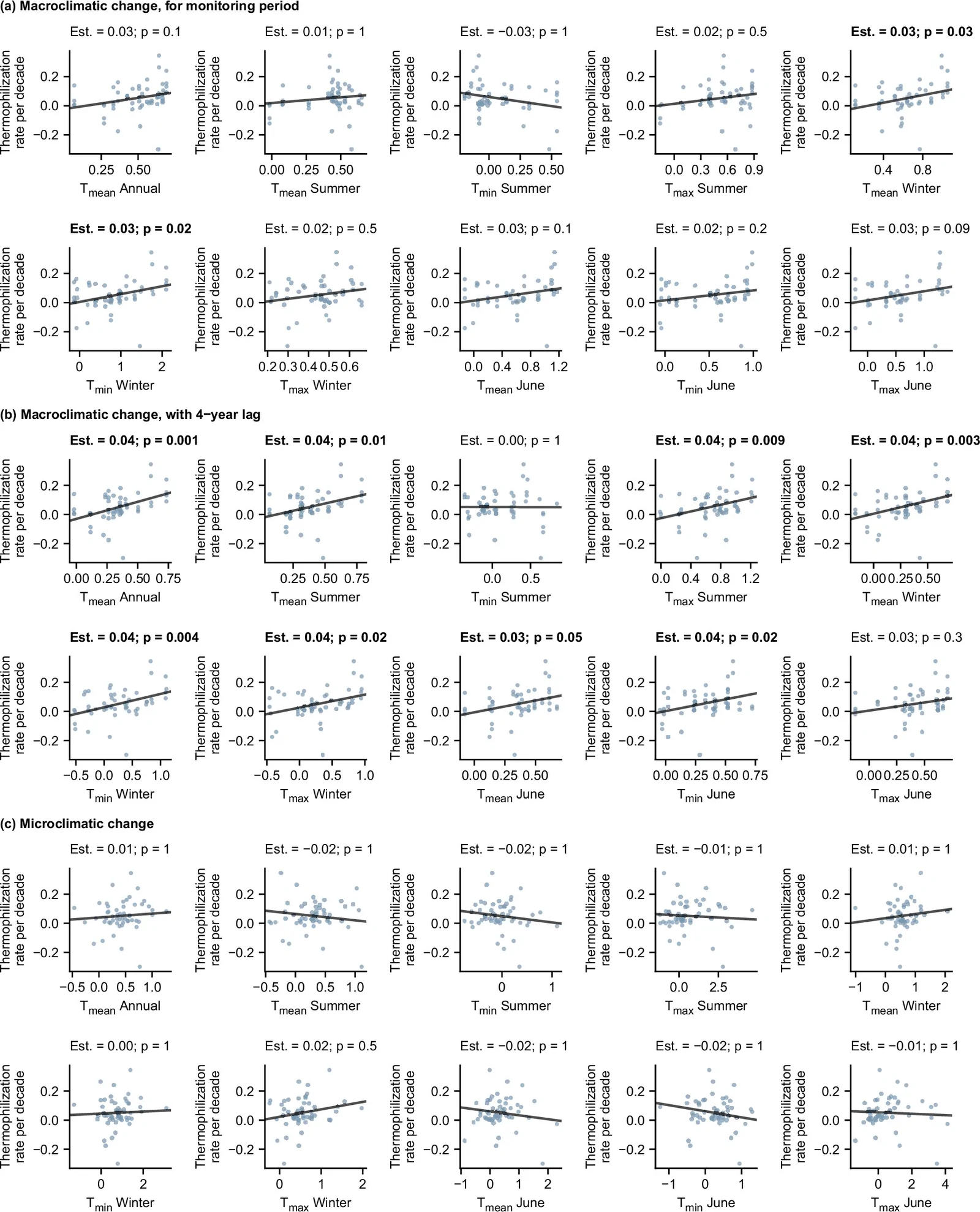
Relationships between thermophilization rates and rates of temperature change on the summit level. Relationships between thermophilization rate aggregated to the summit level via averaging and the rates of change in ten (**a-b**) summit-level macroclimatic (calculated for [**a**] the monitoring period or [**b**] with a 4-year time lag) and (**c**) microclimatic temperature metrics across the 53 summits. Estimated effect (Est.) and p value of the respective temperature metric, derived from linear mixed-effect models, are provided above each panel. Only the one-sided, right-tailed p values adjusted for multiple comparisons are provided (this adjustment can lead to p values of exactly 1). Bold text indicates statistically significant p values. The models included a random-intercept term for mountain region. The line represents the regression line of the fitted models. The estimated effects are reported based on models fitted with predictors scaled to zero mean and unit variance, while the unscaled data is depicted for better interpretability. Est. … estimated effect; T_mean_ … mean temperature; T_min_ … minimum temperature, T_max_ … maximum temperature.

**Extended Data Fig. 7:**
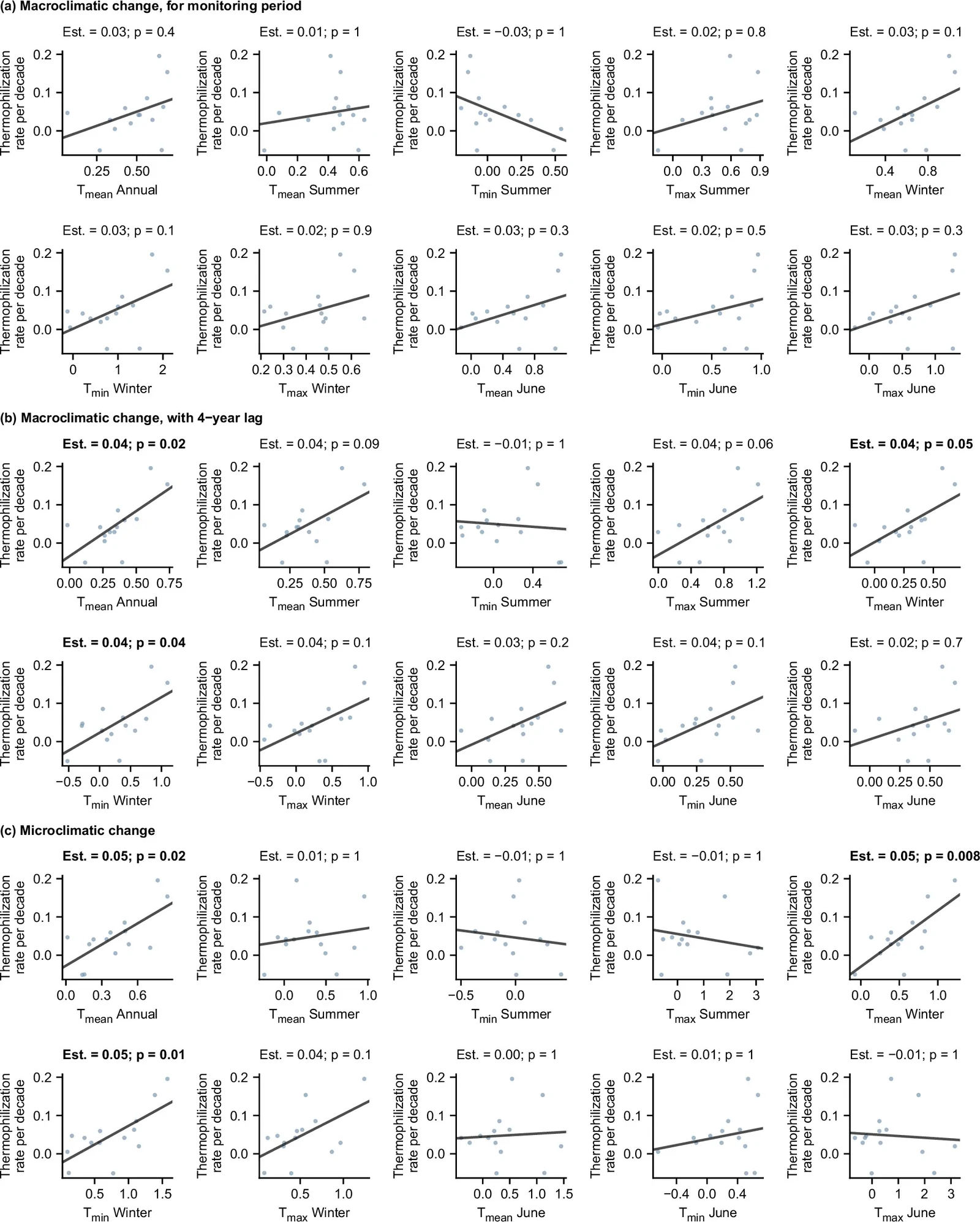
Relationships between thermophilization rates and rates of temperature change on the mountain-region level. Relationships between thermophilization rate aggregated to the mountain-region level via averaging and the rates of change in ten (**a-b**) mountain-region-level macroclimatic (calculated for [**a**] the monitoring period or [**b**] with a 4-year time lag) and (**c**) microclimatic temperature metrics across the 53 summits. Estimated effect (Est.) and p value of the respective temperature metric, derived from ordinary least squares (OLS) regressions, are provided above each panel. Only the one-sided, right-tailed p values adjusted for multiple comparisons are provided (this adjustment can lead to p values of exactly 1). Bold text indicates statistically significant p values. The line represents the regression line of the fitted models. The estimated effects are reported based on models fitted with predictors scaled to zero mean and unit variance, while the unscaled data is depicted for better interpretability. Est. … estimated effect; T_mean_ … mean temperature; T_min_ … minimum temperature, T_max_ … maximum temperature.

According to our hypotheses, the biotic and abiotic context can obscure the impact of warming on thermophilization. To explore this idea, we first abstracted from this plot-level context by aggregating thermophilization rates and rates of change in the temperature metrics on the plot-cluster, summit and regional scales. We found that the coarsest (regional) scale the relationship between trends of warming and the thermophilization rates became clearer in some of the temperature metrics, especially in mean and minimum winter temperature, but also in mean annual temperature (Extended Data Figs. 4–7). At this coarsest spatial scale the (averaged) thermophilization rates were again more strongly related to the macroclimatic temperature changes with a 4-year time lag than to macroclimatic temperature changes during the monitoring period; however, of the eight macroclimatic temperature metrics with a 4-year time lag that were significantly related to thermophilization rates on the plot scale, only three remained significant at the regional scale (Fig. 4a). Surprisingly, at this regional scale, microclimatic temperature change was better related to the thermophilization rates than macroclimatic temperature change with a 4-year time lag (Fig. 4b). Although significant relationships emerged for the identical temperature metrics (annual mean temperature and mean and minimum winter temperature) at the two climatic scales, microclimatic metrics consistently explained a higher proportion of the variation (*R*^2^) in thermophilization rates (Fig. 4).

### The importance of covariables for explaining thermophilization rates

**Fig. 5:**
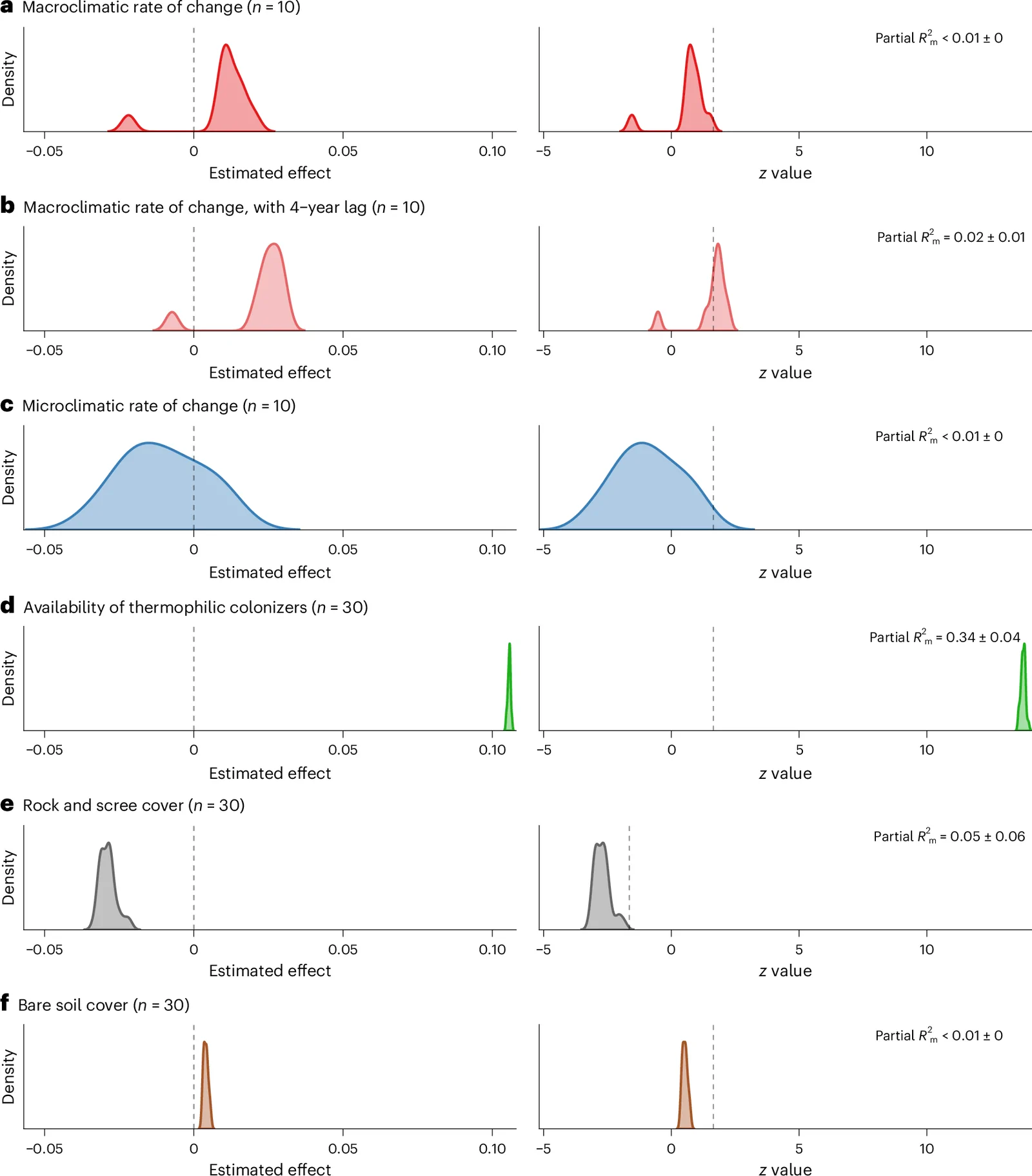
Results of linear mixed-effects models relating thermophilization rates to rates of temperature change and non-climatic covariables. **a**–**f**, Density distributions of the estimated effects (left) and *z* values (right) of the predictors as derived from the 30 linear mixed-effects models: 10 models each for rates of change in macroclimatic temperature metrics calculated for the monitoring period (**a**), or with a 4-year time lag (**b**) and microclimatic temperature metrics (**c**); compare Figs. 2 and 3, with thermophilization rates as response variable. For macroclimatic (**a**,**b**) and microclimatic rates of temperature change (**c**) these distributions incorporate the estimated effects and *z* values across all ten temperature metrics. Dashed lines indicate 0 for the estimated effects of the predictors (left) and the critical *z* values for one-sided tests (right; with the expectation of positive relationships for all but rock and scree cover, for which a negative relationship was tested). The number of models (*n*) that included the individual predictors were: *n* = 10 (**a**–**c**) and *n* = 30 (**d**–**f**). Partial *R*^2^_m_ values (mean ± s.d.) were calculated as the difference between the *R*^2^_m_ of the full models containing all predictors and the *R*^2^_m_ of a reduced model dropping the respective predictor. The mean ± s.d. *R*^2^_m_ value across the 30 full models was 0.37 ± 0.05.

Finally, the context-dependency of plot-level thermophilization rates was further explored. The single-predictor models of all macroclimatic (calculated over the monitoring period or with a 4-year time lag) and microclimatic temperature change metrics were combined with three covariables: availability of thermophilic colonizers in the surroundings of the plots, the proportion of rock and scree cover and the proportion of bare soil cover. We fitted each of these 30 multipredictor models twice, once accounting for all possible interactions between the rate of temperature change and each covariable (Supplementary Fig. 2) and once without accounting for any interactions, that is only considering additive effects of the predictors (Fig. 5). These models revealed no significant positive effect for any of the microclimatic or macroclimatic temperature metrics as long as the latter were calculated for the monitoring time window. However, consistent with the previous results, most macroclimatic change metrics remained significant in these models when calculated for a time window preceding the monitoring interval by four years with partial *R*^2^_m_ values slightly lower than in the single-predictor models (Fig. 5a–c).

**Fig. 6:**
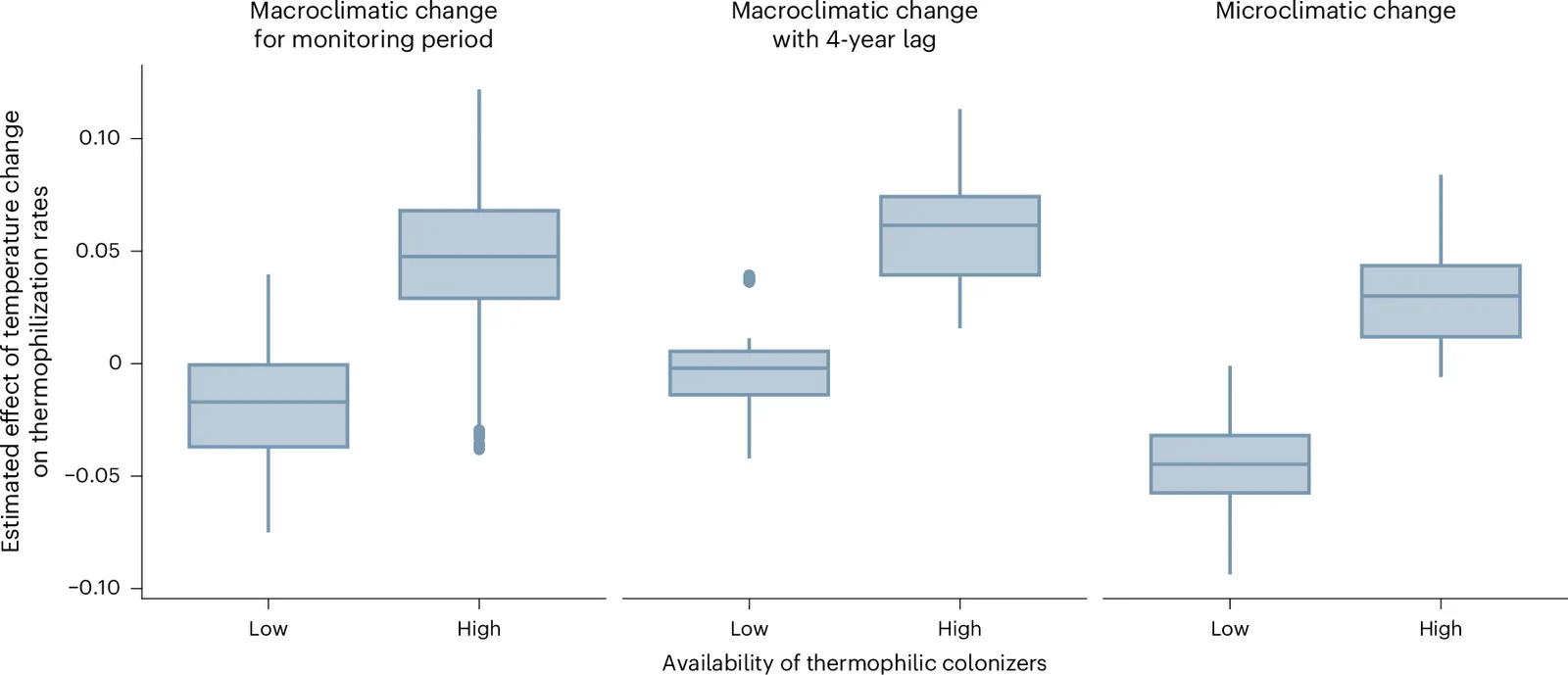
Interactive effects of temperature change and the availability of thermophilic colonizers on thermophilization rates. The estimated effects of a one-unit increase in the rate of temperature change on thermophilization rates dependent on the availability of thermophilic colonizers in the surroundings of the plots, derived from the 30 multipredictor linear mixed-effects models that accounted for interactions between rates of temperature change across all 10 temperature metrics and the covariables. The estimated effects are depicted separately for models including macroclimatic temperature change calculated for the monitoring window, macroclimatic change calculated with a 4-year time lag and microclimatic change. The estimated effects were calculated by adding the main effect of the rate of temperature change on thermophilization rates to the effect of the interaction between the rate of temperature change and the availability of thermophilic colonizers, separately for each model. The effect of the interaction was calculated for the lower (low) and upper (high) 5% quantiles of availability of thermophilic colonizers values in our data (*n* = 360 per box; 10 temperature metrics × 36 quantiles values). Boxes indicate the median and interquartile range (IQR; 25th and 75th percentiles), whiskers extend to the furthest data points within 1.5× IQR from the hinges and points represent outliers.

However, overall *R*^2^_m_ values of these models greatly increased as a result of the inclusion of the three covariables as predictors, with a mean *R*^2^_m_ of 0.37 across all models. Both the availability of thermophilic colonizers in the surroundings of the plots (very strong positive effect: mean partial *R*^2^_m_ = 0.34; Fig. 5d) and the proportion of rock and scree cover (moderate negative effect: mean partial *R*^2^_m_ = 0.05; Fig. 5e) were significant predictors of thermophilization rates across all 30 models. The proportion of bare soil cover had no significant effect on thermophilization rates (Fig. 5f). Significant interactions emerged between temperature change and the availability of thermophilic colonizers in the surroundings of the plots: in general, the impact of temperature change on thermophilization rates became stronger with an increasing availability of thermophilic colonizers (Fig. 6).

## Discussion

Our results demonstrate a significant thermophilization signal across the summit communities of Europe within 21 years, both on average across all plots and in nearly two-thirds of the individual plant communities. This is in line with trends reported by other studies conducted over shorter time spans^6,11,38^ and highlights that thermophilization has been an ongoing process for at least two decades on European summits.

Despite this very widespread thermophilization signal, the intuitive relationship between thermophilization rates and either the rate of macroclimatic or microclimatic warming was surprisingly weak, at least at the scale of individual plant communities. In particular, temperature change hardly contributed to the observed thermophilization signal when both rates were calculated for the same time window and only a single temperature metric was considered as explanatory variable. Relationships became clearer when time lags between temperature change and vegetation responses were considered and when temperature change was captured in a more complex way, that is by two different, potentially interacting metrics. However, even the best-performing models explained less than 10% of the observed variation in thermophilization rates.

These findings demonstrate that the intuitive causal link between a warming climate and plant communities becoming increasingly dominated by more warmth-associated species is surprisingly difficult to substantiate even with the most extensive empirical dataset currently available for alpine vegetation. Although thermophilization rates were significantly related to metrics of macroclimatic temperature change, the detection of this relationship was highly sensitive to the, often subjective, selection of temperature metrics and the search for an appropriate lag time between temperature and vegetation change, which is often neglected^11,18^ (Fig. 3 and Extended Data Fig. 3). In fact, such time lags can be long in stress-tolerant alpine vegetation, especially if the vegetation response considered depends on local colonizations and extinctions^24,39,40^. The time lag of 4 years found to deliver the best models in our analyses is relatively short, probably because the thermophilization metric considered depends on cover changes of resident species at least as much as on species turnover^6^. As these communities are mostly dominated by perennial rather than annual species in the seasonal climate of European summits, cover shifts are probably much more responsive to short-term climatic trends than colonization and extinction dynamics. Furthermore, our results demonstrate that a more complex description of temperature change, by accounting for several temperature metrics and their interactions, improves the attribution of observed vegetation changes to the underlying climatic drivers. Such complex models involving interaction terms are rarely used in similar analyses (compare, for example, refs. ^6,7,38^). The superior fit of these more complex models suggests that different facets of temperature change, such as varying seasonal rates of change, have different impact in different regions; for example due to varying sensitivity to winter temperature change dependent on the length and reliability of snow cover^41,42^ (more detailed discussion below).

Against our expectations, the climatic effect on thermophilization was even less detectable when on-site measurements of soil temperature were considered instead of interpolated macroclimatic temperatures, at least at the spatial resolution of individual plots and summits. We assume that this weak relationship, but also the higher explanatory power of microclimate at the coarsest (regional) scale is due to the overwhelming effect of the biotic and abiotic context within which individual communities grow and change. We suppose that at the coarse regional scale, microclimatic measurements more precisely represent the warming trend to which the average community in a region is exposed. As a consequence, the relationship with the thermophilization rate of this average community is stronger than it would be when computation is based on interpolated macroclimatic trends. At the fine scale of individual plots, however, the strong variation in biotic and abiotic environments overrides the more subtle impact of how warming varies at different sites within one summit. Smoothing across these differences by using macroclimatic metrics, which do not vary across the plots within one summit, makes climate warming again visible as the ultimate driver of thermophilization.

On the level of individual plots, vegetation dynamics are thus obviously idiosyncratic, with many local communities even changing towards less contribution of warmth-associated species, and a complete loss of any relationship between microclimatic warming and thermophilization. According to our results, a factor of particular importance is the availability of species that can potentially contribute to thermophilization in the immediate surroundings of a community, emphasizing the strong impact that dispersal limitation has on alpine vegetation dynamics (for example, refs. ^32,43^). The effect of the surrounding source pool probably has both a random and a deterministic component. With a stronger difference in the climatic requirements of species in a plot and its surroundings, thermophilization rates will be higher just by chance alone, because of a higher likelihood that random colonizations by species from the surroundings involve more warmth-associated species. In addition, a deterministic, warming-driven change towards a more warmth-associated community will also happen faster if nearby populations of warmth-associated species provide the necessary propagule input. We cannot quantify the relative contribution of random and deterministic dynamics to the strength of the effect detected here. However, we emphasize that the sampling design of GLORIA, with plot clusters oriented in the four cardinal directions of a summit, is well suited to represent differences in microclimate among the plots on a summit^44^. In many cases, plots will thus show a ‘colder’ species composition than their surroundings, not by chance alone, but because they are actually situated in microsites colder than the average surroundings, for example on north-facing slopes. As a corollary, the design is highly sensitive to the detection of a deterministic influx of new species into the plots driven by alleviation of local thermic constraints. Moreover, the pronounced interaction between rate of warming and the local availability of thermophilic colonizers also suggests that the strong impact of our proxy variable is probably driven by deterministic rather than random change to a considerable extent.

Apart from the availability of thermophilic colonizers in the surroundings of the plots, a higher proportion of rock and scree substrates hinders thermophilization, probably because it constrains stronger abundance changes, limits the pool of new colonizers and provides microrefugia for cold-associated species. Indeed, the peculiar properties of scree and rock habitats are a well-established filter which only a small subset of alpine plants can pass (for example, ref. ^33^). The effect of this variable on thermophilization rates was much weaker than the one of available thermophilic colonizers, but still stronger than most temperature change metrics. The proportion of bare soil, the other variable describing substrate conditions, was significantly positively related to thermophilization, but only in a few of the models that considered interactions with temperature change and with negligible explanatory power. A potential reason may be the ambiguity of this covariable in terms of vegetation dynamics. On the one hand, the availability of bare soil may indicate rough climatic conditions (and hence reduced colonization options). But on the other hand, it could also reflect a colonization opportunity due to suitable substrate with no or reduced resistance from the resident community^45^.

Apart from these variables, there may be several other factors at play that we could not consider here. Among them, the two perhaps most important ones are changes in snow cover and water availability. Snow cover duration is a major determinant of alpine species distributions, and its change may have an impact on community turnover that exceeds the one of temperature change^42^. With respect to thermophilization, a generally warmer climate may reduce snow cover duration and hence expose species to cooler minimum temperatures in spring^46,47^. The negative trend in minimum summer temperatures in our data, in contrast to all other metrics (Fig. 2), may result from shorter snow cover duration, at least as far as microclimatic temperatures are concerned. Depending on the strength and direction of this complex interaction between snow cover and temperature change, the strength of the thermophilization signal might thus become decoupled from temperature trends. In addition, snow cover in spring and precipitation during the growing season codetermine water availability in alpine vegetation^48^. Although limited water availability does not (yet) seem to constrain plant distribution in the ‘average’ alpine vegetation of temperate and boreal mountains, it may have already been relevant for recent vegetation changes in Mediterranean alpine habitats^49,50^. More generally, alpine plants adapted to humid temperate conditions may increasingly suffer from unusually dry summers, as suggested by rain-out shelter experiments, which affected alpine species more severely compared with their low-elevation congeners^51^. As a corollary, increasing drought stress might be an additional constraint on thermophilization dynamics and its impact may vary strongly across the mountain ranges included here and perhaps also locally among plots dependent on soil properties and topography^30,52^.

In summary, our results demonstrate a significant thermophilization signal in the plant communities of European summits but only weak relationships with climate warming at the scale of local communities. While warming and thermophilization correlate relatively well when averaging trends across mountain regions, the response of individual plant communities is far more complex and context-dependent than many predictive models of alpine vegetation change assume. In particular, the strong effect of the surrounding species pool in our data emphasizes the importance of dispersal limitation for how plant communities change with climate, in line with previous findings^24,39^. Other factors that we could not consider, such as altered snow cover or water availability, and possibly also other facets of anthropogenic environmental change such as nitrogen deposition^8^, probably add to this complexity. We conclude that long-term monitoring datasets such as GLORIA are invaluable for documenting alpine vegetation change. However, to improve our understanding of this change, they should be expanded towards a more complete sampling of the various, potentially interacting drivers and constraints.

## Methods

### Study area

We analysed data from 14 mountain regions across Europe (Fig. 1), each comprising three to four summits (*n* = 53), covering an elevational gradient from around the treeline up to either the limits of vascular plant life or the uppermost available vegetation zone. On each summit, 16 vegetation plots of 1 m × 1 m were permanently marked, four in each cardinal direction, forming so-called ‘plot clusters’ (Supplementary Fig. 1). On a few summits, less than 16 vegetation plots were marked because of topological constraints. In the centre of each plot cluster, soil temperature was measured at hourly intervals at 10-cm soil depth using StowAway TidbiT (until 2008; accuracy ±0.4 °C) and GEOprecision M-Log 5 W (from 2008 onward; accuracy ±0.2 °C) data loggers, covering time spans ranging from 2 years to 21 years (Supplementary Table 1). Some vegetation plots were excluded from the analyses, reducing the total number of vegetation plots analysed to 724, because they were not monitored as a result of inaccessibility (*n* = 14), contained no vascular plants in at least two surveys (*n* = 80) or lacked sufficiently long time series (≥10 years) of soil temperatures as a result of data logger malfunctions (*n* = 30).

Vegetation plots were surveyed four times between 2001 (see Supplementary Table 1 for exceptions) and 2022, using the standardized GLORIA Multi-Summit approach^37^ (global observation research initiative in alpine environments). During each survey, the cover of each vascular plant taxon (determined to the species or subspecies level) was visually estimated (in percentage, down to 0.005%). Additionally, all taxa occurring in the uppermost 10 m below the summit were recorded and their abundances estimated according to five ordinal abundance categories (very rare, rare, scattered, common and dominant).

The names of recorded taxa were standardized using the WorldFlora package^53^ (version 1.14-5; taxonomic backbone version v.2022.12) in R (version 4.5.0)^54^. For taxa that could not be resolved this way, we used the Plants of the World Online (powo.science.kew.org) database for taxonomic standardization. We removed cases where a taxon recorded in one survey was not redetected in the subsequent survey but was replaced by a different taxon of the same genus. Because such potential misidentifications across surveys may bias estimates of species turnover over time we excluded the affected taxa from all surveys of the corresponding plot. We further removed some taxonomically complex genera (*Alchemilla*, *Euphrasia*, *Hieracium*, *Pilosella* and *Taraxacum*) because of potential misidentification issues or ambiguous taxonomic concepts.

### Thermophilization rates

We quantified the rate of thermophilization across the four surveys for each vegetation plot. Each taxon occurring in any of the plots in any survey was assigned an elevational rank (sensu ref. ^6^; herein referred to as ‘altitudinal rank’; Supplementary Table 2), based on the vertical distribution ranges of taxa as indicated in regional standard floras and expert judgments to harmonize different schemes and indications. The elevational rank reflects the elevational distribution optimum of a taxon (along a gradient from the nival (1) to the colline (7) elevational vegetation belts; Supplementary Table 2). Whereas the elevational ranks in ref. ^6^ range from 1 (nival) to 6 (montane) we reassessed taxa along a gradient also including the colline vegetation belt (coded as 7). For each plot in each survey, we calculated the cover-weighted mean elevational rank across all co-occurring taxa recorded separately for each survey. We then, separately for each plot, regressed the cover-weighted mean elevational ranks against the years (as continuous predictor) during which the surveys were conducted, using ordinary least squares (OLS) regression, and extracted the slope coefficient estimates. These estimates represent the change in the cover-weighted mean elevational rank per year which we then converted to change per decade as a metric of plot-level thermophilization rate, with positive values indicating an increase of the share of taxa with distribution centres located at lower elevations.

We consider elevational ranks adequate proxies for the temperature niche optima of taxa relative to each other. Although other factors certainly influence elevational distributions, we argue that temperature is the main driver and similar issues exist in other metrics (for example, ecological indicator values or niche optima derived from species distribution models). The elevational ranks are further available for all taxa occurring in the analysed vegetation plots, which was not the case for other metrics. However, to ensure the robustness of our results, we repeated the analyses using ecological indicator values for temperature (hereafter referred to as EIV T), indicating the relative temperature niche positions of the taxa on a scale ranging from 0 (cold) to 10 (warm), as calculated by refs. ^55,56^ which were available for 93% of the taxa (620 of 668). For six vegetation plots the cover-weighted mean EIV T could not be calculated because EIV T were not available for any of the taxa in these plots. These analyses therefore only considered 718 vegetation plots. As cover-weighted means are potentially largely determined by the most dominant taxa, therefore masking changes triggered by colonizing taxa where individuals or local populations have just started to grow^57^, we repeated the analyses using the unweighted means of both elevational ranks and EIV T. The results of these analyses are provided in Supplementary Information, ‘Results of analyses with three additional types of thermophilization rates’. All these additional analyses delivered results that were qualitatively similar to those reported in the main text of the paper.

### Rates of temperature change

We quantified the rate of temperature change across the four surveys, based on both macroclimatic data (time series from interpolated grids of weather station data) and microclimatic data (time series from soil temperature logger measurements). For macroclimate (ambient air temperature), we statistically downscaled monthly mean, minimum and maximum temperatures to a 100-m resolution over the whole time period covered by the surveys by relying on data from the Climate Research Unit^58^ (CRU: CRUTS v4.09). To downscale the temperatures, we used the methodology described in ref. ^39^, comprising the calculation of lapse rates and spatial interpolation using QGIS Desktop^59^ and R^54^. For microclimate (soil temperature), we computed the same summary statistics (monthly means, minima and maxima) but from the soil temperature logger data. The logger data were first cleaned by removing obviously erroneous temperature values, for example measurements taken before deployment and after retrieval (when the logger was not in the soil) or when temperatures showed abrupt, sustained shifts or increased variability, indicative of loss of soil insulation (logger surfacing). Across all loggers, an average of 1.1% (s.d. = 2.9%, minimum 0%, maximum 25.5%) temperature values were removed. We first aggregated the hourly temperature measurements to daily statistics, by calculating daily means, minima and maxima. Days that were lacking more than 10% of hourly values were discarded. The daily values were then further aggregated to monthly values by calculating monthly means, again discarding months lacking more than 10% of daily values.

From these monthly values, we calculated for each year and scale (macroclimatic and microclimatic) the following temperature metrics: mean annual temperature (the mean of the 12 monthly means), as well as mean, minimum and maximum temperature (*T*_mean_, *T*_min_ and *T*_max_) in summer (May to September), winter (November to March of the same year, effectively pooling data from two winters) and June. These metrics were chosen because mean annual temperature integrates over several temperature variables probably affecting plant survival, summer temperatures affect plant growth and reproduction during the growing season and winter temperatures are important determinants for plant survival during winter dormancy. The specific time frame for calculating summer temperature metrics was selected to ensure that it comprised the peak growing seasons across all studied mountain regions. We extracted temperature metrics for the single month of June as they have previously been shown to correlate with thermophilization in mountain plant communities^6^. If any of the required monthly values were not available, the respective metric was not calculated for that year. This resulted in a total of ten temperature metrics for each of the two climatic scales.

Separately for each of the 53 summits (for macroclimate) and each of the 192 plot clusters (for microclimate), as well as for each of the 10 temperature metrics, we fitted OLS regressions relating temperature values to time (that is, the respective years) so as to extract the slope coefficient estimate reflecting the rate of change in °C yr^−1^, which we converted to °C per decade. For the microclimatic temperature metrics these regressions were only fitted if data were available for at least four different years and covering a time span of at least 10 years. Note that soil temperature loggers are associated with plot clusters and not individual vegetation plots as they are placed at the centres of the plot clusters (four loggers per summit). Therefore, all four plots of a plot cluster are associated with the same rate of microclimatic temperature change in our analyses. Furthermore, all plots of a summit have the same rate of macroclimatic temperature change due to the 100-m resolution of the downscaled macroclimatic data. Correlations between the macroclimatic and microclimatic representations of the same temperature metrics were evaluated using Pearson correlation coefficients.

To assess potentially delayed responses of plant communities to changing temperatures, we calculated ten additional temporal variants for each macroclimatic rate of temperature change metric. Using a 1-year sliding time window, we iteratively shifted the time window across which temperatures were related to time into the past in 1-year increments for up to 10 years. Instead of describing macroclimatic rates of temperature change over the monitoring period (2001–2022), these variants describe the rates of temperature change in prior years (2000–2021, 1999–2020 and so on until 1991–2012). All analyses were repeated using these temporal variants and the results of the best-performing temporal variant are presented in the main text alongside the results of the monitoring-period variant.

### Environmental variables and availability of thermophilic colonizers

We considered the fraction of a given vegetation plot covered by either rock or scree (rock and scree cover) in the first survey to quantify substrate constraints on colonization. Additionally, the fraction of a plot not covered by either vegetation or rock and scree (that is, bare soil cover) in the first survey was calculated to express the ‘competition-free’ space available for colonization by plants, assuming that these would be favourable germination niches.

Finally, we calculated a variable to quantify the availability of thermophilic colonizers in the surroundings of the monitored plots, using only the data from the first survey. We computed the difference between the cover-weighted mean elevational rank of the taxon pool of the summit (all plant taxa recorded in the uppermost 10 elevational metres) and the cover-weighted mean elevational rank of each plot on the respective summit. As percentage covers were not estimated for the taxa in the uppermost 10 m of the summits, we translated the ordinal abundance categories into percentage values (very rare 0.5%; rare 3%; scattered 15%; common 37.5%; dominant 75%). This variable hence quantifies how likely thermophilization of the focal plot is to occur via neutral processes, that is the probability that a new taxon colonizing from the immediate neighbourhood will increase the average elevational rank of the plot. Elevational ranks were not available for four taxa with a total of four occurrences across all summits, never accounting for more than 3% of the taxon pool of a summit. These taxa were therefore not considered for the calculation of this variable. Moreover, this variable could not be calculated for four plots which did not contain any plant taxa in the first survey. Three additional versions of this variable were calculated for the analyses concerning the rates of change in cover-weighted mean EIV T and the unweighted means of elevational rank and EIV T. Note that EIV T values were not available for 55 taxa with a total of 138 occurrences accounting for up to 67% of the taxon pool of a summit (mean 6%).

### Statistical analyses

All analyses were conducted using data of the 724 vegetation plots distributed across the 14 mountain regions and the 53 summits for which all necessary data were available. We initially tested whether the mean thermophilization rate across all plots was significantly greater than zero by fitting an intercept-only linear mixed-effects model to the plot-level thermophilization rates with a random-intercept term for plot cluster nested in summit nested in mountain region to account for the hierarchical structure of the dataset. We further tested whether the mean rates of temperature change per decade were significantly greater than zero for the ten temperature metrics at both scales. For this purpose, we again fitted intercept-only linear mixed-effects models to the plot-cluster rates of temperature change and the summit-level rates of temperature change for the microclimatic and macroclimatic metrics, respectively, with a random-intercept term for summit nested in mountain region for the former and only mountain region for the latter. As we were interested in whether both thermophilization rates and rates of temperature change were significantly greater than zero we calculated one-sided *P* values from the resulting *z* values of the models. Performing multiple hypothesis tests on the same dataset can increase the chance of false positives, therefore we further adjusted these *P* values using the Bonferroni method^60^ separately for the intercept-only models of the macroclimatic and microclimatic metrics (ten each).

We then analysed whether the plot-level thermophilization rates could be explained by either macroclimatic or microclimatic trends by fitting linear mixed-effects models with a random-intercept term for plot cluster nested in summit nested in mountain region to account for both the hierarchical structure of the dataset and pseudoreplication of the predictor variables that is already present at the plot-cluster level (that is, all plots in a given plot cluster have the same rate of microclimatic temperature changes and all plots on a given summit have the same rate of macroclimatic temperature change). The thermophilization rates were used as the response variable and any one of the macroclimatic or microclimatic temperature variables acted as the single fixed-effects predictor variable, for a total of 20 such models. To further consider additive and interactive effects of pairs of temperature variables on thermophilization rates, we also fitted linear mixed-effects models that included two temperature variables and their interaction term as fixed effects with the random effect structure described above. We considered interactions only between either macroclimatic or microclimatic variables, but not between one macroclimatic and one microclimatic variable, and only between variables that were not highly correlated, that is with an absolute Pearson correlation coefficient less than or equal to 0.5. Before fitting the models, we scaled all predictor variables to zero mean and unit variance. We specifically tested whether thermophilization rates were positively related to rates of temperature change and therefore calculated one-sided *P* values from the *z* values of the models. To correct for performing multiple hypothesis tests on the same dataset, we further adjusted these *P* values using a variation of the Bonferroni method^60^, separately for the models including either macroclimatic or microclimatic temperature variables. Because the Bonferroni method becomes overly conservative if the predictor variables of the different models are correlated, we did not simply multiply the *P* values by the number of models fitted but rather by 1 + (*n* − 1) × (1 − *c*), where *n* is the number of models fitted and *c* is the average of the absolute Pearson correlation coefficient between an individual predictor and all nine other predictors in the compared models. This correction factor is equal to the number of models fitted for a predictor that is not correlated to any of the other predictors (correlation coefficient of zero) and therefore equal to the original Bonferroni method in case of no correlation. However, in the case of correlated predictors the *P* values are adjusted to a lesser degree. The mixed-effects models were fitted using the glmmTMB package (version 1.1.14)^61^ and their marginal *R*^2^ values^62^ (*R*^2^_m_) were calculated using the performance package (version 0.17.0)^63^ in R (version 4.5.0)^54^.

To analyse whether the relationship between thermophilization rates and rates of temperature change depended on the spatial scale, we aggregated (via averaging) the thermophilization rates and the rates of temperature change to the plot-cluster, summit and regional scale. We then repeated the approach described above, while adjusting the random-intercept term to summit nested in mountain region for the models fitted to the plot-cluster-level data, and to only mountain region for those fitted to the summit-level data. An OLS regression (that is, without a random effect structure) was fitted to the region-level data.

We then fitted the same models again but added as additional covariables in the fixed effects: (1) the availability of thermophilic colonizers in the surroundings of the plots; (2) the proportion of rock and scree cover within each plot; and (3) the proportion of bare soil cover within each plot. Each of these models was fitted twice, once accounting for interactions between the respective temperature change variables and each covariable without considering interactions among the covariables themselves, and once without accounting for interactions, that is only considering additive effects of the predictors. Correlations between all predictors were checked, but correlations greater than 0.7 or less than −0.7 did not occur between any of the variables used within a single model (Supplementary Fig. 3). We further checked the models for multicollinearity by calculating the variance inflation factor (VIF) using the performance package^63^ but found no multicollinearity in the models (mean VIF = 1.05, minimum 1.00, maximum 1.29). From these models we then extracted the estimated effects and *z* values of all predictors. For each of these 20 models we also fitted four reduced models by individually dropping one of the predictors. We then calculated the difference in the *R*^2^_m_ of the full and the reduced models to quantify the explanatory power of the dropped predictors (partial *R*^2^_m_). As the availability of thermophilic colonizers could not be calculated for four plots, these models were fitted to the data of only 720 plots. For an overview of all predictor variables used across any of the fitted models see Supplementary Table 3.

### Reporting summary

Further information on research design is available in the Nature Portfolio Reporting Summary linked to this article.

## Supplementary information

Supplementary Information Supplementary Figs. 1–5, Tables 1–13 and analyses (geographical and elevational trends; repeated analyses using three additional types of our primary response variable (thermophilization rate) presented/analysed in the main text).

Reporting Summary

Peer Review File

## Data Availability

The data that supports the findings of this study are available via Figshare at https://doi.org/10.6084/m9.figshare.32665953 (ref. ^64^).

## Code availability

The code for replicating the presented analyses and figures is available via Figshare at https://doi.org/10.6084/m9.figshare.32665953 (ref. ^64^).
