# Publisher Correction: Widespread thermophilization but weak link to climate warming in Europe’s summit plant communities

**DOI:** 10.1038/s41559-026-03189-w

**Published:** 2026-09-02

**Authors:** Johannes Hausharter, Johannes Wessely, Norbert Helm, Kryštof Chytrý, Karl Hülber, Dietmar Moser, Mary Carolina García Lino, Katharina Kagerl, Andrea Lamprecht, Jonathan Lenoir, Andreas Kollert, Andreas Mayr, Martin Rutzinger, Philippe Choler, Pieter De Frenne, Jonas J. Lembrechts, Otar Abdaladze, Chris Andrews, Peter Barančok, José-Luis Benito-Alonso, Eva M. Cañadas, Michele Carbognani, Pau Carnicero, Emmanuel Corcket, Michele Di Musciano, Jan Dick, Khatuna Gigauri, Andreas Hilpold, Juan J. Jiménez, Juan Lorite, Umberto Morra di Cella, Panagiotis Nyktas, Simone Orsenigo, Andrej Palaj, Martina Petey, Mihai Pușcaș, Christophe Randin, Ilektra Remoundou, Christian Rixen, Patrick Saccone, Angela Stanisci, Jean-Paul Theurillat, Tudor-Mihai Ursu, Thomas Vanneste, Raphael S. von Büren, Sonja Wipf, Manuela Winkler, Harald Pauli, Stefan Dullinger

**Affiliations:** 1 Department of Botany and Biodiversity Research, University of Vienna, Vienna, Austria; 2 Vienna Doctoral School of Ecology and Evolution, University of Vienna, Vienna, Austria; 3 GLORIA co-ordination, Institute for Interdisciplinary Mountain Research, Austrian Academy of Sciences, Vienna, Austria; 4 GLORIA co-ordination, Department of Ecosystem Management, Climate and Biodiversity, BOKU University, Vienna, Austria; 5 UMR CNRS 7058 “Ecologie et Dynamique des Systèmes Anthropisés” (EDYSAN), Université de Picardie Jules Verne, Amiens, France; 6 Department of Geography, University of Innsbruck, Innsbruck, Austria; 7 Université Grenoble Alpes, Université Savoie Mont Blanc, CNRS, LECA, Grenoble, France; 8 Forest & Nature Lab, Ghent University, Gontrode, Belgium; 9 Ecology & Biodiversity, Utrecht University, Utrecht, the Netherlands; 10 Plants & Ecosystems, University of Antwerp, Antwerp, Belgium; 11 Institute of Ecology, School of Natural Sciences and Medicine, Ilia State University, Tbilisi, Georgia; 12 UK Centre for Ecology and Hydrology, Penicuik, UK; 13 Institute of Landscape Ecology, Slovak Academy of Sciences, Bratislava, Slovak Republic; 14 Jolube Botanical Consultant, Jaca, Spain; 15 Department of Botany, University of Granada, Granada, Spain; 16 Department of Chemistry, Life Sciences and Environmental Sustainability, University of Parma, Parma, Italy; 17 Department of Botany, University of Innsbruck, Innsbruck, Austria; 18 Department of Animal Biology, Plant Biology and Ecology, Autonomous University of Barcelona, Bellaterra, Spain; 19 IMBE, Aix Marseille Université, Avignon Université, CNRS, IRD, Marseille, France; 20 Department of Life, Health and Environmental Sciences, University of L’Aquila, L’Aquila, Italy; 21 Department of Biological, Geological and Environmental Sciences (BiGeA), Alma Mater Studiorum, University of Bologna, Bologna, Italy; 22 Georgian Institute of Public Affairs, Tbilisi, Georgia; 23 Institute for Alpine Environment, Eurac Research, Bolzano, Italy; 24 Pyrenean Institute of Ecology, IPE, CSIC, Jaca, Spain; 25 Interuniversity Institute for Earth System Research, University of Granada, Granada, Spain; 26 Environmental Protection Agency of Aosta Valley, Saint Christophe, Italy; 27 Faculty of Geo-Information Science and Earth Observation (ITC), University of Twente, Enschede, the Netherlands; 28 Natural History Museum of Crete, School of Sciences and Engineering, University of Crete, Heraklion, Greece; 29 Department of Earth and Environmental Sciences, University of Pavia, Pavia, Italy; 30 A. Borza Botanic Garden, Babeș-Bolyai University, Cluj-Napoca, Romania; 31 Faculty of Biology and Geology, Babeș-Bolyai University, Cluj-Napoca, Romania; 32 Emil G. Racoviță Institute, Babeș-Bolyai University, Cluj-Napoca, Romania; 33 Institute of Earth Surface Dynamics (IDYST), University of Lausanne, Lausanne, Switzerland; 34 Fondation du Jardin botanique Flore-Alpe, Champex-Lac, Switzerland; 35 Interdisciplinary Centre for Mountain Research (CIRM), University of Lausanne, Bramois, Switzerland; 36 Mediterranean Agronomic Institute of Chania, Chania, Greece; 37 WSL Institute for Snow and Avalanche Research SLF, Davos, Switzerland; 38 Climate Change, Extremes and Natural Hazards in Alpine Regions Research Center CERC, Davos, Switzerland; 39 Institute of Botany of the Czech Academy of Sciences, Průhonice, Czech Republic; 40 Department of Biosciences and Territory, University of Molise, Termoli, Italy; 41 Department of Plant Sciences, University of Geneva, Chambésy, Switzerland; 42 Institute of Biological Research Cluj, National Institute of Research and Development for Biological Sciences, Cluj-Napoca, Romania; 43 Meise Botanic Garden, Meise, Belgium; 44 Department of Research and Monitoring, Swiss National Park, Zernez, Switzerland; 45 Department of Environmental Sciences, Research Group Ecology, University of Basel, Basel, Switzerland

**Keywords:** Climate-change ecology, Plant ecology, Macroecology

Correction to: *Nature Ecology & Evolution* https://doi.org/10.1038/s41559-026-03150-x, published online 14 August 2026.

In the version of the article initially published, the panel headings were missing from Fig. 5 and have now been added back to the HTML and PDF versions of the article.

