# Supplementary material for: Widespread thermophilization but weak link to climate warming in Europe’s summit plant communities
Source: Nat Ecol Evol. 2026 Aug 14;10(10):1838–51. doi: 10.1038/s41559-026-03150-x (PMC13642035; doi:10.1038/s41559-026-03150-x)
Supplement: Supplementary file 1 [file 41559_2026_3150_MOESM1_ESM.pdf]

nature ecology & evolution

Supplementary information https://doi.org/10.1038/s41559-026-03150-x

# Widespread thermophilization but weak link to climate warming in Europe's summit plant communities

In the format provided by the authors and unedited

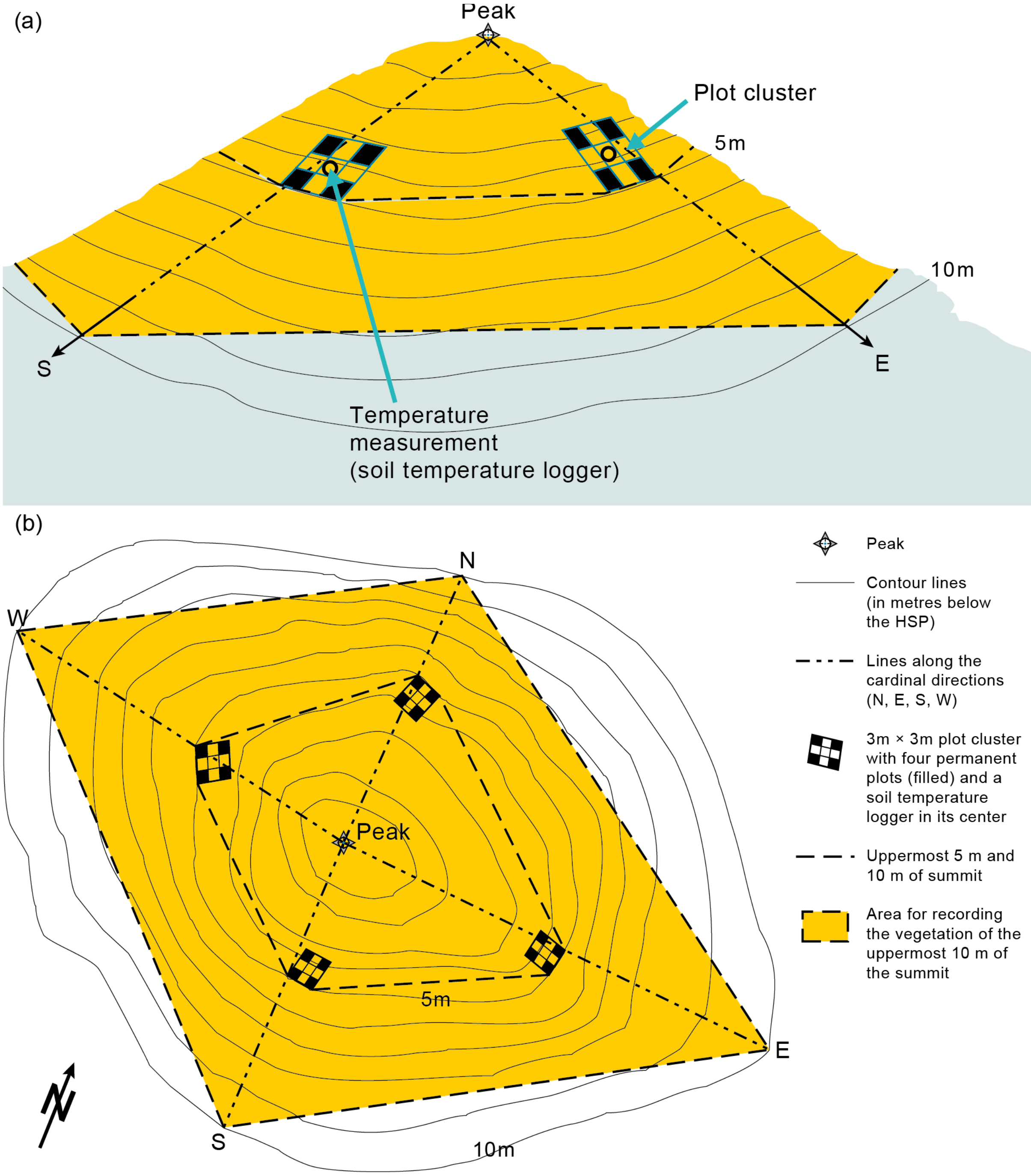


**Supplementary Fig. 1. Monitoring design employed in the GLORIA monitoring scheme.** Monitoring design for an exemplary summit in (a) side and (b) top view. A 3 m x 3 m plot cluster is located along each of the four cardinal directions (N, E, S, W) at the 5-m contour line. Each plot cluster is made up of four 1 m x 1 m permanent vegetation plots, and a soil temperature logger is buried in the ground in the plot cluster's center at 10 cm depth. The vegetation of the uppermost 10 m of a summit is recorded within the area shaded in yellow. Adapted from: Pauli, H. et al. The GLORIA Field Manual – Standard Multi-Summit Approach, Supplementary Methods and Extra Approaches. GLORIA-Coordination, Austrian Academy of Sciences & University of Natural Resources and Life Sciences, Vienna, 2015 (Fig. 3.1, p. 28).

**Supplementary Table 1.** Overview of the mountain regions and summits considered in this study, including the summits' elevation, latitude and longitude, the number of plots per summit included in this study, the years the four surveys were conducted in, the time span in years covered by the soil temperature measurements as well as the elevation of the current forestline. The country is represented by its 2-letter ISO code. While 16 plots were established on every summit, not all plots could be considered for this study. The number indicated in this table therefore represents the number of plots included in the analyses. In the case a survey on a summit was split between two years both years are provided. The time span covered by the soil temperature measurements differed between the temperature metrics (e.g. for calculating annual mean temperature all monthly means of a year had to be available, while only specific monthly means had to be available for calculating the other temperature metrics). In this table we provide the time span covered by the calculated annual mean temperatures, as this represents the minimum time span covered across all temperature metrics. As plots for which the soil temperature measurements covered less than 10 years were excluded from our analyses, the minimum time span indicated here is 10 years. The range of covered time spans is provided in cases where the four soil temperature loggers installed per summit differed in their covered time spans.

| Mountain region | Country | Summit code | Elevation (m) | Latitude | Longitude | Number of plots | Year 1st survey | Year 2nd survey | Year 3rd survey | Year 4th survey | Soil temp. (years) | Forestline (m) |
|---|---|---|---|---|---|---|---|---|---|---|---|---|
| S-Scandes / Dovrefjell | NO | VAR | 1,161 | 62.26 | 9.67 | 16 | 2001 | 2008 | 2015 | 2022 | 20 | 1,000 |
| S-Scandes / Dovrefjell | NO | VKO | 1,418 | 62.31 | 9.46 | 12 | 2001 | 2008 | 2015 | 2022 | 11–20 | 1,000 |
| S-Scandes / Dovrefjell | NO | KOL | 1,651 | 62.29 | 9.49 | 8 | 2001 | 2008 | 2015 | 2022 | 20 | 1,000 |
| S-Scandes / Dovrefjell | NO | SKI | 1,845 | 62.35 | 9.44 | 3 | 2001 | 2008 | 2015 | 2022 | 11–20 | 1,000 |
| Cairngorms | UK | MIG | 742 | 57.10 | -3.86 | 16 | 2002 | 2008 | 2015 | 2022 | 19–20 | 525 |
| Cairngorms | UK | CAM | 904 | 57.09 | -3.84 | 13 | 2001 | 2008 | 2015 | 2022 | 16–20 | 525 |
| Cairngorms | UK | UNK | 978 | 57.08 | -3.82 | 16 | 2001 | 2008 | 2015 | 2022 | 16–20 | 525 |
| Cairngorms | UK | SGO | 1,111 | 57.08 | -3.81 | 16 | 2001 | 2008 | 2015 | 2022 | 18–20 | 525 |
| W-Carpathians / High Tatra | SK | KRI | 1,919 | 49.18 | 19.95 | 16 | 2002 | 2008 | 2015 | 2022 | 13 | 1,550 |
| W-Carpathians / High Tatra | SK | VEK | 2,052 | 49.20 | 19.97 | 16 | 2002 | 2008 | 2015 | 2022 | 13–17 | 1,550 |
| W-Carpathians / High Tatra | SK | SED | 2,061 | 49.15 | 20.02 | 12 | 2001 | 2008 | 2015 | 2022 | 13 | 1,550 |
| W-Carpathians / High Tatra | SK | KRA | 2,336 | 49.16 | 20.01 | 16 | 2002 | 2008 | 2015 | 2022 | 13 | 1,550 |

**Supplementary Table 1.** Continued.

| Mountain region | Country | Summit | Elevation (m) | Latitude | Longitude | Number of plots | Year 1st survey | Year 2nd survey | Year 3rd survey | Year 4th survey | Soil temp. (years) | Forestline (m) |
|---|---|---|---|---|---|---|---|---|---|---|---|---|
| NE-Alps / Hochschwab | AT | ZIK | 1,910 | 47.60 | 15.09 | 16 | 1998/ 2002 | 2008 | 2015 | 2022 | 19 | 1,650 |
| NE-Alps / Hochschwab | AT | WEK | 2,065 | 47.63 | 15.16 | 16 | 2001 | 2008 | 2015 | 2022 | 19 | 1,650 |
| NE-Alps / Hochschwab | AT | GHK | 2,214 | 47.61 | 15.13 | 16 | 2001 | 2008 | 2015 | 2022 | 20 | 1,650 |
| NE-Alps / Hochschwab | AT | ZAK | 2,255 | 47.61 | 15.12 | 16 | 2001 | 2008 | 2015 | 2022 | 19 | 1,650 |
| E-Carpathians / Rodna Mountains | RO | GRO | 2,063 | 47.57 | 24.62 | 16 | 2001 | 2008 | 2015 | 2022 | 15–20 | 1,650 |
| E-Carpathians / Rodna Mountains | RO | GOL | 2,116 | 47.60 | 24.63 | 16 | 2001 | 2008 | 2015 | 2022 | 15–20 | 1,650 |
| E-Carpathians / Rodna Mountains | RO | BUH | 2,221 | 47.58 | 24.63 | 16 | 2001 | 2008 | 2015 | 2022 | 15–20 | 1,650 |
| E-Carpathians / Rodna Mountains | RO | REB | 2,268 | 47.59 | 24.64 | 16 | 2001 | 2008 | 2015 | 2022 | 13–20 | 1,650 |
| S-Alps / Dolomites | IT | GRM | 2,199 | 46.33 | 11.56 | 16 | 2001 | 2008 | 2015 | 2022 | 20 | 1,900 |
| S-Alps / Dolomites | IT | PNL | 2,463 | 46.38 | 11.61 | 14 | 2001 | 2008 | 2015 | 2022 | 20 | 1,900 |
| S-Alps / Dolomites | IT | RNK | 2,757 | 46.38 | 11.59 | 15 | 2001 | 2008 | 2015 | 2022 | 20 | 1,900 |
| S-Alps / Dolomites | IT | MTS | 2,893 | 46.52 | 11.81 | 15 | 2001 | 2008 | 2015 | 2022 | 20 | 1,900 |
| W-Alps / Alps of Valais-Entremont | CH | LAL | 2,360 | 46.03 | 7.25 | 16 | 2001 | 2008 | 2015 | 2022 | 20 | 2,080 |
| W-Alps / Alps of Valais-Entremont | CH | BRU | 2,550 | 46.02 | 7.20 | 16 | 2001 | 2008 | 2015 | 2022 | 20 | 2,080 |
| W-Alps / Alps of Valais-Entremont | CH | PAR | 2,989 | 46.00 | 7.23 | 5 | 2001 | 2008 | 2015 | 2022 | 19–20 | 2,080 |
| W-Alps / Alps of Valais-Entremont | CH | BOV | 3,212 | 45.99 | 7.24 | 2 | 2001 | 2008 | 2015 | 2022 | 20 | 2,080 |

**Supplementary Table 1.** Continued.

| Mountain region | Country | Summit | Elevation (m) | Latitude | Longitude | Number of plots | Year 1st survey | Year 2nd survey | Year 3rd survey | Year 4th survey | Soil temp. (years) | Forestline (m) |
|---|---|---|---|---|---|---|---|---|---|---|---|---|
| SW-Alps / Mercantour | FR | LAU | 2,508 | 44.33 | 6.91 | 16 | 2001 | | 2015 | 2022 | 13–16 | 2,100 |
| SW-Alps / Mercantour | FR | CFE | 2,700 | 44.32 | 6.94 | 12 | 2001 | | 2015 | 2022 | 16 | 2,100 |
| SW-Alps / Mercantour | FR | CBA | 2,792 | 44.30 | 6.94 | 8 | 2001 | | 2015 | 2022 | 16 | 2,100 |
| Northern Apennines | IT | FOG | 1,722 | 44.12 | 10.61 | 16 | 2001 | 2008 | 2015 | 2022 | 20 | 1,700 |
| Northern Apennines | IT | PCA | 1,815 | 44.20 | 10.69 | 16 | 2001 | 2008 | 2015 | 2022 | 16–20 | 1,700 |
| Northern Apennines | IT | MOM | 1,854 | 44.28 | 10.25 | 16 | 2001 | 2008 | 2015 | 2022 | 20 | 1,700 |
| Northern Apennines | IT | CAS | 1,978 | 44.33 | 10.21 | 16 | 2001 | 2008 | 2015 | 2022 | 20 | 1,700 |
| Central Pyrenees / Ordesa | ES | ACU | 2,242 | 42.64 | -0.06 | 16 | 2001 | 2008 | 2015 | 2022 | 20 | 2,150 |
| Central Pyrenees / Ordesa | ES | CUS | 2,519 | 42.65 | 0.03 | 16 | 2001 | 2008 | 2015 | 2022 | 16–20 | 2,150 |
| Central Pyrenees / Ordesa | ES | TOB | 2,779 | 42.66 | -0.01 | 5 | 2001 | 2008 | 2015 | 2022 | 10–20 | 2,150 |
| Central Pyrenees / Ordesa | ES | OLA | 3,022 | 42.66 | 0.05 | 4 | 2001 | 2008 | 2015 | 2022 | 16–20 | 2,150 |
| Central Caucasus / Kazbegi | GE | CP1 | 2,240 | 42.54 | 44.49 | 12 | 2001 | 2008 | 2015 | 2022 | 18–19 | 2,450 |
| Central Caucasus / Kazbegi | GE | CP2 | 2,477 | 42.50 | 44.46 | 16 | 2001 | 2008 | 2015 | 2022 | 12–19 | 2,450 |
| Central Caucasus / Kazbegi | GE | CP4 | 3,024 | 42.50 | 44.51 | 16 | 2001 | 2008 | 2015 | 2022 | 19 | 2,450 |

**Supplementary Table 1.** Continued.

| Mountain region | Country | Summit | Elevation (m) | Latitude | Longitude | Number of plots | Year 1st survey | Year 2nd survey | Year 3rd survey | Year 4th survey | Soil temp. (years) | Forestline (m) |
|---|---|---|---|---|---|---|---|---|---|---|---|---|
| Central Apennines / Majella | IT | FEM | 2,405 | 42.03 | 14.10 | 16 | 2001 | 2008 | 2015 | 2022 | 20 | 1,800 |
| Central Apennines / Majella | IT | MAC | 2,635 | 42.06 | 14.10 | 16 | 2001 | 2008 | 2015 | 2022 | 20 | 1,800 |
| Central Apennines / Majella | IT | MAM | 2,737 | 42.10 | 14.12 | 16 | 2001 | 2008 | 2015 | 2022 | 17–20 | 1,800 |
| W-Sierra Nevada | ES | PUL | 2,778 | 37.04 | -3.35 | 16 | 2001 | 2008 | 2015 | 2022 | 20 | 2,000 |
| W-Sierra Nevada | ES | CUP | 2,968 | 37.05 | -3.35 | 16 | 2001 | 2008 | 2015 | 2022 | 17–19 | 2,000 |
| W-Sierra Nevada | ES | TCA | 3,150 | 37.04 | -3.40 | 16 | 2001 | 2008 | 2015 | 2022 | 19–20 | 2,000 |
| W-Sierra Nevada | ES | MAC | 3,327 | 37.06 | -3.36 | 7 | 2001 | 2008 | 2015 | 2022 | 19–20 | 2,000 |
| Lefka Ori | GR | LOW | 1,664 | 35.27 | 24.09 | 12 | 2001 | 2008 | 2015 | 2022 | 15 | 1,450 |
| Lefka Ori | GR | CHO | 1,965 | 35.27 | 24.08 | 16 | 2001 | 2008 | 2015 | 2022 | 20 | 1,450 |
| Lefka Ori | GR | SEK | 2,160 | 35.28 | 24.09 | 13 | 2001 | 2008 | 2015 | 2022 | 13–20 | 1,450 |
| Lefka Ori | GR | STR | 2,339 | 35.30 | 24.06 | 8 | 2001 | 2008 | 2015 | 2022 | 11–16 | 1,450 |

**Supplementary Table 2.** Overview of the taxa included in this study, as well as their assigned elevational ranks (sensu Gottfried et al. [2012], herein referred to as "altitudinal rank"), representing their elevational distribution optima along a gradient from the nival (1) to the colline (7) elevational vegetation belt. The considered elevational vegetation belts and their associated elevational ranks were: (1) nival: extending from the permanent snowline upwards, very sparse vegetation; (2) subnival: transition zone between the nival and alpine belts, patchy vegetation; (3) alpine: above the climatic treeline, closed vegetation cover; (4) subalpine: transition zone between the alpine and montane belts, stunted trees (Krummholz); (5) montane: extends from mid-elevation forests to the treeline; (6) submontane: transition zone between the montane and colline belts; (7) colline: foothills. The assignment of elevational ranks was based on the vertical distribution ranges of taxa as indicated in regional floras, as well as regional expert judgements to harmonize different schemes and indications. ER ... elevational rank

| Taxon | ER | Taxon | ER |
|---|---|---|---|
| Achillea atrata | 3 | Anthoxanthum nipponicum | 4 |
| Achillea clavennae | 4 | Anthoxanthum odoratum | 5 |
| Achillea erba-rotta subsp. moschata | 3 | Anthyllis vulneraria | 5 |
| Achillea millefolium | 6 | Anthyllis vulneraria subsp. alpestris | 5 |
| Achillea millefolium subsp. collina | 6 | Anthyllis vulneraria subsp. pseudoarundana | 3 |
| Achillea oxyloba subsp. oxyloba | 3 | Anthyllis vulneraria subsp. pulchella | 5 |
| Aethionema saxatile subsp. creticum | 5 | Anthyllis vulneraria subsp. variegata | 4 |
| Aethionema saxatile subsp. ovalifolium | 5 | Aquilegia alpina | 4 |
| Agrostis alpina | 3 | Arabis alpina subsp. alpina | 4 |
| Agrostis capillaris | 6 | Arabis caerulea | 2 |
| Agrostis rupestris | 3 | Arabis pumila | 4 |
| Agrostis rupestris subsp. pyrenaica | 2 | Arabis scabra | 6 |
| Agrostis vinealis | 5 | Arabis stellulata | 4 |
| Ajuga pyramidalis | 5 | Arctostaphylos uva-ursi | 5 |
| Alopecurus glacialis | 3 | Arctous alpina | 4 |
| Alyssum cuneifolium | 3 | Arenaria ciliata | 2 |
| Alyssum minutum | 6 | Arenaria cretica | 4 |
| Alyssum sphacioticum | 4 | Arenaria grandiflora | 4 |
| Androsace adfinis subsp. puberula | 1 | Arenaria grandiflora subsp. grandiflora | 4 |
| Androsace albimontana | 3 | Arenaria multicaulis subsp. moehringioides | 3 |
| Androsace alpina | 1 | Arenaria pungens | 3 |
| Androsace chamaejasme | 3 | Arenaria purpurascens | 4 |
| Androsace ciliata | 3 | Arenaria saponarioides | 6 |
| Androsace lactea | 4 | Arenaria tetraquetra subsp. amabilis | 3 |
| Androsace obtusifolia | 3 | Armeria alpina | 4 |
| Androsace villosa | 4 | Armeria arenaria subsp. marginata | 4 |
| Androsace villosa subsp. villosa | 4 | Armeria canescens | 5 |
| Andryala agardhii | 5 | Arnica montana subsp. montana | 5 |
| Anemonastrum fasciculatum | 4 | Artemisia genipi | 2 |
| Anemonastrum narcissiflorum | 5 | Artemisia umbelliformis subsp. eriantha | 3 |
| Anemonoides baldensis | 3 | Asplenium viride | 5 |
| Anemonoides nemorosa | 6 | Aster alpinus | 4 |
| Antennaria carpatica | 3 | Aster bellidiastrum | 5 |
| Antennaria caucasica | 3 | Astragalus alpinus | 4 |
| Antennaria dioica | 5 | Astragalus angustifolius subsp. angustifolius | 4 |
| Anthemis cretica subsp. iberica | 5 | Astragalus australis | 3 |

**Supplementary Table 2.** Continued.

| Taxon | ER | Taxon | ER |
|---|---|---|---|
| Astrantia minor | 6 | Carex atrata subsp. atrata | 4 |
| Athamanta cretensis | 5 | Carex bigelowii | 2 |
| Aubrieta deltoidea | 5 | Carex capillaris | 4 |
| Avenella flexuosa subsp. iberica | 4 | Carex capillifolia | 3 |
| Bartsia alpina | 4 | Carex curvula subsp. curvula | 3 |
| Bellardiochloa variegata | 5 | Carex digitata | 6 |
| Berberis cretica | 5 | Carex ericetorum | 5 |
| Betonica macrantha | 5 | Carex firma | 4 |
| Betula nana | 5 | Carex fuliginosa | 3 |
| Betula pubescens | 6 | Carex humilis | 6 |
| Biscutella glacialis | 4 | Carex kitaibeliana | 5 |
| Biscutella laevigata subsp. austriaca | 5 | Carex montana | 6 |
| Biscutella laevigata subsp. laevigata | 5 | Carex ornithopoda subsp. ornithopoda | 6 |
| Bistorta officinalis | 5 | Carex ornithopoda subsp. ornithopodioides | 4 |
| Bistorta vivipara | 4 | Carex parviflora | 3 |
| Botrychium lunaria | 5 | Carex pilulifera | 7 |
| Brachypodium pinnatum | 4 | Carex rupestris | 3 |
| Bromus squarrosus | 5 | Carex sempervirens | 4 |
| Bromus tectorum | 6 | Carex tristis | 2 |
| Bromus variegatus | 5 | Carlina acaulis | 6 |
| Bufonia stricta subsp. stricta | 5 | Carum carvi | 6 |
| Bupleurum ranunculoides | 4 | Carum caucasicum | 3 |
| Bupleurum ranunculoides subsp. telonense | 4 | Centaurea cheiranthifolia | 5 |
| Bupleurum trichopodum | 6 | Centaurea idaea | 5 |
| Calamagrostis varia | 6 | Centaurea nervosa | 3 |
| Calluna vulgaris | 6 | Centaurea raphanina subsp. raphanina | 5 |
| Campanula alpina | 4 | Cephalaria gigantea | 5 |
| Campanula barbata | 5 | Cerastium alpinum | 4 |
| Campanula cochleariifolia | 5 | Cerastium arvense | 5 |
| Campanula collina | 5 | Cerastium arvense subsp. strictum | 4 |
| Campanula pulla | 4 | Cerastium arvense subsp. suffruticosum | 5 |
| Campanula rotundifolia | 6 | Cerastium cerastoides | 3 |
| Campanula scheuchzeri | 4 | Cerastium fontanum subsp. fontanum | 4 |
| Campanula sibirica subsp. hohenackeri | 5 | Cerastium fontanum subsp. vulgare | 6 |
| Campanula tridentata subsp. biebersteiniana | 3 | Cerastium gracile | 4 |
| Campanula willkommii | 3 | Cerastium multiflorum | 2 |
| Cardamine bellidifolia | 2 | Cerastium purpurascens | 3 |
| Cardamine resedifolia | 2 | Cerastium semidecandrum | 6 |
| Carduus carlinoides subsp. carlinoides | 4 | Cerastium thomasii | 3 |
| Carduus carlinoides subsp. hispanicus | 3 | Cerastium tomentosum | 5 |
| Carduus defloratus | 5 | Cerastium uniflorum | 1 |
| Carduus defloratus subsp. carlinifolius | 5 | Chaenorhinum glareosum | 2 |
| Carduus defloratus subsp. defloratus | 5 | Chaerophyllum aureum | 6 |
| Carex aterrima subsp. medwedewii | 2 | Chamorchis alpina | 4 |

**Supplementary Table 2.** Continued.

| Taxon | ER | Taxon | ER |
|---|---|---|---|
| Cherleria circassica | 4 | Draba aizoides subsp. aizoides | 4 |
| Cherleria sedoides | 3 | Draba cretica | 4 |
| Cicer incisum | 4 | Draba dolomitica | 3 |
| Cicerbita racemosa | 4 | Draba dubia | 2 |
| Cirsium acaule subsp. acaule | 6 | Draba hispanica subsp. laderoi | 3 |
| Cirsium obvallatum | 4 | Draba hispida | 5 |
| Clematis alpina | 5 | Draba hoppeana | 3 |
| Clinopodium alpinum | 5 | Draba sauteri | 3 |
| Clinopodium alpinum subsp. meridionale | 5 | Draba siliquosa | 3 |
| Colchicum montanum | 5 | Draba stellata | 3 |
| Comastoma tenellum | 3 | Draba supranivalis | 1 |
| Crepis aurea subsp. aurea | 5 | Draba tomentosa | 2 |
| Crepis aurea subsp. glabrescens | 4 | Draba verna | 6 |
| Crepis jacquinii subsp. kerneri | 4 | Dryas caucasica | 3 |
| Crepis magellensis | 3 | Dryas octopetala | 3 |
| Crepis oporinoides | 3 | Edraianthus graminifolius | 5 |
| Crepis pygmaea | 3 | Empetrum nigrum subsp. caucasicum | 5 |
| Crepis sibthorpiana | 4 | Empetrum nigrum subsp. hermaphroditum | 5 |
| Crepis terglouensis | 3 | Erica carnea | 6 |
| Crocus nudiflorus | 6 | Erigeron acris subsp. acris | 5 |
| Crocus vernus | 6 | Erigeron caucasicus subsp. caucasicus | 1 |
| Cruciata glabra | 6 | Erigeron epiroticus | 5 |
| Cuscuta epithymum subsp. epithymum | 6 | Erigeron major | 3 |
| Cynanchica aristata subsp. scabra | 5 | Erigeron neglectus | 3 |
| Cynanchica idaea | 5 | Erigeron uniflorus | 2 |
| Cynoglossum officinale | 5 | Erinus alpinus | 4 |
| Cytisus balansae | 5 | Eritrichium nanum subsp. nanum | 2 |
| Dactylis glomerata subsp. juncinella | 3 | Erodium cheilanthifolium | 4 |
| Dactylorhiza viridis | 5 | Eryngium glaciale | 3 |
| Danthonia decumbens | 6 | Erysimum nevadense | 5 |
| Daphne mezereum | 7 | Euphorbia acanthothamnos | 5 |
| Daphne striata | 4 | Euphorbia herniariifolia | 4 |
| Deschampsia flexuosa | 5 | Euphorbia nevadensis | 4 |
| Dianthus alpinus | 3 | Euphorbia oblongifolia | 6 |
| Dianthus benearnensis | 6 | Facchinia cerastiifolia | 2 |
| Dianthus monspessulanus subsp. monspessulanus | 6 | Facchinia cherlerioides | 2 |
| Dianthus nudiflorus | 6 | Festuca airoides | 4 |
| Dianthus pavonius | 5 | Festuca alpina | 3 |
| Dianthus pungens subsp. brachyanthus | 4 | Festuca circummediterranea | 5 |
| Diapensia lapponica | 2 | Festuca clementei | 1 |
| Dioscorea pyrenaica | 5 | Festuca eskia | 4 |
| Doronicum clusii | 3 | Festuca gautieri | 4 |
| Doronicum glaciale subsp. calcareum | 3 | Festuca glacialis | 3 |
| Draba aizoides | 4 | Festuca halleri subsp. halleri | 4 |

**Supplementary Table 2.** Continued.

| Taxon | ER | Taxon | ER |
|---|---|---|---|
| Festuca indigesta | 4 | Gentianella anisodonta | 5 |
| Festuca intercedens | 2 | Gentianella campestris | 5 |
| Festuca nigrescens | 5 | Gentianella campestris subsp. campestris | 5 |
| Festuca norica | 4 | Gentianella caucasea | 4 |
| Festuca ovina | 5 | Gentianella germanica | 6 |
| Festuca paniculata | 4 | Geranium cinereum | 4 |
| Festuca picturata | 4 | Geranium gymnocaulon | 4 |
| Festuca polita | 5 | Geranium ibericum | 5 |
| Festuca pseudoeskia | 3 | Geranium ruprechtii | 6 |
| Festuca pyrenaica | 3 | Geranium sylvaticum | 6 |
| Festuca quadriflora | 3 | Geum montanum | 4 |
| Festuca robustifolia | 4 | Geum reptans | 2 |
| Festuca rupicaprina | 4 | Gymnadenia conopsea | 6 |
| Festuca scabriculmis | 4 | Gymnadenia nigra | 3 |
| Festuca varia | 4 | Hedysarum hedysaroides | 4 |
| Festuca versicolor subsp. brachystachys | 4 | Hedysarum hedysaroides subsp. hedysaroides | 4 |
| Festuca violacea subsp. italica | 4 | Helianthemum nummularium subsp. glabrum | 5 |
| Festuca violacea subsp. violacea | 5 | Helianthemum oelandicum subsp. alpestre | 4 |
| Filago arvensis | 7 | Helictochloa hookeri | 5 |
| Fritillaria lutea | 3 | Helictochloa versicolor | 4 |
| Galium anisophyllon | 4 | Helictochloa versicolor subsp. praetutiana | 4 |
| Galium magellense | 4 | Helictochloa versicolor subsp. versicolor | 4 |
| Galium marchandii | 4 | Helictotrichon cycladum | 6 |
| Galium murale | 7 | Helictotrichon sedenense | 4 |
| Galium noricum | 4 | Herniaria boissieri | 3 |
| Galium pyrenaicum | 4 | Herniaria parnassica subsp. cretica | 5 |
| Galium rosellum | 3 | Hippocrepis comosa | 6 |
| Galium saxatile | 6 | Homogyne alpina | 4 |
| Galium verticillatum | 6 | Homogyne discolor | 4 |
| Galium verum | 7 | Hormathophylla purpurea | 1 |
| Genista tinctoria | 5 | Hormathophylla spinosa | 4 |
| Gentiana acaulis | 5 | Hornungia alpina | 4 |
| Gentiana aquatica | 4 | Huperzia selago | 4 |
| Gentiana brachyphylla subsp. brachyphylla | 3 | Huperzia selago subsp. selago | 4 |
| Gentiana brachyphylla subsp. favratii | 3 | Hypericum empetrifolium | 5 |
| Gentiana clusii | 4 | Hypericum maculatum | 6 |
| Gentiana frigida | 2 | Hypericum richeri subsp. richeri | 3 |
| Gentiana nivalis | 4 | Hypochaeris uniflora | 4 |
| Gentiana pumila subsp. pumila | 4 | Iberis carnosa subsp. embergeri | 2 |
| Gentiana purpurea | 5 | Iberis saxatilis subsp. saxatilis | 3 |
| Gentiana septemfida | 4 | Jacobaea boissieri | 3 |
| Gentiana terglouensis | 3 | Jacobaea incana | 3 |
| Gentiana verna subsp. pontica | 4 | Jasione crispa subsp. amethystina | 3 |
| Gentiana verna subsp. verna | 5 | Jasione montana | 6 |

**Supplementary Table 2.** Continued.

| Taxon | ER | Taxon | ER |
|---|---|---|---|
| Juniperus communis | 5 | Luzula spicata subsp. conglomerata | 3 |
| Juniperus oxycedrus | 6 | Luzula stenophylla | 3 |
| Jurinea humilis | 5 | Luzula sylvatica subsp. sieberi | 4 |
| Jurinea moschus | 1 | Lycopodium alpinum | 4 |
| Kalmia procumbens | 4 | Mattiastrum lithospermifolium subsp. cariense | 4 |
| Knautia calycina | 5 | Melica rectiflora | 5 |
| Knautia dipsacifolia subsp. dipsacifolia | 5 | Minuartia hirsuta subsp. oreina | 4 |
| Kobresia myosuroides | 2 | Minuartia recurva subsp. recurva | 3 |
| Kobresia simpliciuscula | 3 | Muscari spreitzenhoferi | 5 |
| Lactuca alpestris | 5 | Mutellina purpurea | 4 |
| Lactuca singularis | 4 | Myosotis alpestris | 4 |
| Lapsana communis subsp. grandiflora | 6 | Myosotis ambigens | 4 |
| Larix decidua | 5 | Myosotis arvensis | 5 |
| Leontodon boryi | 3 | Myosotis minutiflora | 5 |
| Leontodon caucasicus | 3 | Nardus stricta | 5 |
| Leontodon hispidus | 5 | Neogaya simplex | 3 |
| Leontopodium nivale | 3 | Noccaea alpestris | 4 |
| Leontopodium nivale subsp. alpinum | 3 | Noccaea caerulescens subsp. brachypetala | 6 |
| Lepidium hirtum subsp. stylatum | 2 | Noccaea rotundifolia | 3 |
| Leucanthemopsis alpina subsp. alpina | 2 | Odontarrhena fragillima | 4 |
| Leucanthemopsis alpina subsp. minima | 4 | Omalotheca supina | 3 |
| Leucanthemopsis alpina subsp. tatrae | 2 | Orchis mascula | 7 |
| Leucanthemopsis pectinata | 3 | Oreochloa disticha | 2 |
| Leucanthemum atratum | 4 | Oreojuncus monanthos | 3 |
| Lilium martagon | 6 | Oreojuncus trifidus | 3 |
| Linaria aeruginea subsp. nevadensis | 3 | Oreomecon alpina subsp. alpina | 3 |
| Linaria alpina | 2 | Oxyria digyna | 3 |
| Linum alpinum subsp. gracilius | 5 | Oxytropis albana | 5 |
| Linum hypericifolium | 5 | Oxytropis campestris | 5 |
| Lomatocarum alpinum | 3 | Oxytropis campestris subsp. campestris | 4 |
| Lomelosia caucasica | 5 | Oxytropis montana | 3 |
| Lotus alpicola | 5 | Oxytropis neglecta subsp. neglecta | 4 |
| Lotus alpinus | 4 | Parnassia palustris | 5 |
| Lotus corniculatus | 5 | Paronychia macrosepala | 5 |
| Lotus glacialis | 2 | Paronychia polygonifolia | 4 |
| Luzula alpinopilosa subsp. obscura | 3 | Pedicularis elegans | 5 |
| Luzula arcuata | 2 | Pedicularis kerneri | 3 |
| Luzula campestris | 7 | Pedicularis lapponica | 4 |
| Luzula glabrata | 4 | Pedicularis portenschlagii | 3 |
| Luzula lutea | 4 | Pedicularis rosea | 3 |
| Luzula luzuloides | 6 | Pedicularis rostratocapitata | 3 |
| Luzula multiflora | 5 | Pedicularis rostratocapitata subsp. rostratocapitata | 3 |
| Luzula nivea | 6 | Pedicularis tuberosa | 4 |
| Luzula spicata | 2 | Pedicularis verticillata | 4 |

**Supplementary Table 2.** Continued.

| Taxon | ER | Taxon | ER |
|---|---|---|---|
| Pentanema orientale | 5 | Potentilla aurea subsp. chrysocraspeda | 4 |
| Petrocallis pyrenaica | 3 | Potentilla brauneana | 3 |
| Petrosedum tenuifolium | 4 | Potentilla clusiana | 3 |
| Peucedanum alpinum | 4 | Potentilla crantzii | 4 |
| Phedimus spurius | 5 | Potentilla erecta | 5 |
| Phleum alpinum | 5 | Potentilla frigida | 2 |
| Phleum hirsutum | 5 | Potentilla grandiflora | 4 |
| Phyllodoce caerulea | 4 | Potentilla neumanniana | 7 |
| Phyteuma betonicifolium | 5 | Potentilla nitida | 3 |
| Phyteuma globulariifolium | 3 | Potentilla nivalis | 4 |
| Phyteuma hemisphaericum | 3 | Potentilla reptans | 7 |
| Phyteuma orbiculare | 5 | Primula algida | 4 |
| Phyteuma sieberi | 3 | Primula amoena | 5 |
| Picea abies | 6 | Primula apennina | 3 |
| Pimpinella procumbens | 3 | Primula auricula | 4 |
| Pimpinella rhodantha | 5 | Primula clusiana | 4 |
| Pimpinella saxifraga | 6 | Primula hirsuta | 4 |
| Pimpinella tragium subsp. depressa | 4 | Primula minima | 3 |
| Pinguicula alpina | 4 | Primula veris subsp. columnae | 6 |
| Pinus cembra | 5 | Prunella vulgaris | 7 |
| Pinus mugo | 5 | Prunus prostrata | 4 |
| Plantago alpina | 4 | Pseudocherleria imbricata | 4 |
| Plantago atrata | 4 | Pseudocherleria inamoena | 1 |
| Plantago lanceolata | 6 | Pseudorchis albida | 5 |
| Plantago maritima | 4 | Pulmonaria angustifolia | 5 |
| Plantago monosperma | 4 | Pulsatilla alpina | 3 |
| Plantago subulata | 4 | Pulsatilla alpina subsp. alpina | 4 |
| Poa alpina | 3 | Pulsatilla vernalis | 3 |
| Poa bulbosa | 6 | Pyrola minor | 5 |
| Poa caucasica | 2 | Ranunculus acris | 5 |
| Poa granitica subsp. disparilis | 3 | Ranunculus alpestris subsp. alpestris | 4 |
| Poa laxa | 1 | Ranunculus apenninus | 4 |
| Poa ligulata | 4 | Ranunculus breyninus | 4 |
| Poa longifolia | 5 | Ranunculus carinthiacus | 4 |
| Poa molinerii | 5 | Ranunculus crenatus | 3 |
| Poa ursina | 3 | Ranunculus demissus | 4 |
| Polygala alpestris | 5 | Ranunculus glacialis | 1 |
| Polygala alpestris subsp. alpestris | 5 | Ranunculus hybridus | 4 |
| Polygala alpicola | 4 | Ranunculus lojkae | 3 |
| Polygaloides chamaebuxus | 6 | Ranunculus montanus | 4 |
| Polygonatum verticillatum | 6 | Ranunculus parnassifolius subsp. heterocarpus | 3 |
| Polygonum aviculare | 7 | Ranunculus seguieri subsp. seguieri | 3 |
| Polygonum carneum | 4 | Ranunculus villarsii | 4 |
| Potentilla aurea | 4 | Reseda complicata | 4 |

**Supplementary Table 2.** Continued.

| Taxon | ER | Taxon | ER |
|---|---|---|---|
| Rhamnus oleoides | 6 | Saxifraga paniculata | 4 |
| Rhinanthus mediterraneus | 5 | Saxifraga sedoides | 3 |
| Rhinanthus minor | 6 | Saxifraga sibirica | 5 |
| Rhododendron caucasicum | 4 | Saxifraga squarrosa | 3 |
| Rhododendron ferrugineum | 5 | Scabiosa lucida | 5 |
| Rhododendron hirsutum | 5 | Scorzonera aristata | 5 |
| Rhododendron kotschyi | 5 | Scorzoneroides helvetica | 4 |
| Rhododendron × intermedium | 5 | Scorzoneroides montana | 3 |
| Rosa pendulina | 6 | Scorzoneroides pyrenaica | 4 |
| Rubus saxatilis | 6 | Scrophularia minima | 2 |
| Rumex acetosa | 6 | Sedum album | 6 |
| Rumex alpestris | 5 | Sedum alpestre | 3 |
| Rumex alpinus | 4 | Sedum atratum | 4 |
| Sabulina attica | 4 | Sedum dasyphyllum | 5 |
| Sabulina verna subsp. verna | 3 | Sedum monregalense | 5 |
| Sagina glabra | 4 | Sedum pallidum | 5 |
| Salix alpina | 5 | Sedum tristriatum | 5 |
| Salix glauca | 5 | Selaginella selaginoides | 4 |
| Salix hastata | 5 | Sempervivum arachnoideum | 5 |
| Salix herbacea | 3 | Sempervivum arachnoideum subsp. arachnoideum | 5 |
| Salix kazbekensis | 5 | Sempervivum minutum | 3 |
| Salix phylicifolia | 5 | Sempervivum montanum | 4 |
| Salix reticulata | 3 | Sempervivum montanum subsp. burnatii | 4 |
| Salix retusa | 3 | Sempervivum montanum subsp. montanum | 4 |
| Salix serpillifolia | 2 | Sempervivum tectorum | 5 |
| Salix waldsteiniana | 5 | Sesleria albicans | 5 |
| Sanguisorba minor | 7 | Sesleria juncifolia | 4 |
| Saponaria caespitosa | 5 | Sesleria ovata | 2 |
| Satureja spinosa | 4 | Sesleria sphaerocephala | 3 |
| Saussurea pygmaea | 3 | Sibbaldia parviflora | 2 |
| Saxifraga aizoides | 5 | Sideritis glacialis | 3 |
| Saxifraga androsacea | 2 | Silene acaulis | 2 |
| Saxifraga bryoides | 1 | Silene acaulis subsp. acaulis | 2 |
| Saxifraga caesia | 3 | Silene acaulis subsp. bryoides | 2 |
| Saxifraga carpatica | 3 | Silene boryi | 5 |
| Saxifraga cespitosa | 2 | Silene rupestris | 5 |
| Saxifraga exarata | 2 | Silene saxatilis | 4 |
| Saxifraga exarata subsp. ampullacea | 3 | Silene vulgaris subsp. vulgaris | 5 |
| Saxifraga exarata subsp. exarata | 2 | Soldanella alpina | 4 |
| Saxifraga facchinii | 3 | Soldanella carpatica | 4 |
| Saxifraga moschata | 2 | Soldanella minima subsp. austriaca | 3 |
| Saxifraga nevadensis | 1 | Soldanella rugosa | 3 |
| Saxifraga oppositifolia | 1 | Solidago virgaurea | 5 |
| Saxifraga oppositifolia subsp. oppositifolia | 1 | Solidago virgaurea subsp. minuta | 5 |

**Supplementary Table 2.** Continued.

| Taxon | ER | Taxon | ER |
|---|---|---|---|
| Sorbus aucuparia | 6 | Vaccinium vitis-idaea | 5 |
| Swertia iberica | 5 | Valeriana celtica | 3 |
| Tanacetum coccineum subsp. coccineum | 6 | Valeriana elongata | 4 |
| Teucrium aureum subsp. turdetanum | 4 | Valeriana montana | 5 |
| Thalictrum alpinum | 3 | Valeriana saliunca | 3 |
| Thesium alpinum | 5 | Valeriana tripteris | 6 |
| Thesium bergeri | 6 | Verbascum spinosum | 6 |
| Thlaspi stylosum | 5 | Veronica allionii | 3 |
| Thymbra capitata | 6 | Veronica alpina | 2 |
| Thymelaea tinctoria | 5 | Veronica aphylla | 3 |
| Thymus armeniacus | 5 | Veronica baumgartenii | 4 |
| Thymus nervosus | 4 | Veronica bellidioides | 3 |
| Thymus nummularius | 5 | Veronica bellidioides subsp. bellidioides | 3 |
| Thymus praecox subsp. polytrichus | 4 | Veronica chamaedrys subsp. chamaedrys | 7 |
| Thymus serpylloides subsp. serpylloides | 3 | Veronica fruticans | 4 |
| Thymus transcaucasicus | 5 | Veronica gentianoides | 4 |
| Tragopogon filifolius | 5 | Veronica officinalis | 6 |
| Tragopogon reticulatus | 5 | Veronica telephiifolia | 1 |
| Trifolium alpinum | 4 | Veronica thymifolia | 4 |
| Trifolium ambiguum | 5 | Vicia alpestris | 4 |
| Trifolium badium | 5 | Vicia pyrenaica | 4 |
| Trifolium canescens | 5 | Vicia sepium | 7 |
| Trifolium pratense | 5 | Vicia tenuifolia subsp. subalpina | 5 |
| Trifolium repens | 6 | Viola alpina | 3 |
| Trifolium thalii | 4 | Viola biflora | 4 |
| Trifolium trichocephalum | 5 | Viola calcarata | 4 |
| Trinia dalechampii | 5 | Viola cavillieri | 4 |
| Tripleurospermum caucasicum | 1 | Viola crassiuscula | 2 |
| Trisetum glaciale | 2 | Viola eugeniae subsp. eugeniae | 5 |
| Trollius europaeus | 5 | Viola magellensis | 3 |
| Trollius ranunculinus | 4 | Viola odorata | 7 |
| Vaccinium myrtillus | 6 | Viola rupestris subsp. rupestris | 6 |
| Vaccinium uliginosum | 6 | Vitaliana primuliflora subsp. canescens | 2 |
| Vaccinium uliginosum subsp. uliginosum | 4 | Vitaliana primuliflora subsp. praetutiana | 3 |

**Supplementary Table 3.** Overview of the predictor variables used across any of the fitted models, including the reasoning for their inclusion. Rates of change in the various temperature metrics were calculated from both macroclimatic and microclimatic temperature data.

| Predictor variable | | Relevance |
|---|---|---|
| Rate of change in | Mean annual temperature ($T_{mean}$ annual) | Integrates over several temperature variables likely affecting plant survival. |
| | Mean, minimum and maximum summer temperature ($T_{mean}$, $T_{min}$, $T_{max}$ summer) | Affect plant growth and reproduction during the growing season. |
| | Mean, minimum and maximum winter temperature ($T_{mean}$, $T_{min}$, $T_{max}$ winter) | Important determinants for plant survival during winter dormancy. |
| | Mean, minimum and maximum June temperature ($T_{mean}$, $T_{min}$, $T_{max}$ June) | Minimum June temperature has previously been shown to correlate with thermophilization in mountain plant communities (Gottfried et al. 2012). |
| Bare soil cover | | Expresses the 'competition-free' space available for colonization by plants. |
| Rock & scree cover | | Quantifies substrate constraints on colonization. |
| Availability of thermophilic colonizers | | Quantifies how likely thermophilization of the focal plot can occur via neutral processes, i.e. the probability that a new taxon colonizing from the immediate neighborhood will increase the average elevational rank of the plot. |

**Supplementary Table 4.** Summaries of intercept-only linear mixed-effects models with either thermophilization rate or the rates of change in one of the ten temperature metrics on both climatic scales (macro- and microclimate) as response. The models included a random-intercept term for plot cluster nested in summit nested in mountain region for the thermophilization rate, summit nested in mountain region for microclimatic temperature metrics and only mountain region for macroclimatic ones. Scale indicates which version of the temperature metric was used (macro- or microclimatic). Only the Bonferroni-adjusted, one-sided, right-tailed p values are reported (this adjustment can lead to p values of exactly 1). Bold p values indicate statistical significance. Est. ... estimated intercept; S.E. ... standard error; $T_{mean}$ ... mean temperature; $T_{min}$ ... minimum temperature; $T_{max}$ ... maximum temperature.

| Response | Scale | Est. | S.E. | z value | p value |
|---|---|---|---|---|---|
| Thermophilization rate | | 0.052 | 0.017 | 3.092 | < **0.001** |
| $T_{mean}$ Annual | macro | 0.484 | 0.046 | 10.512 | < **0.001** |
| | micro | 0.415 | 0.064 | 6.447 | < **0.001** |
| $T_{mean}$ Summer | macro | 0.419 | 0.048 | 8.684 | < **0.001** |
| | micro | 0.313 | 0.086 | 3.623 | **0.001** |
| $T_{min}$ Summer | macro | 0.073 | 0.061 | 1.187 | 1.000 |
| | micro | -0.046 | 0.059 | -0.772 | 1.000 |
| $T_{max}$ Summer | macro | 0.512 | 0.077 | 6.671 | < **0.001** |
| | micro | 0.661 | 0.305 | 2.166 | 0.152 |
| $T_{mean}$ Winter | macro | 0.634 | 0.066 | 9.651 | < **0.001** |
| | micro | 0.542 | 0.085 | 6.347 | < **0.001** |
| $T_{min}$ Winter | macro | 0.877 | 0.169 | 5.196 | < **0.001** |
| | micro | 0.768 | 0.123 | 6.263 | < **0.001** |
| $T_{max}$ Winter | macro | 0.436 | 0.034 | 12.731 | < **0.001** |
| | micro | 0.522 | 0.086 | 6.098 | < **0.001** |
| $T_{mean}$ June | macro | 0.548 | 0.107 | 5.124 | < **0.001** |
| | micro | 0.382 | 0.129 | 2.956 | **0.016** |
| $T_{min}$ June | macro | 0.522 | 0.094 | 5.533 | < **0.001** |
| | micro | 0.252 | 0.095 | 2.659 | **0.039** |
| $T_{max}$ June | macro | 0.576 | 0.123 | 4.663 | < **0.001** |
| | micro | 0.657 | 0.278 | 2.360 | 0.091 |

## Geographical and elevational trends

To test whether there were geographical or elevational trends in the rates of thermophilization and temperature change, we fitted linear mixed-effects models, regressing either plot-level thermophilization, plot-cluster-level microclimatic or summit-level macroclimatic temperature change, separately for each of the ten temperature metrics, against the summits' latitudes, longitudes and elevational distances to the current regional forestlines. The elevational distance to the current regional forestline was used to account for the variation of treeline elevation across the studied mountain ranges. It was calculated as the elevational distance of a summit to the elevation of the current forestline in the respective mountain region, with the current position of the regional forestlines estimated by the regional data contributors.

These models included random intercept terms for plot cluster nested in summit nested in mountain region for models fitted to plot-level thermophilization rates, summit nested in mountain region for models fitted to plot-cluster-level microclimatic temperature change and only mountain region for models fitted to summit-level macroclimatic temperature change. To correct for performing multiple hypothesis tests, the resulting p values were adjusted using the Bonferroni method. This adjustment was applied separately to the models of the macro- and microclimatic metrics (ten each).

We found no significant relationship between the rate of thermophilization and latitudinal, longitudinal or elevational gradients. However, significant relationships regarding the rates of temperature change along latitudinal, longitudinal and elevational gradients were present in a few temperature metrics with stronger warming towards the east and the north of Europe and towards higher elevations (Supplementary Table 5).

**Supplementary Table 5.** Summaries of linear mixed-effects models relating the plot-level thermophilization rate (i.e. change in cover-weighted mean elevational rank), as well as the rates of change in the ten temperature metrics on both climatic scales to the summits' latitude (degrees), longitude (degrees) and elevation (expressed as the difference between the summits' elevations and the elevations of the current regional forestlines; expressed in kilometers to keep estimated effects interpretable). The models included a random-intercept term for plot cluster nested in summit nested in mountain region for thermophilization rate, summit nested in mountain region for microclimatic temperature metrics and only mountain region for macroclimatic ones. Scale indicates which version of the temperature metric was used (micro- or macroclimatic). Only the Bonferroni-adjusted, one-sided, right-tailed p values are reported (this adjustment can lead to p values of exactly 1). Bold p values indicate statistical significance. Est. ... estimated effect; S.E. ... standard error; $T_{mean}$ ... mean temperature; $T_{min}$ ... minimum temperature; $T_{max}$ ... maximum temperature.

| Response | Scale | Predictor | Est. | S.E. | z value | p value |
|---|---|---|---|---|---|---|
| Thermophilization rate | | Longitude | 0.000 | 0.019 | 0.007 | 0.994 |
| | | Latitude | -0.004 | 0.018 | -0.246 | 0.805 |
| | | Elevation | -0.012 | 0.017 | -0.690 | 0.490 |
| $T_{mean}$ Annual | macro | Longitude | 0.072 | 0.037 | 1.924 | 0.543 |
| | | Latitude | -0.026 | 0.040 | -0.663 | 1.000 |
| | | Elevation | 0.002 | 0.002 | 1.175 | 1.000 |
| | micro | Longitude | 0.102 | 0.056 | 1.813 | 0.698 |
| | | Latitude | -0.020 | 0.056 | -0.363 | 1.000 |
| | | Elevation | 0.126 | 0.042 | 2.997 | **0.027** |

**Supplementary Table 5.** Continued.

| | | | | | | |
|---|---|---|---|---|---|---|
| $T_{mean}$ Summer | macro | Longitude | 0.023 | 0.037 | 0.637 | 1.000 |
| | | Latitude | -0.093 | 0.040 | -2.332 | 0.197 |
| | | Elevation | 0.000 | 0.002 | 0.165 | 1.000 |
| | micro | Longitude | 0.157 | 0.064 | 2.471 | 0.135 |
| | | Latitude | -0.110 | 0.064 | -1.719 | 0.856 |
| | | Elevation | 0.114 | 0.043 | 2.645 | 0.082 |
| $T_{min}$ Summer | macro | Longitude | 0.009 | 0.050 | 0.183 | 1.000 |
| | | Latitude | -0.128 | 0.053 | -2.403 | 0.163 |
| | | Elevation | 0.002 | 0.006 | 0.365 | 1.000 |
| | micro | Longitude | 0.107 | 0.052 | 2.064 | 0.390 |
| | | Latitude | -0.052 | 0.051 | -1.021 | 1.000 |
| | | Elevation | 0.016 | 0.051 | 0.308 | 1.000 |
| $T_{max}$ Summer | macro | Longitude | 0.013 | 0.070 | 0.180 | 1.000 |
| | | Latitude | -0.106 | 0.075 | -1.419 | 1.000 |
| | | Elevation | 0.005 | 0.003 | 1.389 | 1.000 |
| | micro | Longitude | 0.092 | 0.266 | 0.345 | 1.000 |
| | | Latitude | -0.477 | 0.267 | -1.786 | 0.741 |
| | | Elevation | 0.160 | 0.146 | 1.096 | 1.000 |
| $T_{mean}$ Winter | macro | Longitude | 0.117 | 0.054 | 2.179 | 0.294 |
| | | Latitude | 0.032 | 0.057 | 0.558 | 1.000 |
| | | Elevation | 0.004 | 0.003 | 1.636 | 1.000 |
| | micro | Longitude | 0.089 | 0.083 | 1.072 | 1.000 |
| | | Latitude | 0.101 | 0.082 | 1.224 | 1.000 |
| | | Elevation | 0.140 | 0.062 | 2.252 | 0.243 |
| $T_{min}$ Winter | macro | Longitude | 0.403 | 0.112 | 3.587 | **0.003** |
| | | Latitude | 0.119 | 0.117 | 1.013 | 1.000 |
| | | Elevation | -0.011 | 0.005 | -2.049 | 0.405 |
| | micro | Longitude | 0.217 | 0.118 | 1.835 | 0.665 |
| | | Latitude | 0.167 | 0.116 | 1.431 | 1.000 |
| | | Elevation | 0.194 | 0.101 | 1.920 | 0.549 |
| $T_{max}$ Winter | macro | Longitude | 0.046 | 0.031 | 1.476 | 1.000 |
| | | Latitude | 0.004 | 0.033 | 0.127 | 1.000 |
| | | Elevation | 0.007 | 0.004 | 1.686 | 0.918 |
| | micro | Longitude | 0.042 | 0.083 | 0.503 | 1.000 |
| | | Latitude | -0.039 | 0.082 | -0.479 | 1.000 |
| | | Elevation | 0.060 | 0.066 | 0.905 | 1.000 |
| $T_{mean}$ June | macro | Longitude | 0.285 | 0.061 | 4.668 | **< 0.001** |
| | | Latitude | 0.206 | 0.065 | 3.175 | **0.015** |
| | | Elevation | -0.009 | 0.005 | -1.972 | 0.486 |
| | micro | Longitude | 0.328 | 0.101 | 3.250 | **0.012** |
| | | Latitude | 0.069 | 0.100 | 0.694 | 1.000 |
| | | Elevation | 0.094 | 0.074 | 1.272 | 1.000 |
| $T_{min}$ June | macro | Longitude | 0.241 | 0.062 | 3.902 | **0.001** |
| | | Latitude | 0.165 | 0.066 | 2.503 | 0.123 |
| | | Elevation | -0.006 | 0.005 | -1.163 | 1.000 |
| | micro | Longitude | 0.289 | 0.057 | 5.099 | **< 0.001** |
| | | Latitude | 0.180 | 0.056 | 3.241 | **0.012** |
| | | Elevation | -0.012 | 0.057 | -0.202 | 1.000 |
| $T_{max}$ June | macro | Longitude | 0.337 | 0.066 | 5.106 | **< 0.001** |
| | | Latitude | 0.243 | 0.070 | 3.462 | **0.005** |
| | | Elevation | -0.011 | 0.006 | -1.898 | 0.576 |
| | micro | Longitude | 0.425 | 0.250 | 1.703 | 0.885 |
| | | Latitude | -0.134 | 0.249 | -0.536 | 1.000 |
| | | Elevation | 0.157 | 0.151 | 1.036 | 1.000 |

**Supplementary Table 6.** Mean ± standard deviation (minimum–maximum) values of each temperature metric when calculated with a time lag of 1–10 years compared to the monitoring period (i.e. for the periods 2000–2021 to 1991–2012). Bold values indicate that an intercept-only linear mixed-effects model with the rates of temperature change as response and a random-intercept term for mountain region, revealed that the respective rate of temperature change was significantly greater than zero, after adjusting the p values for multiple comparison.

| | **Macroclimatic temperature change** | | | | | | | | | |
|---|---|---|---|---|---|---|---|---|---|---|
| | **1-year lag** | **2-year lag** | **3-year lag** | **4-year lag** | **5-year lag** | **6-year lag** | **7-year lag** | **8-year lag** | **9-year lag** | **10-year lag** |
| $T_{mean}$ Annual | **0.38 ± 0.15 (0.03–0.64)** | **0.41 ± 0.17 (0.04–0.68)** | **0.39 ± 0.18 (0.04–0.72)** | **0.36 ± 0.19 (-0.02–0.73)** | **0.4 ± 0.19 (0.07–0.76)** | **0.41 ± 0.21 (0.06–0.79)** | **0.34 ± 0.17 (0.07–0.65)** | **0.36 ± 0.14 (0.13–0.63)** | **0.28 ± 0.14 (0.1–0.59)** | **0.36 ± 0.11 (0.21–0.58)** |
| $T_{mean}$ Summer | **0.33 ± 0.13 (0.06–0.65)** | **0.31 ± 0.16 (0.02–0.61)** | **0.35 ± 0.17 (0.08–0.69)** | **0.39 ± 0.19 (0.07–0.79)** | **0.42 ± 0.18 (0.09–0.79)** | **0.44 ± 0.19 (0.03–0.81)** | **0.37 ± 0.15 (0.06–0.68)** | **0.38 ± 0.13 (0.23–0.63)** | **0.41 ± 0.13 (0.23–0.67)** | **0.49 ± 0.14 (0.24–0.74)** |
| $T_{min}$ Summer | -0.14 ± 0.36 (-0.5–1) | -0.11 ± 0.35 (-0.55–1.04) | -0.06 ± 0.4 (-0.56–1.02) | 0.13 ± 0.3 (-0.36–0.85) | 0.08 ± 0.27 (-0.29–0.63) | 0.1 ± 0.26 (-0.29–0.73) | 0.18 ± 0.24 (-0.23–0.79) | 0.15 ± 0.31 (-0.26–0.85) | 0.18 ± 0.29 (-0.23–0.68) | **0.51 ± 0.14 (0.31–0.84)** |
| $T_{max}$ Summer | **0.46 ± 0.2 (0.05–0.83)** | **0.51 ± 0.26 (-0.07–0.96)** | **0.62 ± 0.29 (-0.14–0.97)** | **0.69 ± 0.33 (-0.01–1.22)** | **0.75 ± 0.4 (-0.08–1.26)** | **0.62 ± 0.3 (-0.08–0.92)** | **0.49 ± 0.25 (-0.03–0.8)** | **0.47 ± 0.18 (0.18–0.87)** | **0.44 ± 0.18 (0.03–0.81)** | **0.38 ± 0.28 (-0.14–1.05)** |
| $T_{mean}$ Winter | **0.53 ± 0.24 (0.04–0.89)** | **0.55 ± 0.23 (0.08–0.92)** | **0.44 ± 0.26 (-0.05–0.9)** | **0.28 ± 0.24 (-0.17–0.68)** | **0.38 ± 0.3 (0.02–0.91)** | **0.4 ± 0.32 (-0.1–0.94)** | **0.25 ± 0.28 (-0.24–0.68)** | **0.24 ± 0.26 (-0.23–0.65)** | 0.04 ± 0.28 (-0.32–0.67) | 0.11 ± 0.22 (-0.27–0.59) |
| $T_{min}$ Winter | **0.87 ± 0.55 (-0.22–1.86)** | **0.7 ± 0.38 (-0.05–1.31)** | **0.49 ± 0.45 (-0.22–1.37)** | 0.3 ± 0.45 (-0.53–1.11) | 0.31 ± 0.41 (-0.38–0.98) | **0.48 ± 0.52 (-0.51–1.29)** | 0.28 ± 0.41 (-0.51–0.96) | 0.19 ± 0.37 (-0.49–0.68) | -0.2 ± 0.39 (-0.93–0.71) | -0.14 ± 0.37 (-0.8–0.59) |
| $T_{max}$ Winter | **0.34 ± 0.18 (-0.02–0.54)** | **0.44 ± 0.23 (0.05–0.73)** | **0.48 ± 0.34 (-0.08–1.07)** | **0.32 ± 0.41 (-0.46–0.95)** | **0.44 ± 0.36 (-0.2–1.03)** | **0.43 ± 0.38 (-0.38–1)** | **0.4 ± 0.4 (-0.36–1)** | **0.55 ± 0.51 (-0.35–1.48)** | **0.4 ± 0.47 (-0.38–1.29)** | **0.46 ± 0.38 (-0.14–1.15)** |
| $T_{mean}$ June | **0.39 ± 0.42 (-0.29–1.21)** | **0.35 ± 0.35 (-0.2–1.08)** | **0.41 ± 0.28 (-0.1–0.81)** | **0.36 ± 0.2 (-0.08–0.68)** | **0.35 ± 0.16 (-0.04–0.66)** | **0.34 ± 0.2 (-0.21–0.67)** | **0.4 ± 0.23 (-0.18–0.88)** | **0.42 ± 0.21 (0–0.84)** | **0.51 ± 0.19 (0.13–0.98)** | **0.73 ± 0.21 (0.35–1.05)** |
| $T_{min}$ June | **0.41 ± 0.39 (-0.22–1)** | **0.36 ± 0.34 (-0.21–0.88)** | **0.37 ± 0.28 (-0.14–0.75)** | **0.32 ± 0.21 (-0.05–0.72)** | **0.32 ± 0.19 (0.01–0.6)** | **0.3 ± 0.23 (-0.1–0.6)** | **0.34 ± 0.24 (-0.13–0.65)** | **0.36 ± 0.2 (-0.11–0.59)** | **0.45 ± 0.15 (0.18–0.71)** | **0.64 ± 0.17 (0.37–1.01)** |
| $T_{max}$ June | **0.38 ± 0.45 (-0.39–1.44)** | **0.33 ± 0.39 (-0.23–1.32)** | **0.46 ± 0.31 (-0.06–0.95)** | **0.41 ± 0.22 (-0.12–0.71)** | **0.39 ± 0.18 (-0.1–0.85)** | **0.39 ± 0.23 (-0.35–0.84)** | **0.46 ± 0.28 (-0.36–1.11)** | **0.48 ± 0.27 (-0.19–1.1)** | **0.57 ± 0.28 (0.06–1.27)** | **0.82 ± 0.28 (0.22–1.23)** |

**Supplementary Table 7.** Summaries of linear mixed-effects models relating plot-level thermophilization rate separately to the rates of changes in each of the ten temperature metrics on both climatic scales. The models included a random-intercept term for plot cluster nested in summit nested in mountain region. Scale indicates which version of the temperature metric was used (macro- or microclimatic). Only the one-sided, right-tailed p values adjusted for multiple comparisons are reported (this adjustment can lead to p values of exactly 1). Bold p values indicate statistical significance. The reported estimated effects are based on models fitted with the rates of temperature change scaled to zero mean and unit variance for better comparability. Est. ... estimated effect; S.E. ... standard error; $R^2m$ ... marginal $R^2$; $T_{mean}$ ... mean temperature; $T_{min}$ ... minimum temperature; $T_{max}$ ... maximum temperature.

| Predictor | Scale | Est. | S.E. | z value | p value | $R^2m$ |
|---|---|---|---|---|---|---|
| $T_{mean}$ Annual | macro | 0.028 | 0.015 | 1.838 | 0.135 | 0.019 |
| | micro | -0.018 | 0.011 | -1.599 | 1.000 | 0.008 |
| $T_{mean}$ Summer | macro | 0.015 | 0.016 | 0.919 | 1.000 | 0.005 |
| | micro | -0.020 | 0.010 | -1.858 | 1.000 | 0.009 |
| $T_{min}$ Summer | macro | -0.030 | 0.015 | -2.019 | 1.000 | 0.022 |
| | micro | -0.018 | 0.010 | -1.914 | 1.000 | 0.008 |
| $T_{max}$ Summer | macro | 0.022 | 0.015 | 1.517 | 0.433 | 0.012 |
| | micro | 0.001 | 0.011 | 0.132 | 1.000 | 0.000 |
| $T_{mean}$ Winter | macro | 0.035 | 0.014 | 2.469 | **0.025** | 0.030 |
| | micro | -0.011 | 0.011 | -1.050 | 1.000 | 0.003 |
| $T_{min}$ Winter | macro | 0.036 | 0.014 | 2.557 | **0.020** | 0.043 |
| | micro | -0.011 | 0.010 | -1.082 | 1.000 | 0.003 |
| $T_{max}$ Winter | macro | 0.025 | 0.016 | 1.561 | 0.400 | 0.015 |
| | micro | 0.009 | 0.010 | 0.873 | 1.000 | 0.002 |
| $T_{mean}$ June | macro | 0.029 | 0.015 | 1.950 | 0.104 | 0.020 |
| | micro | -0.011 | 0.011 | -0.961 | 1.000 | 0.003 |
| $T_{min}$ June | macro | 0.025 | 0.015 | 1.634 | 0.205 | 0.015 |
| | micro | -0.024 | 0.010 | -2.283 | 1.000 | 0.014 |
| $T_{max}$ June | macro | 0.030 | 0.015 | 2.071 | 0.082 | 0.022 |
| | micro | 0.006 | 0.011 | 0.549 | 1.000 | 0.001 |

**Supplementary Table 8.** Summaries of linear mixed-effects models, each relating plot-level thermophilization rate to two macroclimatic temperature metrics calculated for the monitoring period, accounting for interactions between the two. Only the one-sided, right-tailed p values are reported for the main effects, while the two-sided p values are reported for the interaction effects. P values were further adjusted for multiple comparisons (this adjustment can lead to p values of exactly 1). Bold p values indicate statistical significance. The reported estimated effects are based on models fitted with the rates of temperature change scaled to zero mean and unit variance for better comparability. Est. ... estimated effect; S.E. ... standard error; $R^2$m ... marginal $R^2$; $T_{mean}$ ... mean temperature; $T_{min}$ ... minimum temperature; $T_{max}$ ... maximum temperature; Interaction ... interaction between two temperature metrics.

| Predictor | Est. | S.E. | z value | p value | $R^2$m |
|---|---|---|---|---|---|
| $T_{min}$ Summer | -0.032 | 0.013 | -2.429 | 0.272 | 0.079 |
| $T_{mean}$ Annual | 0.021 | 0.013 | 1.548 | 1.000 | |
| Interaction | -0.032 | 0.016 | -2.031 | 0.567 | |
| $T_{min}$ Summer | -0.028 | 0.014 | -1.974 | 0.870 | 0.060 |
| $T_{mean}$ Summer | 0.015 | 0.014 | 1.078 | 1.000 | |
| Interaction | -0.029 | 0.019 | -1.492 | 1.000 | |
| $T_{max}$ Summer | 0.021 | 0.013 | 1.680 | 1.000 | 0.073 |
| $T_{min}$ Summer | -0.028 | 0.013 | -2.120 | 0.610 | |
| Interaction | -0.029 | 0.015 | -1.905 | 0.951 | |
| $T_{mean}$ Winter | 0.020 | 0.014 | 1.409 | 1.000 | 0.077 |
| $T_{min}$ Summer | -0.032 | 0.014 | -2.203 | 0.496 | |
| Interaction | -0.029 | 0.016 | -1.884 | 0.771 | |
| $T_{mean}$ Winter | 0.030 | 0.016 | 1.894 | 0.461 | 0.056 |
| $T_{max}$ Summer | 0.013 | 0.015 | 0.878 | 1.000 | |
| Interaction | 0.023 | 0.017 | 1.337 | 1.000 | |
| $T_{min}$ Winter | 0.034 | 0.015 | 2.283 | 0.180 | 0.044 |
| $T_{mean}$ Summer | 0.007 | 0.018 | 0.356 | 1.000 | |
| Interaction | 0.008 | 0.019 | 0.401 | 1.000 | |
| $T_{min}$ Winter | 0.027 | 0.014 | 1.890 | 0.471 | 0.078 |
| $T_{min}$ Summer | -0.026 | 0.015 | -1.806 | 1.000 | |
| Interaction | -0.025 | 0.012 | -2.097 | 0.468 | |
| $T_{min}$ Winter | 0.026 | 0.017 | 1.573 | 0.927 | 0.051 |
| $T_{max}$ Summer | 0.016 | 0.015 | 1.097 | 1.000 | |
| Interaction | 0.011 | 0.017 | 0.611 | 1.000 | |
| $T_{max}$ Winter | 0.033 | 0.015 | 2.172 | 0.469 | 0.093 |
| $T_{mean}$ Annual | 0.032 | 0.015 | 2.220 | 0.235 | |
| Interaction | 0.040 | 0.012 | 3.426 | **0.008** | |
| $T_{max}$ Winter | 0.040 | 0.015 | 2.706 | 0.107 | 0.065 |
| $T_{mean}$ Summer | 0.021 | 0.013 | 1.562 | 1.000 | |
| Interaction | 0.036 | 0.014 | 2.655 | 0.119 | |
| $T_{max}$ Winter | 0.020 | 0.014 | 1.425 | 1.000 | 0.046 |
| $T_{min}$ Summer | -0.029 | 0.015 | -1.992 | 0.832 | |
| Interaction | -0.007 | 0.013 | -0.537 | 1.000 | |
| $T_{max}$ Winter | 0.032 | 0.014 | 2.362 | 0.286 | 0.070 |
| $T_{max}$ Summer | 0.009 | 0.014 | 0.612 | 1.000 | |
| Interaction | 0.032 | 0.014 | 2.249 | 0.383 | |

**Supplementary Table 8.** Continued.

| Predictor | Est. | S.E. | z value | p value | $R^2$m |
|---|---|---|---|---|---|
| $T_{mean}$ June | 0.027 | 0.016 | 1.725 | 0.743 | 0.022 |
| $T_{mean}$ Summer | 0.007 | 0.016 | 0.425 | 1.000 | |
| Interaction | -0.003 | 0.022 | -0.129 | 1.000 | |
| $T_{mean}$ June | 0.034 | 0.015 | 2.242 | 0.220 | 0.077 |
| $T_{min}$ Summer | -0.026 | 0.014 | -1.797 | 1.000 | |
| Interaction | -0.028 | 0.011 | -2.504 | 0.164 | |
| $T_{mean}$ June | 0.019 | 0.015 | 1.258 | 1.000 | 0.043 |
| $T_{max}$ Summer | 0.020 | 0.014 | 1.390 | 1.000 | |
| Interaction | 0.014 | 0.017 | 0.826 | 1.000 | |
| $T_{mean}$ June | 0.014 | 0.014 | 0.970 | 1.000 | 0.084 |
| $T_{max}$ Winter | 0.035 | 0.016 | 2.237 | 0.398 | |
| Interaction | 0.040 | 0.013 | 2.941 | **0.040** | |
| $T_{min}$ June | 0.022 | 0.016 | 1.370 | 1.000 | 0.017 |
| $T_{mean}$ Summer | 0.010 | 0.016 | 0.590 | 1.000 | |
| Interaction | 0.007 | 0.017 | 0.408 | 1.000 | |
| $T_{min}$ June | 0.026 | 0.015 | 1.683 | 0.804 | 0.065 |
| $T_{min}$ Summer | -0.030 | 0.015 | -2.020 | 0.778 | |
| Interaction | -0.028 | 0.013 | -2.157 | 0.414 | |
| $T_{min}$ June | 0.017 | 0.015 | 1.164 | 1.000 | 0.041 |
| $T_{max}$ Summer | 0.021 | 0.014 | 1.536 | 1.000 | |
| Interaction | 0.016 | 0.016 | 1.025 | 1.000 | |
| $T_{min}$ June | 0.016 | 0.015 | 1.115 | 1.000 | 0.079 |
| $T_{max}$ Winter | 0.038 | 0.016 | 2.295 | 0.341 | |
| Interaction | 0.041 | 0.014 | 2.956 | **0.038** | |
| $T_{max}$ June | 0.033 | 0.015 | 2.151 | 0.296 | 0.038 |
| $T_{mean}$ Summer | 0.003 | 0.015 | 0.223 | 1.000 | |
| Interaction | -0.020 | 0.025 | -0.801 | 1.000 | |
| $T_{max}$ June | 0.037 | 0.015 | 2.460 | 0.131 | 0.082 |
| $T_{min}$ Summer | -0.024 | 0.014 | -1.718 | 1.000 | |
| Interaction | -0.028 | 0.011 | -2.617 | 0.121 | |
| $T_{max}$ June | 0.021 | 0.016 | 1.254 | 1.000 | 0.043 |
| $T_{max}$ Summer | 0.019 | 0.015 | 1.269 | 1.000 | |
| Interaction | 0.011 | 0.018 | 0.635 | 1.000 | |
| $T_{max}$ June | 0.014 | 0.014 | 0.982 | 1.000 | 0.085 |
| $T_{max}$ Winter | 0.032 | 0.015 | 2.121 | 0.533 | |
| Interaction | 0.038 | 0.013 | 2.870 | 0.052 | |

**Supplementary Table 9.** Summaries of linear mixed-effects models, each relating plot-level thermophilization rate to two macroclimatic temperature metrics calculated with a 4-year time lag, accounting for interactions between the two. Only the one-sided, right-tailed p values adjusted for multiple comparisons are reported (this adjustment can lead to p values of exactly 1). Bold p values indicate statistical significance. The reported estimated effects are based on models fitted with the rates of temperature change scaled to zero mean and unit variance for better comparability. Est. ... estimated effect; S.E. ... standard error; $R^2m$ ... marginal $R^2$; $T_{mean}$ ... mean temperature; $T_{min}$ ... minimum temperature; $T_{max}$ ... maximum temperature; Interaction ... interaction between two temperature metrics.

| Predictor | Est. | S.E. | z value | p value | $R^2m$ |
|---|---|---|---|---|---|
| $T_{min}$ Summer | -0.003 | 0.014 | -0.238 | 1.000 | 0.087 |
| $T_{mean}$ Annual | 0.029 | 0.017 | 1.677 | 0.676 | |
| Interaction | 0.034 | 0.018 | 1.865 | 0.686 | |
| $T_{min}$ Summer | -0.010 | 0.015 | -0.684 | 1.000 | 0.069 |
| $T_{mean}$ Summer | 0.027 | 0.020 | 1.389 | 1.000 | |
| Interaction | 0.029 | 0.019 | 1.481 | 1.000 | |
| $T_{max}$ Summer | 0.005 | 0.017 | 0.291 | 1.000 | 0.091 |
| $T_{min}$ Summer | 0.013 | 0.013 | 0.990 | 1.000 | |
| Interaction | 0.057 | 0.020 | 2.829 | 0.055 | |
| $T_{mean}$ Winter | 0.035 | 0.016 | 2.104 | 0.283 | 0.069 |
| $T_{min}$ Summer | -0.008 | 0.014 | -0.543 | 1.000 | |
| Interaction | 0.021 | 0.017 | 1.230 | 1.000 | |
| $T_{min}$ Winter | 0.029 | 0.015 | 1.909 | 0.561 | 0.074 |
| $T_{min}$ Summer | 0.002 | 0.015 | 0.139 | 1.000 | |
| Interaction | 0.024 | 0.014 | 1.737 | 1.000 | |
| $T_{min}$ Winter | 0.031 | 0.015 | 2.081 | 0.374 | 0.078 |
| $T_{max}$ Summer | 0.022 | 0.015 | 1.435 | 1.000 | |
| Interaction | 0.010 | 0.012 | 0.814 | 1.000 | |
| $T_{max}$ Winter | 0.049 | 0.015 | 3.349 | **0.007** | 0.081 |
| $T_{min}$ Summer | -0.021 | 0.014 | -1.441 | 1.000 | |
| Interaction | 0.033 | 0.017 | 1.972 | 0.563 | |
| $T_{mean}$ June | 0.020 | 0.015 | 1.355 | 1.000 | 0.077 |
| $T_{mean}$ Annual | 0.033 | 0.020 | 1.655 | 0.708 | |
| Interaction | 0.006 | 0.021 | 0.272 | 1.000 | |
| $T_{mean}$ June | 0.018 | 0.014 | 1.327 | 1.000 | 0.082 |
| $T_{min}$ Summer | 0.002 | 0.013 | 0.121 | 1.000 | |
| Interaction | 0.047 | 0.017 | 2.869 | 0.050 | |
| $T_{mean}$ June | 0.023 | 0.014 | 1.655 | 0.951 | 0.075 |
| $T_{mean}$ Winter | 0.029 | 0.015 | 1.944 | 0.416 | |
| Interaction | 0.010 | 0.016 | 0.637 | 1.000 | |
| $T_{mean}$ June | 0.026 | 0.014 | 1.886 | 0.576 | 0.082 |
| $T_{min}$ Winter | 0.035 | 0.013 | 2.658 | 0.078 | |
| Interaction | 0.006 | 0.013 | 0.462 | 1.000 | |
| $T_{min}$ June | 0.027 | 0.013 | 2.091 | 0.321 | 0.085 |
| $T_{min}$ Summer | 0.011 | 0.013 | 0.864 | 1.000 | |
| Interaction | 0.038 | 0.013 | 2.816 | 0.057 | |

**Supplementary Table 9.** Continued.

| Predictor | Est. | S.E. | z value | p value | $R^2m$ |
|---|---|---|---|---|---|
| $T_{min}$ June | 0.026 | 0.014 | 1.824 | 0.599 | 0.081 |
| $T_{min}$ Winter | 0.032 | 0.014 | 2.273 | 0.230 | |
| Interaction | 0.016 | 0.013 | 1.267 | 1.000 | |
| $T_{max}$ June | 0.016 | 0.014 | 1.160 | 1.000 | 0.076 |
| $T_{mean}$ Annual | 0.049 | 0.023 | 2.170 | 0.217 | |
| Interaction | -0.011 | 0.022 | -0.482 | 1.000 | |
| $T_{max}$ June | 0.024 | 0.018 | 1.378 | 1.000 | 0.063 |
| $T_{mean}$ Summer | 0.013 | 0.023 | 0.581 | 1.000 | |
| Interaction | 0.022 | 0.020 | 1.114 | 1.000 | |
| $T_{max}$ June | 0.015 | 0.015 | 0.974 | 1.000 | 0.043 |
| $T_{min}$ Summer | -0.009 | 0.014 | -0.659 | 1.000 | |
| Interaction | 0.046 | 0.020 | 2.370 | 0.239 | |
| $T_{max}$ June | 0.022 | 0.013 | 1.621 | 1.000 | 0.073 |
| $T_{mean}$ Winter | 0.039 | 0.017 | 2.255 | 0.193 | |
| Interaction | 0.000 | 0.019 | -0.024 | 1.000 | |
| $T_{max}$ June | 0.029 | 0.016 | 1.898 | 0.694 | 0.082 |
| $T_{min}$ Winter | 0.043 | 0.014 | 3.009 | **0.026** | |
| Interaction | -0.003 | 0.016 | -0.197 | 1.000 | |
| $T_{max}$ June | 0.019 | 0.014 | 1.364 | 1.000 | 0.073 |
| $T_{max}$ Winter | 0.028 | 0.014 | 1.924 | 0.455 | |
| Interaction | 0.025 | 0.014 | 1.747 | 0.823 | |

**Supplementary Table 10.** Summaries of linear mixed-effects models, each relating plot-level thermophilization rate to two microclimatic temperature metrics, accounting for interactions between the two. Only the one-sided, right-tailed p values adjusted for multiple comparisons are reported (this adjustment can lead to p values of exactly 1). The reported estimated effects are based on models fitted with the rates of temperature change scaled to zero mean and unit variance for better comparability. Est. ... estimated effect; S.E. ... standard error; $R^2m$ ... marginal $R^2$; $T_{mean}$ ... mean temperature; $T_{min}$ ... minimum temperature; $T_{max}$ ... maximum temperature; Interaction ... interaction between two temperature metrics.

| Predictor | Est. | S.E. | z value | p value | $R^2m$ |
|---|---|---|---|---|---|
| $T_{min}$ Summer | -0.015 | 0.010 | -1.403 | 1.000 | 0.013 |
| $T_{mean}$ Annual | -0.005 | 0.013 | -0.353 | 1.000 | |
| Interaction | 0.009 | 0.008 | 1.089 | 1.000 | |
| $T_{min}$ Summer | -0.014 | 0.011 | -1.241 | 1.000 | 0.012 |
| $T_{mean}$ Summer | -0.012 | 0.012 | -0.997 | 1.000 | |
| Interaction | 0.003 | 0.007 | 0.410 | 1.000 | |
| $T_{max}$ Summer | 0.012 | 0.012 | 1.006 | 1.000 | 0.016 |
| $T_{mean}$ Annual | -0.019 | 0.011 | -1.651 | 1.000 | |
| Interaction | 0.012 | 0.007 | 1.775 | 1.000 | |
| $T_{max}$ Summer | -0.002 | 0.012 | -0.146 | 1.000 | 0.009 |
| $T_{min}$ Summer | -0.019 | 0.010 | -1.923 | 1.000 | |
| Interaction | 0.001 | 0.008 | 0.123 | 1.000 | |
| $T_{mean}$ Winter | -0.008 | 0.011 | -0.780 | 1.000 | 0.012 |
| $T_{mean}$ Summer | -0.011 | 0.013 | -0.917 | 1.000 | |
| Interaction | 0.006 | 0.006 | 1.013 | 1.000 | |

**Supplementary Table 10.** Continued.

| Predictor | Est. | S.E. | z value | p value | $R^2m$ |
|---|---|---|---|---|---|
| $T_{mean}$ Winter | -0.005 | 0.011 | -0.399 | 1.000 | 0.012 |
| $T_{min}$ Summer | -0.013 | 0.010 | -1.226 | 1.000 | |
| Interaction | 0.010 | 0.008 | 1.214 | 1.000 | |
| $T_{mean}$ Winter | -0.011 | 0.011 | -0.992 | 1.000 | 0.005 |
| $T_{max}$ Summer | 0.006 | 0.012 | 0.480 | 1.000 | |
| Interaction | 0.008 | 0.008 | 0.974 | 1.000 | |
| $T_{min}$ Winter | -0.012 | 0.010 | -1.224 | 1.000 | 0.014 |
| $T_{mean}$ Summer | -0.014 | 0.012 | -1.174 | 1.000 | |
| Interaction | 0.007 | 0.008 | 0.851 | 1.000 | |
| $T_{min}$ Winter | -0.009 | 0.010 | -0.906 | 1.000 | 0.011 |
| $T_{min}$ Summer | -0.015 | 0.011 | -1.422 | 1.000 | |
| Interaction | 0.005 | 0.009 | 0.587 | 1.000 | |
| $T_{min}$ Winter | -0.011 | 0.010 | -1.036 | 1.000 | 0.004 |
| $T_{max}$ Summer | 0.004 | 0.012 | 0.285 | 1.000 | |
| Interaction | 0.006 | 0.008 | 0.776 | 1.000 | |
| $T_{max}$ Winter | 0.020 | 0.011 | 1.885 | 1.000 | 0.018 |
| $T_{mean}$ Annual | -0.021 | 0.013 | -1.680 | 1.000 | |
| Interaction | 0.007 | 0.004 | 1.636 | 1.000 | |
| $T_{max}$ Winter | 0.015 | 0.010 | 1.454 | 1.000 | 0.021 |
| $T_{mean}$ Summer | -0.014 | 0.012 | -1.199 | 1.000 | |
| Interaction | 0.012 | 0.006 | 2.076 | 0.828 | |
| $T_{max}$ Winter | 0.014 | 0.010 | 1.333 | 1.000 | 0.017 |
| $T_{min}$ Summer | -0.018 | 0.009 | -1.880 | 1.000 | |
| Interaction | 0.012 | 0.007 | 1.871 | 1.000 | |
| $T_{max}$ Winter | 0.002 | 0.012 | 0.157 | 1.000 | 0.010 |
| $T_{max}$ Summer | -0.009 | 0.013 | -0.713 | 1.000 | |
| Interaction | 0.012 | 0.007 | 1.847 | 1.000 | |
| $T_{max}$ Winter | 0.018 | 0.011 | 1.683 | 1.000 | 0.012 |
| $T_{mean}$ Winter | -0.018 | 0.011 | -1.527 | 1.000 | |
| Interaction | 0.008 | 0.005 | 1.527 | 1.000 | |
| $T_{max}$ Winter | 0.014 | 0.011 | 1.295 | 1.000 | 0.007 |
| $T_{min}$ Winter | -0.014 | 0.011 | -1.350 | 1.000 | |
| Interaction | 0.006 | 0.006 | 1.003 | 1.000 | |
| $T_{mean}$ June | -0.007 | 0.012 | -0.583 | 1.000 | 0.009 |
| $T_{min}$ Summer | -0.016 | 0.010 | -1.600 | 1.000 | |
| Interaction | -0.004 | 0.008 | -0.477 | 1.000 | |
| $T_{mean}$ June | -0.004 | 0.012 | -0.371 | 1.000 | 0.009 |
| $T_{mean}$ Winter | -0.011 | 0.011 | -1.046 | 1.000 | |
| Interaction | 0.011 | 0.007 | 1.511 | 1.000 | |
| $T_{mean}$ June | -0.008 | 0.012 | -0.714 | 1.000 | 0.009 |
| $T_{min}$ Winter | -0.014 | 0.010 | -1.333 | 1.000 | |
| Interaction | 0.011 | 0.008 | 1.286 | 1.000 | |

**Supplementary Table 10.** Continued.

| Predictor | Est. | S.E. | z value | p value | $R^2m$ |
|---|---|---|---|---|---|
| $T_{mean}$ June | -0.002 | 0.012 | -0.185 | 1.000 | 0.016 |
| $T_{max}$ Winter | 0.010 | 0.010 | 0.964 | 1.000 | |
| Interaction | 0.019 | 0.008 | 2.406 | 0.362 | |
| $T_{min}$ June | -0.019 | 0.012 | -1.677 | 1.000 | 0.015 |
| $T_{mean}$ Annual | -0.007 | 0.013 | -0.501 | 1.000 | |
| Interaction | 0.002 | 0.006 | 0.356 | 1.000 | |
| $T_{min}$ June | -0.019 | 0.011 | -1.627 | 1.000 | 0.016 |
| $T_{mean}$ Summer | -0.012 | 0.012 | -0.993 | 1.000 | |
| Interaction | -0.001 | 0.006 | -0.177 | 1.000 | |
| $T_{min}$ June | -0.020 | 0.013 | -1.523 | 1.000 | 0.014 |
| $T_{min}$ Summer | -0.008 | 0.012 | -0.719 | 1.000 | |
| Interaction | -0.003 | 0.007 | -0.454 | 1.000 | |
| $T_{min}$ June | -0.024 | 0.011 | -2.237 | 0.572 | 0.016 |
| $T_{max}$ Summer | -0.008 | 0.013 | -0.650 | 1.000 | |
| Interaction | -0.003 | 0.007 | -0.416 | 1.000 | |
| $T_{min}$ June | -0.019 | 0.011 | -1.662 | 1.000 | 0.015 |
| $T_{mean}$ Winter | -0.006 | 0.011 | -0.544 | 1.000 | |
| Interaction | 0.005 | 0.006 | 0.722 | 1.000 | |
| $T_{min}$ June | -0.020 | 0.011 | -1.801 | 1.000 | 0.016 |
| $T_{min}$ Winter | -0.008 | 0.010 | -0.732 | 1.000 | |
| Interaction | 0.003 | 0.007 | 0.486 | 1.000 | |
| $T_{min}$ June | -0.021 | 0.010 | -1.975 | 1.000 | 0.016 |
| $T_{max}$ Winter | 0.013 | 0.011 | 1.164 | 1.000 | |
| Interaction | 0.007 | 0.006 | 1.203 | 1.000 | |
| $T_{max}$ June | 0.015 | 0.012 | 1.216 | 1.000 | 0.012 |
| $T_{mean}$ Annual | -0.021 | 0.011 | -1.878 | 1.000 | |
| Interaction | 0.007 | 0.008 | 0.965 | 1.000 | |
| $T_{max}$ June | 0.002 | 0.012 | 0.161 | 1.000 | 0.009 |
| $T_{min}$ Summer | -0.017 | 0.010 | -1.741 | 1.000 | |
| Interaction | -0.002 | 0.008 | -0.274 | 1.000 | |
| $T_{max}$ June | 0.009 | 0.013 | 0.732 | 1.000 | 0.005 |
| $T_{mean}$ Winter | -0.012 | 0.011 | -1.055 | 1.000 | |
| Interaction | 0.007 | 0.008 | 0.839 | 1.000 | |
| $T_{max}$ June | 0.006 | 0.013 | 0.495 | 1.000 | 0.004 |
| $T_{min}$ Winter | -0.011 | 0.011 | -1.028 | 1.000 | |
| Interaction | 0.004 | 0.008 | 0.489 | 1.000 | |
| $T_{max}$ June | -0.001 | 0.012 | -0.113 | 1.000 | 0.009 |
| $T_{max}$ Winter | -0.001 | 0.012 | -0.109 | 1.000 | |
| Interaction | 0.013 | 0.007 | 1.781 | 1.000 | |
| $T_{max}$ June | 0.003 | 0.012 | 0.262 | 1.000 | 0.014 |
| $T_{min}$ June | -0.022 | 0.011 | -1.948 | 1.000 | |
| Interaction | -0.002 | 0.007 | -0.264 | 1.000 | |

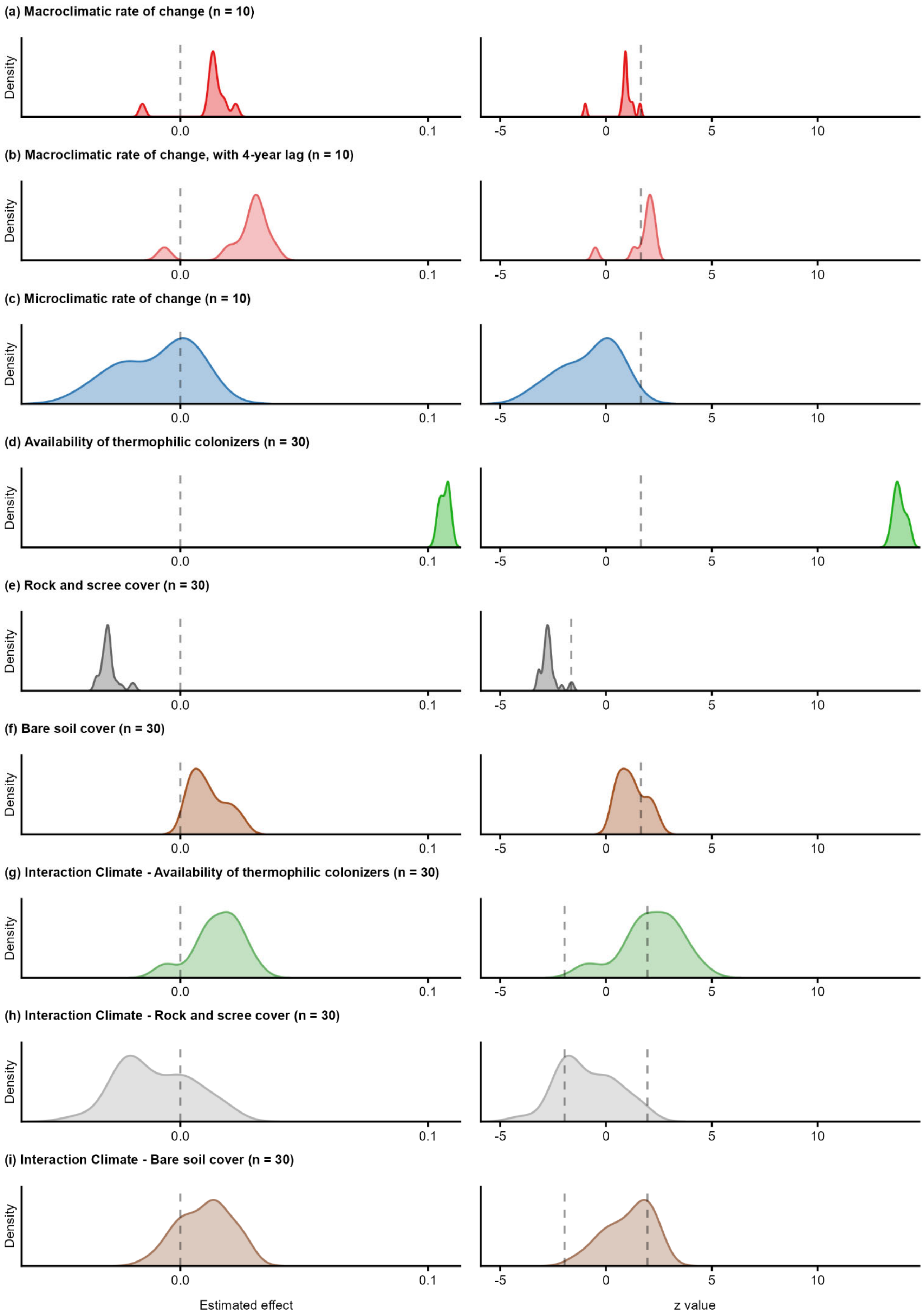

(a) Macroclimatic rate of change (n = 10)
(b) Macroclimatic rate of change, with 4-year lag (n = 10)
(c) Microclimatic rate of change (n = 10)
(d) Availability of thermophilic colonizers (n = 30)
(e) Rock and scree cover (n = 30)
(f) Bare soil cover (n = 30)
(g) Interaction Climate - Availability of thermophilic colonizers (n = 30)
(h) Interaction Climate - Rock and scree cover (n = 30)
(i) Interaction Climate - Bare soil cover (n = 30)
Density
0.0
0.1
-5
0
5
10
Estimated effect
z value

**Supplementary Fig. 2. Results of linear mixed-effects models relating thermophilization rates to rates of temperature change and non-climatic covariables, including interactions between rates of temperature change and these covariables.** Density distributions of the estimated effects (left) and z values (right) of the different predictors (a–f) and the interactions between temperature change and the non-climatic covariables (g–i) as derived from the 30 fitted linear mixed-effects models, one for each of the ten temperature metrics on both climatic scales and including macroclimatic temperature change calculated with a 4-year time lag, with thermophilization rate as response variable. For macroclimatic (a-b) and microclimatic rates of temperature change (c) these distributions incorporate the estimated effects and z values across all ten temperature metrics. Dashed lines indicate 0 for the estimated effects of the predictors (left) and the critical z values for one-sided tests (right; with the expectation of positive relationships for all but rock and scree cover, for which a negative relationship was tested). The sample sizes (n; i.e. the number of models fitted that included the individual predictors) are given in parentheses after the predictor names. Partial $R^2m$ values are not provided as a clear attribution of the explained variance to one single predictor is difficult with the presence of interactions between the predictors. The mean ± SD $R^2m$ value across the 30 full models was 0.38 ± 0.07.

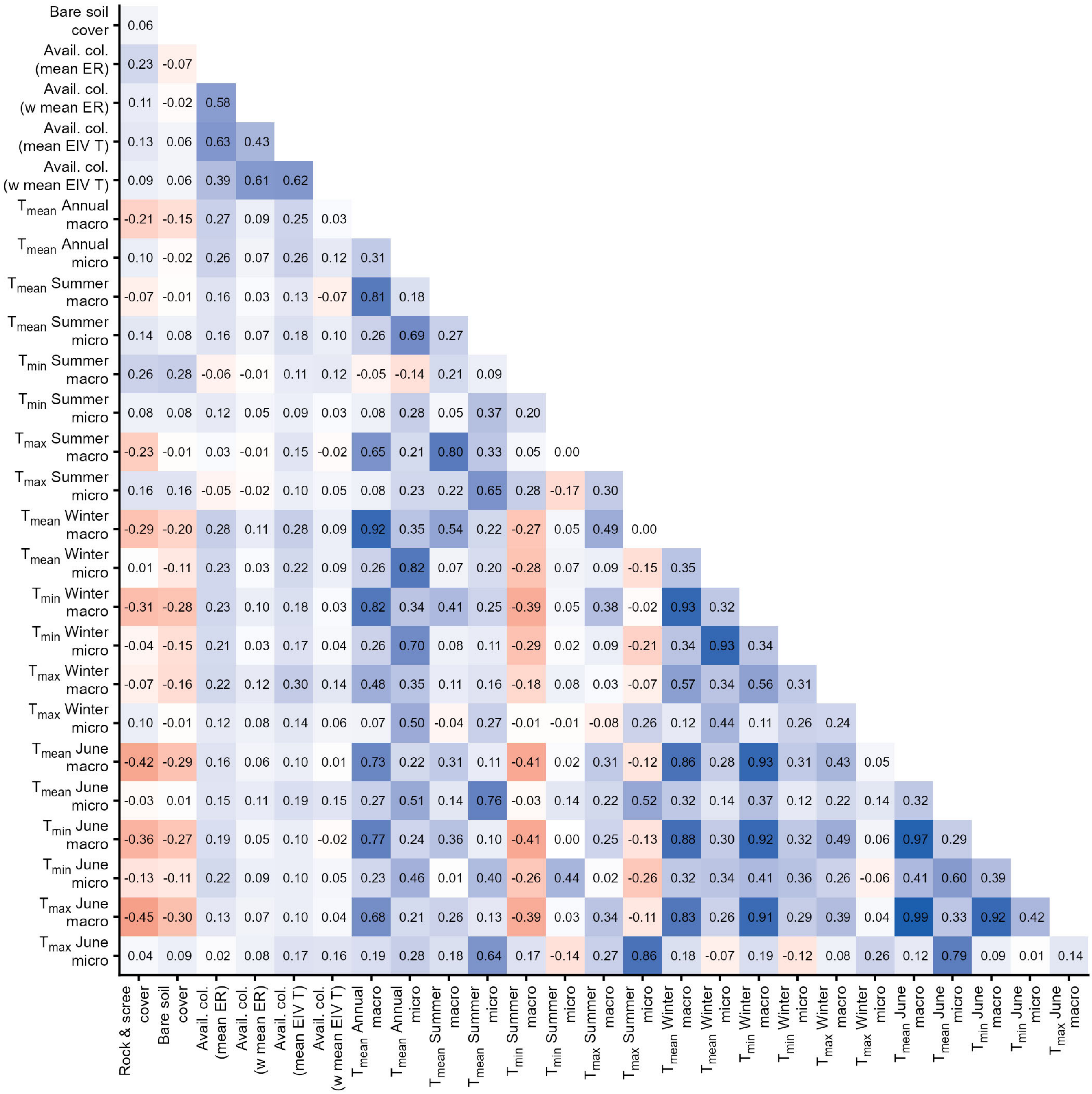


**Supplementary Fig. 3. Correlations between predictor variables.** Pearson correlations coefficients of correlations between the different predictor variables used across any of the fitted models. All four versions of the propagule pressure variables calculated for the four different types of thermophilization rates are shown, i.e. thermophilization rates expressed as plot-level changes in the cover-weighted mean elevational rank (w mean ER) as presented in the main text, or as plot-level changes in either the mean elevational rank (mean ER), the cover-weighted mean EIV T (w mean EIV T), or the mean EIV T (results presented in the supporting information). Avail. col. … Availability of thermophilic colonizers; $T_{mean}$ … mean temperature; $T_{min}$ … minimum temperature; $T_{max}$ … maximum temperature

**Results of analyses with three additional types of thermophilization rates**

To ensure the robustness of our results we repeated our analyses using thermophilization rates calculated from plot-level changes in mean elevational ranks (i.e. not cover-weighted), mean ecological indicator values for temperature (EIV T; Dengler et al. 2023), and cover-weighted mean EIV T. Thermophilization rates based on elevational ranks and EIV T were available for 724 and 718 plots, respectively.

The mean thermophilization rate was positive and differed significantly from zero for all three types of thermophilization rates (Supplementary Table 11). The mean thermophilization rate per decade across all plots was 0.04 (SD = 0.14, min = -0.71, max = 1.07), 0.04 (SD = 0.14, min = -0.49, max = 0.86) and 0.07 (SD = 0.20, min = -0.82, max = 1.26) when calculated from mean elevational ranks, mean EIV T and cover-weighted mean EIV T, respectively. Of these plots, 60.8%, 62.0% and 65.6% showed a positive and 30.4%, 32.6% and 33.1% a negative thermophilization rate, while 8.8%, 5.4% and 1.3% showed a rate of zero, when calculated from mean elevational ranks, mean EIV T and cover-weighted mean EIV T, respectively (Supplementary Fig. 4).

**Supplementary Table 11.** Summaries of intercept-only linear mixed-effects models with either one of the three different types of thermophilization rate as response. The models included a random-intercept term for plot cluster nested in summit nested in mountain region. Only the one-sided, right-tailed p values are reported. Bold p values indicate statistical significance. Est. ... estimated intercept; S.E. ... standard error.

| Thermophilization rate | Est. | S.E. | z value | p value |
|---|---|---|---|---|
| Mean elevational rank | 0.046 | 0.010 | 4.496 | **< 0.001** |
| Mean EIV T | 0.041 | 0.009 | 4.698 | **< 0.001** |
| Cover-weighted mean EIV T | 0.069 | 0.014 | 5.063 | **< 0.001** |

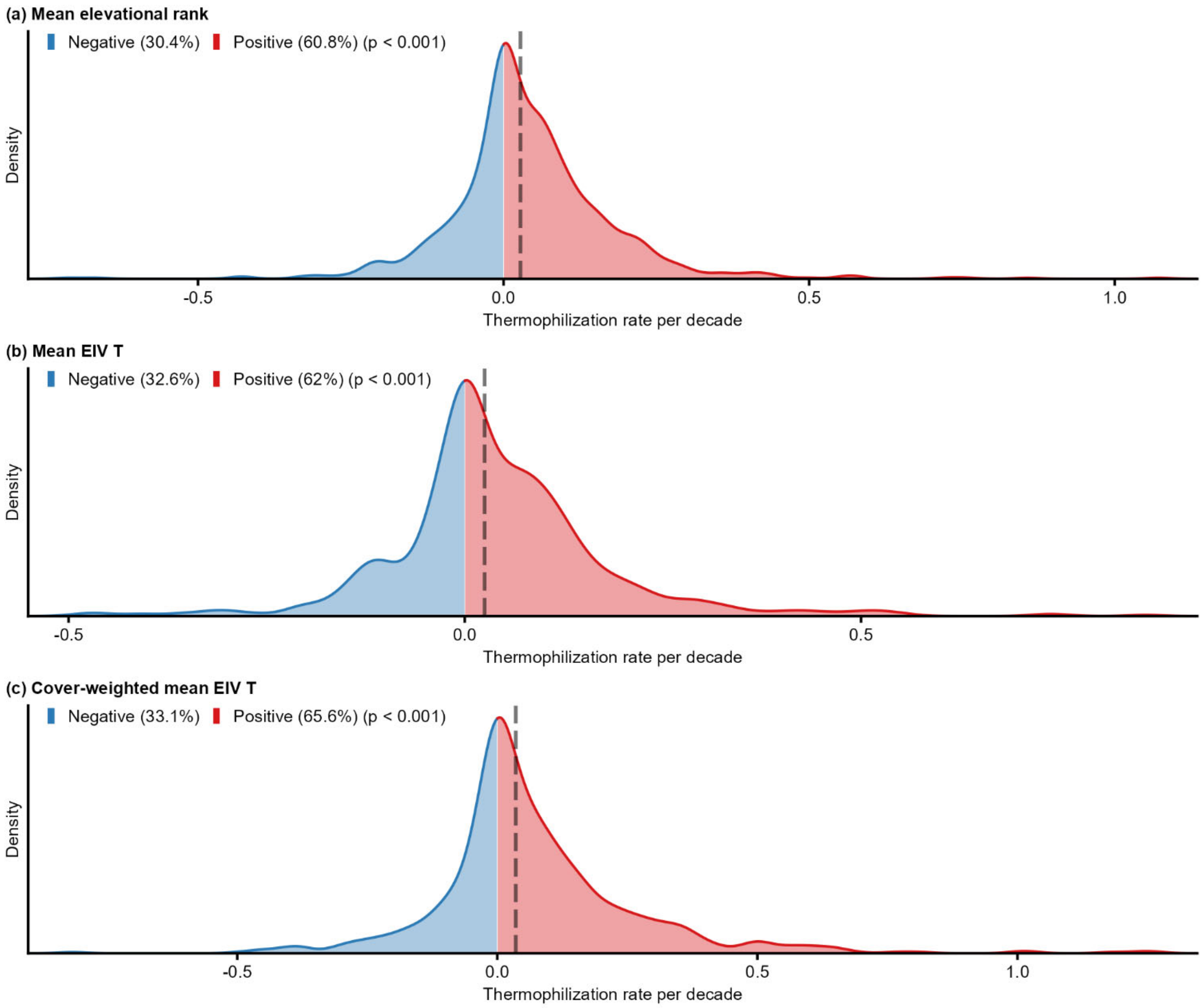


**Supplementary Fig. 4. Distribution of additional types of thermophilization rates.** Distribution of plot-level thermophilization rates calculated from (a) mean elevational ranks (n = 724), (b) mean EIV T (n = 718) and (c) cover-weighted mean EIV T (n = 718). Percentages indicate proportions of negative (blue) and positive (red) rates. The dashed lines indicate the respective medians. Values do not sum up to 100% due to plots with thermophilization rates of zero (8.8%, 5.4% and 1.3% for mean elevational ranks, mean EIV T and cover-weighted mean EIV T, respectively). One-sided p values were derived from intercept-only linear mixed-effects models testing whether thermophilization rates and rates of temperature change were significantly greater than zero. The p values were further corrected for multiple comparisons (this adjustment can lead to p values of exactly 1) and are presented at the top of each panel.

Relating the three types of thermophilization rate to the summits' latitude, longitude and elevation we found a significant relationship only for the thermophilization rate calculated from mean elevational ranks along the latitudinal gradient, with higher thermophilization rates in the south compared to the north of Europe (Supplementary Table 12).

**Supplementary Table 12.** Summaries of linear mixed-effects models relating either of the three types of plot-level thermophilization rate to the summits' latitude (degrees), longitude (degrees) and elevation (expressed as the difference between the summits' elevations and the elevations of the current regional forestlines; expressed in kilometers to keep estimated effects interpretable). The models included a random-intercept term for plot cluster nested in summit nested in mountain region. Only the Bonferroni-adjusted, one-sided, right-tailed p values are reported (this adjustment can lead to p values of exactly 1). Bold p values indicate statistical significance. Est. ... estimated effect; S.E. ... standard error.

| Thermophilization rate | Predictor | Est. | S.E. | z value | p value |
|---|---|---|---|---|---|
| Mean elevational rank | Longitude | -0.008 | 0.009 | -0.903 | 0.366 |
| | Latitude | -0.020 | 0.009 | -2.319 | **0.020** |
| | Elevation | 0.006 | 0.009 | 0.640 | 0.522 |
| Mean EIV T | Longitude | -0.003 | 0.009 | -0.390 | 0.697 |
| | Latitude | -0.009 | 0.009 | -1.069 | 0.285 |
| | Elevation | 0.006 | 0.008 | 0.741 | 0.458 |
| Cover-weighted mean EIV T | Longitude | 0.011 | 0.014 | 0.779 | 0.436 |
| | Latitude | -0.007 | 0.014 | -0.536 | 0.592 |
| | Elevation | -0.001 | 0.013 | -0.105 | 0.916 |

Fitting linear mixed-effects models relating either of the three types of thermophilization rate to the rates of change in the ten temperature metrics on both climatic scales (i.e. macro- and microclimate) revealed significant effects of macroclimatic mean summer temperature on the thermophilization rate calculated from mean elevational ranks with a marginal $R^2$ ($R^2$m) of 0.02, as well as of macroclimatic mean and minimum winter temperatures on the thermophilization rate calculated from cover-weighted mean EIV T with $R^2$m values of 0.02 and 0.03, respectively. Across all temperature metrics, macroclimatic metrics explained only a minor fraction of the variation in thermophilization rate but still more than microclimatic ones (Supplementary Fig. 5, Supplementary Table 13).

As all previous analyses on the three different types of thermophilization rate revealed results and patterns qualitatively largely similar to the results presented in the main text, subsequent analyses (i.e. considering macroclimatic temperature changes with a 4-year time lag, introducing interactions between two temperature metrics, and introducing the three covariables availability of thermophilic colonizers, rock and scree cover, as well as bare soil cover as predictors into linear mixed-effects models) were not repeated for these three types of thermophilization rate.

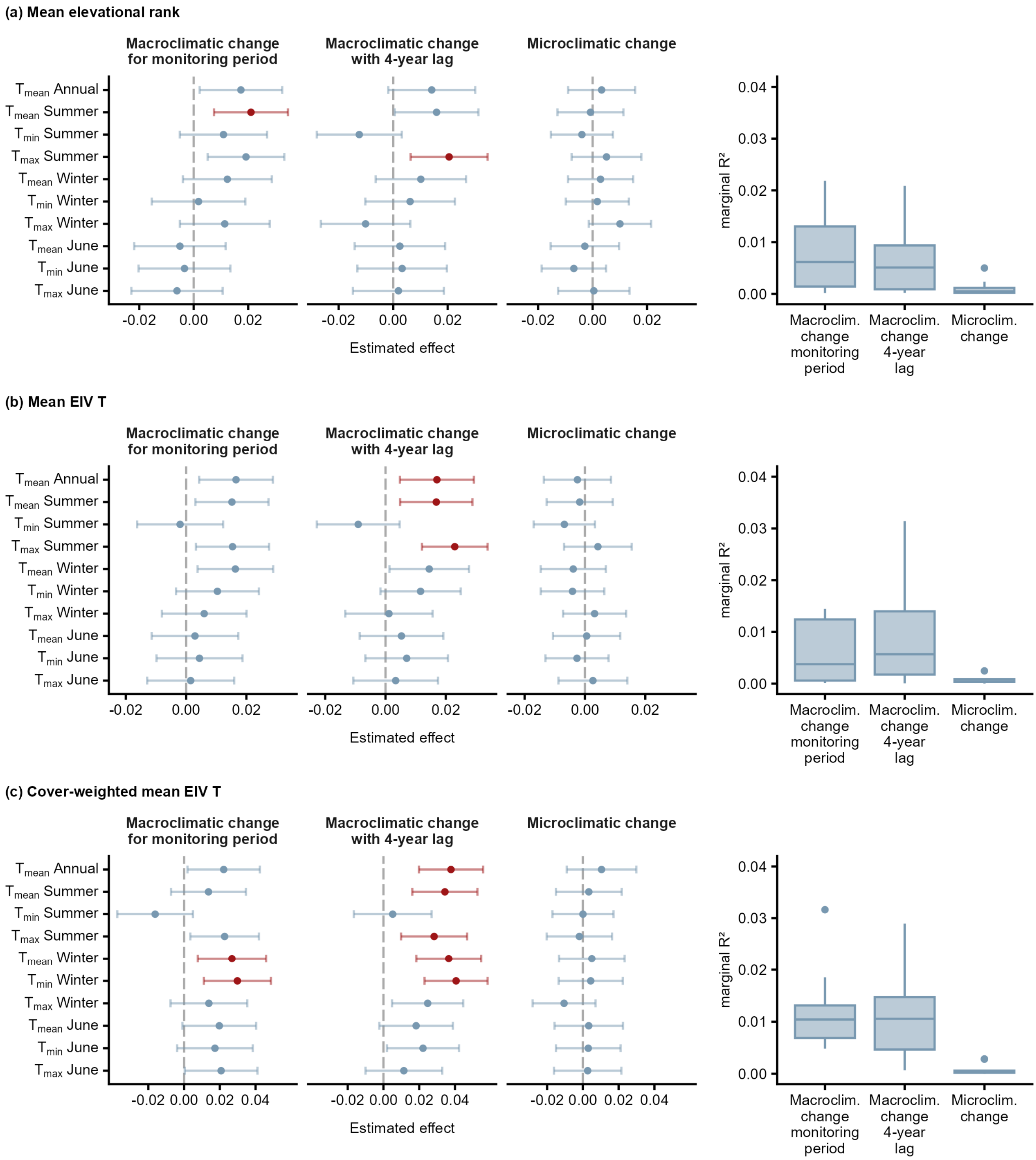


**Supplementary Fig. 5. Results of linear mixed-effects models relating additional types of thermophilization rates to rates of temperature change.** Estimated effects (dots) and 90% confidence intervals (error bars) of linear mixed-effect models relating the three types of thermophilization rate individually to the rates of change in each of the ten temperature metrics on both climatic scales (left). Temperature metrics with a significant effect on the thermophilization rate (after correcting for multiple testing) are highlighted in red. Estimated effects are based on models fitted with the rates of temperature change scaled to zero mean and unit variance for better comparability. $R^2$m values of models containing either one of the macro- or microclimatic temperature metrics (n = 10; right). Boxes indicate the median and interquartile range (IQR; 25th and 75th percentiles), whiskers extend to the furthest data points within 1.5 × IQR from the hinges, and points represent outliers.

**Supplementary Table 13.** Summaries of linear mixed-effects models relating either of the three types of plot-level thermophilization rate to the rates of changes in each of the ten temperature metrics on both climatic scales. The models included a random-intercept term for plot cluster nested in summit nested in mountain region. Scale indicates which version of the temperature metric was used (macro- or microclimatic). Only the one-sided, right-tailed p values adjusted for multiple comparisons are reported (this adjustment can lead to p values of exactly 1). Bold p values indicate statistical significance. Est. ... estimated effect; S.E. ... standard error; $R^2m$ ... marginal $R^2$; $T_{mean}$ ... mean temperature; $T_{min}$ ... minimum temperature; $T_{max}$ ... maximum temperature.

| Thermophilization rate | Predictor | Scale | Est. | S.E. | z value | p value | $R^2m$ |
|---|---|---|---|---|---|---|---|
| Mean elevational ranks | $T_{mean}$ Annual | macro | 0.017 | 0.009 | 1.881 | 0.122 | 0.015 |
| | | micro | 0.003 | 0.007 | 0.442 | 1.000 | 0.001 |
| | $T_{mean}$ Summer | macro | 0.021 | 0.008 | 2.547 | **0.034** | 0.022 |
| | | micro | -0.001 | 0.007 | -0.107 | 1.000 | 0.000 |
| | $T_{min}$ Summer | macro | 0.011 | 0.010 | 1.120 | 1.000 | 0.006 |
| | | micro | -0.004 | 0.007 | -0.570 | 1.000 | 0.001 |
| | $T_{max}$ Summer | macro | 0.019 | 0.009 | 2.243 | 0.083 | 0.018 |
| | | micro | 0.005 | 0.008 | 0.656 | 1.000 | 0.001 |
| | $T_{mean}$ Winter | macro | 0.012 | 0.010 | 1.245 | 0.395 | 0.008 |
| | | micro | 0.003 | 0.007 | 0.400 | 1.000 | 0.000 |
| | $T_{min}$ Winter | macro | 0.002 | 0.010 | 0.169 | 1.000 | 0.000 |
| | | micro | 0.002 | 0.007 | 0.245 | 1.000 | 0.000 |
| | $T_{max}$ Winter | macro | 0.011 | 0.010 | 1.137 | 0.865 | 0.006 |
| | | micro | 0.010 | 0.007 | 1.445 | 0.579 | 0.005 |
| | $T_{mean}$ June | macro | -0.005 | 0.010 | -0.496 | 1.000 | 0.001 |
| | | micro | -0.003 | 0.008 | -0.374 | 1.000 | 0.000 |
| | $T_{min}$ June | macro | -0.003 | 0.010 | -0.331 | 1.000 | 0.001 |
| | | micro | -0.007 | 0.007 | -0.958 | 1.000 | 0.002 |
| | $T_{max}$ June | macro | -0.006 | 0.010 | -0.603 | 1.000 | 0.002 |
| | | micro | 0.000 | 0.008 | 0.056 | 1.000 | 0.000 |
| Mean EIV T | $T_{mean}$ Annual | macro | 0.017 | 0.007 | 2.229 | 0.053 | 0.014 |
| | | micro | -0.003 | 0.007 | -0.370 | 1.000 | 0.000 |
| | $T_{mean}$ Summer | macro | 0.015 | 0.007 | 2.061 | 0.121 | 0.012 |
| | | micro | -0.002 | 0.007 | -0.263 | 1.000 | 0.000 |
| | $T_{min}$ Summer | macro | -0.002 | 0.009 | -0.228 | 1.000 | 0.000 |
| | | micro | -0.007 | 0.006 | -1.107 | 1.000 | 0.002 |
| | $T_{max}$ Summer | macro | 0.015 | 0.007 | 2.090 | 0.123 | 0.012 |
| | | micro | 0.004 | 0.007 | 0.629 | 1.000 | 0.001 |
| | $T_{mean}$ Winter | macro | 0.016 | 0.008 | 2.143 | 0.059 | 0.014 |
| | | micro | -0.004 | 0.007 | -0.593 | 1.000 | 0.001 |
| | $T_{min}$ Winter | macro | 0.010 | 0.008 | 1.243 | 0.400 | 0.006 |
| | | micro | -0.004 | 0.006 | -0.645 | 1.000 | 0.001 |
| | $T_{max}$ Winter | macro | 0.006 | 0.009 | 0.704 | 1.000 | 0.002 |
| | | micro | 0.003 | 0.006 | 0.501 | 1.000 | 0.001 |
| | $T_{mean}$ June | macro | 0.003 | 0.009 | 0.338 | 1.000 | 0.000 |
| | | micro | 0.001 | 0.007 | 0.083 | 1.000 | 0.000 |
| | $T_{min}$ June | macro | 0.004 | 0.009 | 0.513 | 1.000 | 0.001 |
| | | micro | -0.003 | 0.006 | -0.417 | 1.000 | 0.000 |
| | $T_{max}$ June | macro | 0.002 | 0.009 | 0.174 | 1.000 | 0.000 |
| | | micro | 0.003 | 0.007 | 0.379 | 1.000 | 0.000 |

**Supplementary Table 13.** Continued.

| | | | | | | | |
|---|---|---|---|---|---|---|---|
| Cover-weighted mean EIV T | $T_{mean}$ Annual | macro | 0.022 | 0.012 | 1.805 | 0.145 | 0.013 |
| | | micro | 0.010 | 0.012 | 0.881 | 1.000 | 0.003 |
| | $T_{mean}$ Summer | macro | 0.014 | 0.013 | 1.074 | 0.875 | 0.005 |
| | | micro | 0.003 | 0.011 | 0.293 | 1.000 | 0.000 |
| | $T_{min}$ Summer | macro | -0.016 | 0.013 | -1.257 | 1.000 | 0.007 |
| | | micro | 0.000 | 0.010 | 0.002 | 1.000 | 0.000 |
| | $T_{max}$ Summer | macro | 0.023 | 0.012 | 1.947 | 0.173 | 0.013 |
| | | micro | -0.002 | 0.011 | -0.180 | 1.000 | 0.000 |
| | $T_{mean}$ Winter | macro | 0.027 | 0.012 | 2.310 | **0.039** | 0.019 |
| | | micro | 0.005 | 0.011 | 0.447 | 1.000 | 0.001 |
| | $T_{min}$ Winter | macro | 0.030 | 0.011 | 2.621 | **0.016** | 0.032 |
| | | micro | 0.004 | 0.011 | 0.400 | 1.000 | 0.000 |
| | $T_{max}$ Winter | macro | 0.014 | 0.013 | 1.065 | 0.969 | 0.005 |
| | | micro | -0.011 | 0.011 | -0.987 | 1.000 | 0.003 |
| | $T_{mean}$ June | macro | 0.020 | 0.013 | 1.575 | 0.234 | 0.010 |
| | | micro | 0.003 | 0.012 | 0.279 | 1.000 | 0.000 |
| | $T_{min}$ June | macro | 0.017 | 0.013 | 1.346 | 0.358 | 0.008 |
| | | micro | 0.003 | 0.011 | 0.272 | 1.000 | 0.000 |
| | $T_{max}$ June | macro | 0.021 | 0.012 | 1.680 | 0.199 | 0.011 |
| | | micro | 0.003 | 0.011 | 0.232 | 1.000 | 0.000 |
