# Supplementary material for: Widespread thermophilization but weak link to climate warming in Europe’s summit plant communities
Source: Nat Ecol Evol. 2026 Aug 14;10(10):1838–51. doi: 10.1038/s41559-026-03150-x (PMC13642035; doi:10.1038/s41559-026-03150-x)
Supplement: Supplementary file 2 [file 41559_2026_3150_MOESM2_ESM.pdf]

nature portfolio

Corresponding author(s): Johannes Hausharter

Last updated by author(s): May 5, 2026

Reporting Summary

Nature Portfolio wishes to improve the reproducibility of the work that we publish. This form provides structure for consistency and transparency in reporting. For further information on Nature Portfolio policies, see our Editorial Policies and the Editorial Policy Checklist.

Statistics

For all statistical analyses, confirm that the following items are present in the figure legend, table legend, main text, or Methods section.

n/a	Confirmed	
☐	☒	The exact sample size (n) for each experimental group/condition, given as a discrete number and unit of measurement
☒	☐	A statement on whether measurements were taken from distinct samples or whether the same sample was measured repeatedly
☐	☒	The statistical test(s) used AND whether they are one- or two-sided<br>*Only common tests should be described solely by name; describe more complex techniques in the Methods section.*
☐	☒	A description of all covariates tested
☐	☒	A description of any assumptions or corrections, such as tests of normality and adjustment for multiple comparisons
☐	☒	A full description of the statistical parameters including central tendency (e.g. means) or other basic estimates (e.g. regression coefficient) AND variation (e.g. standard deviation) or associated estimates of uncertainty (e.g. confidence intervals)
☐	☒	For null hypothesis testing, the test statistic (e.g. F, t, r) with confidence intervals, effect sizes, degrees of freedom and P value noted<br>*Give P values as exact values whenever suitable.*
☒	☐	For Bayesian analysis, information on the choice of priors and Markov chain Monte Carlo settings
☒	☐	For hierarchical and complex designs, identification of the appropriate level for tests and full reporting of outcomes
☐	☒	Estimates of effect sizes (e.g. Cohen's d, Pearson's r), indicating how they were calculated

Our web collection on statistics for biologists contains articles on many of the points above.

Software and code

Policy information about availability of computer code

Data collection	Much of the data used in this study were collected directly in the field without the use of any software; however, some data was generated using QGIS Desktop (Version 3.34.11) and R (Version 4.5.0; using the RStudio IDE, Version 2026.01.0+392).
Data analysis	Data were analyzed using R (Version 4.5.0; using the RStudio IDE, Version 2026.01.0+392). The following R packages were used for analyzing the data: WorldFlora (Version 1.14-5; Kindt 2020), glmmTMB (Version 1.1.14; Brooks et al. 2017), performance (Version 0.17.0; Lüdecke et al. 2021).<br>Upon acceptance the data and code can be accessed via https://doi.org/ 10.6084/m9.figshare.32665953. For now, this DOI is reserved and will be made active upon acceptance. In the meantime, the repository can be accessed via a temporary, private link: https://figshare.com/ s/583b83f9493129e68350

For manuscripts utilizing custom algorithms or software that are central to the research but not yet described in published literature, software must be made available to editors and reviewers. We strongly encourage code deposition in a community repository (e.g. GitHub). See the Nature Portfolio guidelines for submitting code & software for further information.

Data

Policy information about availability of data

All manuscripts must include a data availability statement. This statement should provide the following information, where applicable:

- Accession codes, unique identifiers, or web links for publicly available datasets
- A description of any restrictions on data availability
- For clinical datasets or third party data, please ensure that the statement adheres to our policy

Upon acceptance the data and code can be accessed via https://doi.org/ 10.6084/m9.figshare.32665953. For now, this DOI is reserved and will be made active upon acceptance. In the meantime, the repository can be accessed via a temporary, private link: https://figshare.com/s/583b83f9493129e68350

Research involving human participants, their data, or biological material

Policy information about studies with human participants or human data. See also policy information about sex, gender (identity/presentation), and sexual orientation and race, ethnicity and racism.

Reporting on sex and gender	*Use the terms sex (biological attribute) and gender (shaped by social and cultural circumstances) carefully in order to avoid confusing both terms. Indicate if findings apply to only one sex or gender; describe whether sex and gender were considered in study design; whether sex and/or gender was determined based on self-reporting or assigned and methods used. Provide in the source data disaggregated sex and gender data, where this information has been collected, and if consent has been obtained for sharing of individual-level data; provide overall numbers in this Reporting Summary. Please state if this information has not been collected. Report sex- and gender-based analyses where performed, justify reasons for lack of sex- and gender-based analysis.*
Reporting on race, ethnicity, or other socially relevant groupings	*Please specify the socially constructed or socially relevant categorization variable(s) used in your manuscript and explain why they were used. Please note that such variables should not be used as proxies for other socially constructed/relevant variables (for example, race or ethnicity should not be used as a proxy for socioeconomic status). Provide clear definitions of the relevant terms used, how they were provided (by the participants/respondents, the researchers, or third parties), and the method(s) used to classify people into the different categories (e.g. self-report, census or administrative data, social media data, etc.) Please provide details about how you controlled for confounding variables in your analyses.*
Population characteristics	*Describe the covariate-relevant population characteristics of the human research participants (e.g. age, genotypic information, past and current diagnosis and treatment categories). If you filled out the behavioural & social sciences study design questions and have nothing to add here, write "See above."*
Recruitment	*Describe how participants were recruited. Outline any potential self-selection bias or other biases that may be present and how these are likely to impact results.*
Ethics oversight	*Identify the organization(s) that approved the study protocol.*

Note that full information on the approval of the study protocol must also be provided in the manuscript.

Field-specific reporting

Please select the one below that is the best fit for your research. If you are not sure, read the appropriate sections before making your selection.

☒ Life sciences ☐ Behavioural & social sciences ☐ Ecological, evolutionary & environmental sciences

For a reference copy of the document with all sections, see nature.com/documents/nr-reporting-summary-flat.pdf

Life sciences study design

All studies must disclose on these points even when the disclosure is negative.

Sample size	Sample size was predetermined due to this study using data collected by the systematic GLORIA monitoring scheme. Sample size therefore depended on the number of vegetation plots surveyed by GLORIA since 2001. 724 vegetation plots could be analyzed in this study as these were the plots for which we had complete lists of the taxa occurring in them, as well as sufficient temperature logger data (spanning at least 10 years). One analyses (the one also considering non-climatic covariables as predictors for thermophilization rates) was restricted to 720 plots, as one of the covariable could not be calculated for four of the plots. We consider 724 (or 720) plots surveyed for more than 20 years each and spread across Europe a sufficient sample, in terms of sample size, as well as temporal and spatial coverage. Across such a large temporal and spatial extent, this sample size should be sufficient to reveal temperature change effects on vegetation.
Data exclusions	Data for specific vegetation plots were excluded if the corresponding soil-temperature data loggers did not provide a sufficiently long temperature time series. As sufficient we considered a temperature time series spanning at least 10 years. Reasons for insufficient temperature time series: logger surfacing or malfunctioning.
Replication	Not applicable, as we analyze long-term vegetation monitoring data collected between 2001 and 2022 for which replication is not possible

Replication	retroactively.
Randomization	Not applicable, as we analyze long-term vegetation monitoring data without any allocation into experimental groups. One could consider individual summits or mountain regions as experimental groups, but in this case the allocation to these "groups" is determined by the location where the recorded plants occurred.
Blinding	Not applicable, as we analyze long-term vegetation monitoring data that was already collected. The only aspect where blinding would have been relevant was the visual estimation of the individual plant species' percentage covers in the vegetation plots; however, the GLORIA monitoring scheme does not consider blinding for cover estimation.

Reporting for specific materials, systems and methods

We require information from authors about some types of materials, experimental systems and methods used in many studies. Here, indicate whether each material, system or method listed is relevant to your study. If you are not sure if a list item applies to your research, read the appropriate section before selecting a response.

Materials & experimental systems

n/a	Involved in the study
☒	☐ Antibodies
☒	☐ Eukaryotic cell lines
☒	☐ Palaeontology and archaeology
☒	☐ Animals and other organisms
☒	☐ Clinical data
☒	☐ Dual use research of concern
☒	☐ Plants

Methods

n/a	Involved in the study
☒	☐ ChIP-seq
☒	☐ Flow cytometry
☒	☐ MRI-based neuroimaging

Plants

Seed stocks	*Report on the source of all seed stocks or other plant material used. If applicable, state the seed stock centre and catalogue number. If plant specimens were collected from the field, describe the collection location, date and sampling procedures.*
Novel plant genotypes	*Describe the methods by which all novel plant genotypes were produced. This includes those generated by transgenic approaches, gene editing, chemical/radiation-based mutagenesis and hybridization. For transgenic lines, describe the transformation method, the number of independent lines analyzed and the generation upon which experiments were performed. For gene-edited lines, describe the editor used, the endogenous sequence targeted for editing, the targeting guide RNA sequence (if applicable) and how the editor was applied.*
Authentication	*Describe any authentication procedures for each seed stock used or novel genotype generated. Describe any experiments used to assess the effect of a mutation and, where applicable, how potential secondary effects (e.g. second site T-DNA insertions, mosiacism, off-target gene editing) were examined.*
