# Supplementary material for: Widespread thermophilization but weak link to climate warming in Europe’s summit plant communities
Source: Nat Ecol Evol. 2026 Aug 14;10(10):1838–51. doi: 10.1038/s41559-026-03150-x (PMC13642035; doi:10.1038/s41559-026-03150-x)
Supplement: Supplementary file 3 [file 41559_2026_3150_MOESM3_ESM.pdf]

nature portfolio

Peer Review File

Widespread thermophilization but weak link to climate warming in Europe's summit plant communities

Corresponding Author: Mr Johannes Hausharter

This file contains all reviewer reports in order by version, followed by all author rebuttals in order by version.

Version 0:

Reviewer comments:

Reviewer #1

(Remarks to the Author)
This work brings together vegetation data spanning both a large spatial and temporal (~20 years) scale to investigate the drivers of thermophilization observed in mountain systems. Additionally, both macro- and micro-climate data are considered as predictors of rate of thermophilzation, as well as plot-level estimates of the substrate and surrounding vegetation. This is a rich dataset, and a key one to address questions about the importance of the ecological context for how plant communities may respond to climate change.
I appreciate the clear hypotheses setup in the introduction (L184 - 193), however I think they need to be stated earlier in the introduction to give some conceptual bearing to the range of variables quantified described in the previous section. Currently, the paragraph briefly describing the methods is difficult to follow and the individual sentences describe unrelated variables. The text in L157 – 162 could also be separated out and used to distinguish between what variables were used for the separate questions of the manuscript.
Related to this, there needs to be more clarity in what sections of the results are directly related to the hypotheses tested. I would encourage directly tying each section to a specific hypothesis. For example, the section on elevation and geographical gradients (L252) was not described in the introduction, making the results section more difficult to follow.
In the results, the model structures used to analyze the patterns are difficult to follow (L282-285, L254-255). These models are more clearly described in the methods, but the reference here to the model structure with unclear language also unnecessarily complicates the results section.
I was surprised that the additive or interactive effects of the climate variables for predicting the rate of thermophilzation were not considered. In examining the methods, supplemental I believe that all climate variable effects were modeled with separate linear mixed-effects models. Please include an explanation of why the climate variables were modeled separately as opposed to as additive or interactive predictors. Further, as described in L622, I believe the non-climatic predictors (eg., surrounding species pool) were modeled only as additive predictors, not including interactions with the climate variables. I think that looking for interactions between the climate variables and these non-climatic components would allow the work to better address the question if these local ecological contexts mediate how communities are responding to changing conditions.
Minor lines-specific comments below:
L146 – Remove 'actually'
L160 – the types of temperature data used is difficult to follow in this sentence – I suggest removing the commas
L180 – L183 The comparison that allows for the calculation 'of the difference' is not clear. I would add here that is based on only the initial survey, and otherwise confusing why not also a dynamic metric.
Figure 1. I think it would be helpful to Include the underlying data, not just fit lines for panel b
Figure 2. This could possibly be in the supplement. The novel component of this paper is how the thermophilzation trends covaries with rates of change in climate conditions, which can't be gleaned from this figure.
L122. How were the focal temperature metrics chosen? Why were interactions between climate variables excluded? What about snow (L389)? The length of snow cover can be pulled from the logger data (using days with minimum diurnal variability), even if not possible to derive for the macroclimate-level. Why was degree-days not included as it can contain some of the interactive effects of multiple seasonal temperature metrics?
Figure 4. Overlaying the density plots (with decreases alpha) may allow easier comparison across the effect size of the different predictors
Figure S3 may be worth highlighting in the main text for a subset of the climate variables considered

Reviewer #2

(Remarks to the Author)
This is a valuable contribution summarizing the changes for plants on mountain summits across Europe. It demonstrates an expected thermophilization of the flora on these summits, but this thermophilization is surprisingly not related to the temperature changes. In my opinion the paper is especially interesting because it uses a range of relevant temperature estimates, which perhaps becomes especially important given the lack of a clear relationship between thermophilization and temperature change. I found the inclusion of both macroclimatic and microclimatic variables to be especially interesting in this context.
One potential problem with the study is that it only has data back to 2001. This does not take away the value of the observed rate of thermophilization or the observed temperature changes by itself, but merely the somewhat naïve expectation that the thermophilization in the period 2001-2022 is related to the climate change in the same period. The floral composition of a summit is not the result of the temperature of the sampling year, and hence the composition of the plots sampled are more a result of the temperature of the previous years. Ideally, one should therefore have temperature measurements available for a time period prior to 2001 (e.g. a five- or ten-year period from 2001 and backwards). This is of course not feasible for the microclimati estimates, but it could probably be estimated for the macroclimatical temperature estimates. I would therefore very much like to see if it would improve the explanatory power of temperature change if the rate of temperature change is estimated by for example 5-year averages (i.e. relating the thermophilization rate between 2001 and 2022 with a temperature change from 1997 -2022, or more explicitly relate the starting year to the 5-year average of the period 1997-2001). To keep the comparison with the microclimatic explanatory power, I would suggest that these analyses complement the current ones and not replace the macroclimatic analyses.
Apart from this point I noted only some minor issues especially around ambiguities in the methods used and the interpretability when only reading the main text.
Line 171 and Line 212: Information about the elevational rank is not sufficient to make a reasonable interpretation as a reader. After reading this the number of 0.06 units of elevational rank is probably not understandable for most readers. I would also say that the explanations in the methods are somewhat minimal (Lines 454-466). This description needs to be made more independent of the Gottfried et al description from 2012. I note, for example, that Gottfried et al had 6 levels and here you use 7. No references for the ranks are given in Table S5.
Line 175-178: I would like to see a reference to a table or list of the actual variables used.
Line 192: Not sure if I would expect an increase in thermophilization in areas with high cover of bare soil. Bare soil would to me in many cases indicate rougher climatic conditions (e.g. wind conditions).
Line 162-165: There are 53 summits each with 4 plots in each of the four cardinal directions. This makes up 848 plots deviating from the 724 plots used. This deviance makes it difficult to understand the sampling scheme, without some more explanations, or at lest saying that some plots/summits were discarded (with a reference to further information in the method section).
Given the confusion of the sampling protocol, I dd not find Figure 1b helpful
Line 210: What are the null models referring to here? It is understandable what is meant after reading the whole manuscript, but I found the 'null' here only to be confusing
Line 214-215: The information about the magnitude of elevational shift is interesting (0.06 vegetation belts per decade), but some more context about the levels of vegetation belts are needed for this result to make meaning for the reader.
Line 225-230: The text focuses on the variability of the climatic estimates. But are these more interesting than the actual changes estimated. I would say that the mean changes are the most interesting, and would therefore recommend that the text focuses on the mean differences in this part.
Line 246-252: I would recommend focusing first on the thermophilization rate, and then on temperature change. The first sentence describes a model including both thermophilization and temperature change but only report on the former. I
Figure 4: I am not sure if this figure is needed in the main document. My feeling is that the main message of this figure is equally well conveyed in the text, and that the figure did not really help in making the message clearer. Perhaps rather replace this with a direct relationship between thermophilization and availability of colonizers and rock/scree cover.
Table S2: Is the p-value of 1.000 for the macroclimatic min summer temperature correct?
Table S5: Is there a reference for where these ERs come from?

Version 1:

Reviewer comments:

Reviewer #1

(Remarks to the Author)
I appreciate the inclusion of the additional analyses from both my and the other reviewers comments. I now only have minor comments on the current version of the abstract.
L108 In this following paragraph, it seems worth mentioning interactions between climate variables and time lags as these analyses are now included in the work. In particular, the reasoning for the inclusions due to alpine plant biology would be helpful. I think that these concepts could added most easily if the sentences concerning non-temperature drivers of change (starting at L129) is made into a separate paragraph
L145 I would suggest changing 'cold-adapted' and 'warm-demanding' to 'cold-associated' and warm-associated' species to be more accurate with how this species-level metric is quantified
Figure 1 legends need to specify how to interpret the change in cover-weighted mean elevation rate is linked to

thermophilzation trends – i.e., positive trends indicate thermophilzation. This may be obvious for researchers that commonly used the elevation rank system, but adding this explanation would make the figure more generally interpretable.
L328 this sentence is difficult to follow, please simplify.
L 445 The dynamics described here and in this section overall are explored (albeit in a different ecosystem type) in Ackerly et al 2020 https://esajournals.onlinelibrary.wiley.com/doi/10.1002/fee.2204, which might be useful additional citation in this section
L627 – 623 Distance to forest line is not used in the main text, so I think that the description of this metric can be removed from the methods . See also L679

Reviewer #2

(Remarks to the Author)
The authors have done an excellent job revising the manuscript, and this remains a valuable contribution based on an important data set. I am very pleased with the authors' responses the comments on the previous version, and believe that particularly the inclusion of delayed responses and interactions between predictor variables are valuable additions and have been handled well.

Open Access This Peer Review File is licensed under a Creative Commons Attribution 4.0 International License, which permits use, sharing, adaptation, distribution and reproduction in any medium or format, as long as you give appropriate credit to the original author(s) and the source, provide a link to the Creative Commons license, and indicate if changes were made.
In cases where reviewers are anonymous, credit should be given to 'Anonymous Referee' and the source.
The images or other third party material in this Peer Review File are included in the article's Creative Commons license, unless indicated otherwise in a credit line to the material. If material is not included in the article's Creative Commons license and your intended use is not permitted by statutory regulation or exceeds the permitted use, you will need to obtain permission directly from the copyright holder.
To view a copy of this license, visit https://creativecommons.org/licenses/by/4.0/

Reviewers' remarks to the authors and the authors' responses

Our responses are formatted in blue, to allow for easier differentiation between reviewer remarks (in black) and our responses. The line numbers we indicate in our responses refer to the revised document without tracked changes.

Reviewer #1

This work brings together vegetation data spanning both a large spatial and temporal (~20 years) scale to investigate the drivers of thermophilization observed in mountain systems. Additionally, both macro- and micro-climate data are considered as predictors of rate of thermophilzation, as well as plot-level estimates of the substrate and surrounding vegetation. This is a rich dataset, and a key one to address questions about the importance of the ecological context for how plant communities may respond to climate change.

I appreciate the clear hypotheses setup in the introduction (L184 - 193), however I think they need to be stated earlier in the introduction to give some conceptual bearing to the range of variables quantified described in the previous section. Currently, the paragraph briefly describing the methods is difficult to follow and the individual sentences describe unrelated variables. The text in L157 – 162 could also be separated out and used to distinguish between what variables were used for the separate questions of the manuscript.

We split the long paragraph (starting at L154) preceding the hypothesis into two parts and inserted the paragraph that sets up our hypotheses in between (L167–176) the two resulting paragraphs. For now, we did not further separate the text in L161–166, as we hope that the text now flows and sets up the considered variables better, making the descriptions in the preceding paragraph easier to follow. We moreover shifted the sentence on possible effects of dispersal limitation to an earlier point in the introduction (L137–140) and inserted a sentence on the possible effects of substrate conditions (L140–141) to let the subsequent, related hypothesis appear better motivated to the reader. Finally, we added information on the statistical models used to the methods description within the introduction (L191–193, L202–204, L211–214) to make it easier for the reader to follow the results section.

Related to this, there needs to be more clarity in what sections of the results are directly related to the hypotheses tested. I would encourage directly tying each section to a specific hypothesis. For example, the section on elevation and geographical gradients (L252) was not described in the introduction, making the results section more difficult to follow.

We shifted the section on geographical and elevational gradients to the supporting information (in front of the table where we already presented the detailed summaries of these models (section *Geographical and elevational trends*; Tab. S10) because they are not essential for the paper. These analyses are still explained in the methods section, but now with a reference (L681) to the results in this updated section in the supporting information. The remaining three subsections within the results section are directly related to our stated hypotheses.

In the results, the model structures used to analyze the patterns are difficult to follow (L282-285, L254-255). These models are more clearly described in the methods, but the reference here to the model structure with unclear language also unnecessarily complicates the results section.

We tried to improve our descriptions of the model structures (L268 & 328–335) without introducing too many details. We now also introduce the statistical models already at the end of the introduction (L191–193, L202–204, L211–214) to make the entire workflow easier to follow for the reader.

I was surprised that the additive or interactive effects of the climate variables for predicting the rate of thermophilzation were not considered. In examining the methods, supplemental I believe that all climate variable effects were modeled with separate linear mixed-effects models. Please include an explanation of why the climate variables were modeled separately as opposed to as additive or interactive predictors.

We were indeed only looking at univariate effects of temperature metrics because this was and still is common practice in most analyses of climate-effects on alpine vegetation (e.g. Gottfried et al. 2012, Steinbauer et al. 2018, Yue et al. 2026). However, we are grateful for this comment as we now also consider models with two metrics at the same scale (i.e. interactions only among either macroclimatic or microclimatic variables, but not between macro- and microclimatic variables) and their interactions as predictors. We explored all possible combinations of two variables that were not too highly correlated ($-0.5 \leq r \leq 0.5$). We found that these two-predictor models indeed improved the relationship between warming and thermophilization, even if the explained variation remained low. We have now integrated the results of these models in the results and discussion sections (L283–293, L406–415), present their marginal R^2 values in Figure 3b and describe the approach in the methods section (L691–697).

Further, as described in L622, I believe the non-climatic predictors (eg., surrounding species pool) were modeled only as additive predictors, not including interactions with the climate variables. I think that looking for interactions between the climate variables and these non-climatic components would allow the work to better address the question if these local ecological contexts mediate how communities are responding to changing conditions.

We again agree with the reviewer and now include these interactions. While they were largely irrelevant with respect to substrate conditions we found them to be significant with respect to the availability of thermophilic colonizers in most models: the effect of warming on thermophilization increased with the availability of such colonizers as one would expect. We also included these additional results into the manuscript in the results (L360–363) and discussion (L451–453 & 4461–463) sections and added new figures (Fig. 6 & Fig. S2) showing the described interaction. The part in the methods section describing these models was also updated to reflect the change (L723–727).

Minor lines-specific comments below:

L146 – Remove ‘actually’

We removed the word (L152) and modified the sentence.

L160 – the types of temperature data used is difficult to follow in this sentence – I suggest removing the commas

We removed the commas (L164).

L180 – L183 The comparison that allows for the calculation ‘of the difference’ is not clear. I would add here that is based on only the initial survey, and otherwise confusing why not also a dynamic metric.

We added the information that this difference was calculated for the first survey to the preceding sentence (L207–211).

Figure 1. I think it would be helpful to Include the underlying data, not just fit lines for panel b

We included the underlying data for all plots in Fig. 1b. We think this works well for the second and last plot, but maybe less so for the first one. Nevertheless, it still gives the reader an idea of the distribution of the actual values.

Figure 2. This could possibly be in the supplement. The novel component of this paper is how the thermophilzation trends covaries with rates of change in climate conditions, which can’t be gleaned from this figure.

While this is true, we still think that demonstrating thermophilization rates and their variability in the data, and in direct comparison with the temperature change rates and their variation, is not only worth it but necessary in a paper focused on the relationship between these two phenomena. We hence would like to keep this figure in the main text.

L122. How were the focal temperature metrics chosen? Why were interactions between climate variables excluded? What about snow (L389)? The length of snow cover can be pulled from the logger data (using days with minimum diurnal variability), even if not possible to derive for the macroclimate-level. Why was degree-days not included as it can contain some of the interactive effects of multiple seasonal temperature metrics?

We added a sentence to the introduction shortly mentioning which metrics were calculated and why (L197–202). We now also include interactions between climate variables (see our response to your previous remark concerning this point). Moreover, we calculated the number of days without snow cover (= minimum daily temperature variation) per year, as well as growing degree days from the temperature logger data. We introduced these two variables into our models, and they performed worse than most other temperature metrics, with no significant effect on thermophilization rates. However, we generally consider these variables to be less reliably calculated from our data. The logger data does contain measurement gaps, with considerable variation across individual loggers. These gaps are less of an issue when calculating the temporal trends in monthly means, minima and maxima, especially with our threshold of having at least 90% of hourly or daily values available for calculating these summary statistics. However, both snow cover duration and growing degree days are sums of daily values (counts of days or temperature sums) and hence more sensitive to these measurement gaps. We have therefore refrained from including the results of the models which included these variables in the paper.

Figure 4. Overlaying the density plots (with decreases alpha) may allow easier comparison across the effect size of the different predictors

We tried overlaying the density plots using different opacities, but due to the extensive overlap between the densities, the legibility suffered. We would therefore prefer to keep the densities separate.

Figure S3 may be worth highlighting in the main text for a subset of the climate variables considered

We shifted this figure to the extended data figures (Fig. E4) for it to be more prominent than it would be in the supporting information. As we already highlight the results of the models relating thermophilization rates to these different temperature change metrics (Fig. 3), we feel it would be redundant to also include these scatterplots in the main text.

Reviewer #2 (Remarks to the Author):

This is a valuable contribution summarizing the changes for plants on mountain summits across Europe. It demonstrates an expected thermophilization of the flora on these summits, but this thermophilization is surprisingly not related to the temperature changes. In my opinion the paper is especially interesting because it uses a range of relevant temperature estimates, which perhaps becomes especially important given the lack of a clear relationship between thermophilization and temperature change. I found the inclusion of both macroclimatic and microclimatic variables to be especially interesting in this context.

One potential problem with the study is that it only has data back to 2001. This does not take away the value of the observed rate of thermophilization or the observed temperature changes by itself, but merely the somewhat naïve expectation that the thermophilization in the period 2001-2022 is related to the climate change in the same period. The floral composition of a summit is not the result of the temperature of the sampling year, and hence the composition of the plots sampled are more a result of the temperature of the previous years. Ideally, one should therefore have temperature measurements available for a time period prior to 2001 (e.g. a five- or ten-year period from 2001 and backwards). This is of course not feasible for the microclimati estimates, but it could probably be estimated for the macroclimatical temperature estimates. I would therefore very much like to see if it would improve the explanatory power of temperature change if the rate of temperature change is estimated by for example 5-year averages (i.e. relating the thermophilization rate between 2001 and 2022 with a temperature change from 1997 -2022, or more explicitly relate the starting year to the 5-year average of the period 1997-2001). To keep the comparison with the microclimatic explanatory power, I would suggest that these analyses complement the current ones and not replace the macroclimatic analyses.

We are grateful for this comment. We have now evaluated how time-shifted rates of temperature change are related to thermophilization. To approach this in a systematic way we shifted the time window for which we calculated the macroclimatic temperature change year-by-year from 1 to 10 years earlier than the start of the monitoring period (i.e. from 2000 to 2021 to 1991 to 2012). We kept the length of time window constant to guarantee comparability of models fit with these different rates of temperature change subsequently. We found that this indeed had a significant effect on the relationship between warming and thermophilization rate, with models fit with temperature-change rates computed for 3 to 5 years earlier than the monitoring window delivering better results than those computed for the monitoring window itself. We hence repeated all analyses using the best-performing time-window for temperature-change computation and present the results of these analyses alongside our initial results. Please see the respective text in methods (L618–626), results (L275–282, L312–314, L337–340), discussion (starting at L398–406), extended data figures (Fig. E4–E7) and supporting information (Tab. S5, Tab. S8).

Apart from this point I noted only some minor issues especially around ambiguities in the methods used and the interpretability when only reading the main text.

Line 171 and Line 212: Information about the elevational rank is not sufficient to make a reasonable interpretation as a reader. After reading this the number of 0.06 units of elevational rank is probably not understandable for most readers. I would also say that the explanations in the methods are somewhat minimal (Lines 454-466). This description needs to be made more independent of the Gottfried et al description from 2012. I note, for example, that

Gottfried et al had 6 levels and here you use 7. No references for the ranks are given in Table S5.

We added additional descriptions to the parts of the introduction (L185–189), Results (L237–239), and Methods (L542–545), and hope that is now easier to interpret our results.

Line 175-178: I would like to see a reference to a table or list of the actual variables used.

Such a table is already provided in the supporting information and referred to in the methods section, but not the main text. We therefore added a sentence mentioning all temperature metrics that we calculated (L201).

Line 192: Not sure if I would expect an increase in thermophilization in areas with high cover of bare soil. Bare soil would to me in many cases indicate rougher climatic conditions (e.g. wind conditions).

We agree that bare soil is an ambivalent indicator as it might result from harsh conditions but also indicate suitable recruitment sites with low competition. We have thus added a respective sentence to the Discussion (L463–468).

Line 162-165: There are 53 summits each with 4 plots in each of the four cardinal directions. This makes up 848 plots deviating from the 724 plots used. This deviance makes it difficult to understand the sampling scheme, without some more explanations, or at lest saying that some plots/summits were discarded (with a reference to further information in the method section).

We highlight reasons for excluding certain plots in the methods section. We added a statement to the part you referenced (L179–181) highlighting the fact that not all plots could be considered and refer to the methods section for more details.

Given the confusion of the sampling protocol, I dd not find Figure 1b helpful

We hope that our improvements to the manuscript help to alleviate this issue.

Line 210: What are the null models referring to here? It is understandable what is meant after reading the whole manuscript, but I found the 'null' here only to be confusing

The "null model" is an intercept-only model. We changed how we refer to these models in the text by switching from "null model" to intercept-only model (e.g. L191–193, L234, L660–664).

Line 214-215: The information about the magnitude of elevational shift is interesting (0.06 vegetation belts per decade), but some more context about the levels of vegetation belts are needed for this result to make meaning for the reader.

We have added a respective description here and hope this is now better to understand (L237–239).

Line 225-230: The text focuses on the variability of the climatic estimates. But are these more interesting than the actual changes estimated. I would say that the mean changes are the most interesting, and would therefore recommend that the text focuses on the mean differences in this part.

We agree and have removed the part of the paragraph that focused on the variability within the different temperature change metrics.

Line 246-252: I would recommend focusing first on the thermophilization rate, and then on temperature change. The first sentence describes a model including both thermophilization and temperature change but only report on the former.

Following the suggestion of the other reviewer, we moved this section ("Geographical and elevational trends") into the supporting information, as these patterns are not really the focus of this study. In the moved section we clarify that separate models were fitted to either thermophilization rate or any individual variable describing rate of temperature change. Followed by first describing the results for the model of thermophilization rate and then describing the results for the models of the various temperature metrics.

Figure 4: I am not sure if this figure is needed in the main document. My feeling is that the main message of this figure is equally well conveyed in the text, and that the figure did not really help in making the message clearer. Perhaps rather replace this with a direct relationship between thermophilization and availability of colonizers and rock/scree cover.

We think this visual presentation of the results is an important summary of the multiple models that should remain in the main text because (1) the much stronger effect of the availability of thermophilic colonizers, as compared to the magnitude of temperature change is one of the main results of the paper, and (2) main results should be presented in visual items to emphasize them (also because many readers nowadays only look at Abstract and Figures and do not read the entire text). However, following the suggestion of the reviewer, we added another figure describing the interaction between change in temperature and availability of thermophilic colonizers which we think nicely complements our results (Fig. 6).

Table S2: Is the p-value of 1.000 for the macroclimatic min summer temperature correct?

Yes, this is a result of adjusting the p values for multiple comparisons. We added a clarifying statement to all tables in the supporting information where this is relevant (starting with the table you refer to, which is now Tab. S4).

Table S5: Is there a reference for where these ERs come from?

We added a short description and a reference to Gottfried et al. (2012) to the table caption (now Tab. S2).
